# Synthesis and Biological Evaluation of Novel 10-Substituted-7-ethyl-10-hydroxycamptothecin (SN-38) Prodrugs

**DOI:** 10.3390/molecules191219718

**Published:** 2014-11-27

**Authors:** Mo Zhou, Meixia Liu, Xinhua He, Hong Yu, Di Wu, Yishan Yao, Shiyong Fan, Ping Zhang, Weiguo Shi, Bohua Zhong

**Affiliations:** 1 Beijing Institute of Pharmacology & Toxicology, 27 Tai-Ping Road, Beijing 100850, China; E-Mails: michelle_zm2011@163.com (M.Z.); liumeixia@163.com (M.L.); hexinhua01@163.com (X.H.); spray_yao123456@hotmail.com (Y.Y.); fsyn1996@163.com (S.F.); gundnir@163.com (P.Z.); 2 Cell Biology Laboratory of Jilin Province Tumor Institute, No 1018 Huguang Road, Changchun 130012, China; E-Mail: yuhong123@163.com; 3 Tumor Centre, No.1 Hospital, Jilin University, 71 Xin-Min Street, Changchun 130012, China; E-Mail: wd78@163.com

**Keywords:** antitumor agent, camptothecins, prodrugs, SN-38

## Abstract

In an attempt to improve the antitumor activity and reduce the side effects of irinotecan (**2**), novel prodrugs of SN-38 (**3**) were prepared by conjugating amino acids or dipeptides to the 10-hydroxyl group of SN-38 via a carbamate linkage. The synthesized compounds completely generated SN-38 in pH 7.4 buffer or in human plasma, while remaining stable under acidic conditions. All prodrug compounds demonstrated much greater *in vitro* antitumor activities against HeLa cells and SGC-7901 cells than irinotecan. The most active compounds, **5h**, **7c**, **7d**, and **7f**, exhibited IC_50_ values that were 1000 times lower against HeLa cells and 30 times lower against SGC-7901 cells than those of irinotecan, and the inhibitory activities of these prodrugs against acetylcholinesterase (AchE) were significantly reduced, with IC_50_ values more than 6.8 times greater than that of irinotecan. In addition, compound **5e** exhibited the same level of tumor growth inhibitory activity as irinotecan (CPT-11) in a human colon xenograft model *in vivo*.

## 1. Introduction

**Figure 1:**
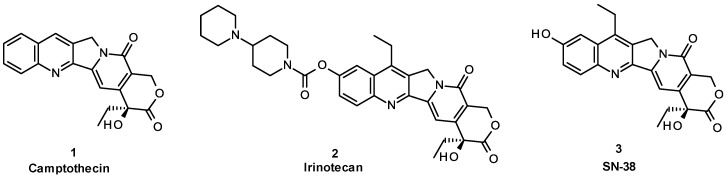
Structure of camptothecin and its analogs.

Camptothecin (CPT **1**, Figure 1) derivatives are potent topoisomerase I inhibitors with strong antitumor activities both *in vitro* and *in vivo* and are the only antitumor agents with topoisomerase I inhibitory activity used in the clinic [1,2].

Irinotecan (**2**) is a water-soluble prodrug of CPT derivative SN-38 (**3**). It is the most widely used CPT derivative for the treatment of colorectal cancer that has been previously treated with 5-fluorouracil; in addition, it has activity against a wide range of other cancers either as a single agent or in combination with other antitumor agents [3,4,5,6]. However, high individual variation in efficacy and toxicity as well as side effects, including acute cholinergic diarrhea, preclude its clinical use. It is believed that the low bioconversion efficiency (4%–5%) from irinotecan to the active form SN-38 is responsible for high interpatient variability in terms of the pharmacokinetics, which leads to considerable individual variation in efficacy and toxicity [7,8,9]. Moreover, the 4-piperidinopiperidine moiety of irinotecan is responsible for inhibiting acetylcholinesterase (AChE) activity, causing acute cholinergic diarrhea [10,11,12].

In this study, novel water soluble prodrugs of SN-38, with reduced AChE inhibitory activity, that could be completely converted to SN-38 were designed and synthesized. Linear amino acids or dipeptides with less steric effects were used to replace the 4-piperidinopiperidine moiety of irinotecan. Firstly, amino acids are considered to have well physiological compatibility and ability to penetrate the biological membrane by active transport, which make them the ideal prodrug carriers. Secondly, the carboxyl groups(-COOH) in amino acids or dipeptides can be easily converted to sodium or potassium salt to improve water solubility of prodrugs.The target compounds were prepared by conjugating an amino acid or dipeptide to the 10-hydroxyl group of SN-38 via a carbamate linker.

**Figure 2:**
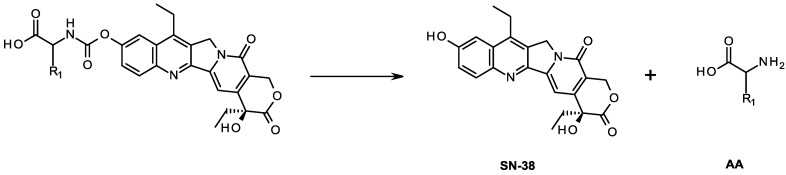
Conversion of target compound to SN-38.

These prodrugs were stable in aqueous solutions at acidic pH levels but were rapidly converted to active SN-38 in pH 7.4 buffer or in human plasma (Figure 2). These new CPT prodrugs exhibited much greater antitumor activity and less AChE inhibitory activity than irinotecan *in vitro*.

## 2. Results and Discussion

### 2.1. Chemistry

**Scheme 1:**
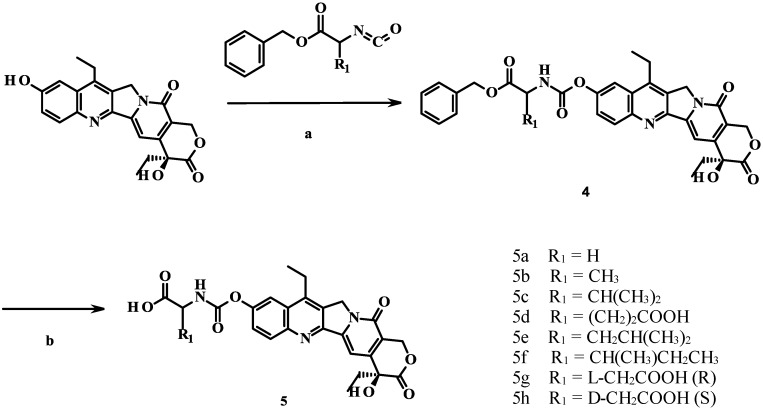
Synthesis of prodrugs **5a**–**h**.

The synthesis of the prodrugs is shown in Scheme 1. The benzyl ester of the amino acid was initially converted in good yield to the corresponding isocyanate in the presence of bis(trichloromethyl) carbonate. Next, the isocyanate was reacted with SN-38 to give coupled compounds **4** in 83%–89% yield. Deprotection of **4** by catalytic hydrogenation yielded compounds **5a**–**h**.

*Reagents and conditions*: (a) (i) Et_3_N, THF, rt, 30 min; (ii) isocyanate, THF, 40 °C, overnight; (b) Pd/C, H_2_, THF/EtOH, rt, overnight.

**Scheme 2:**
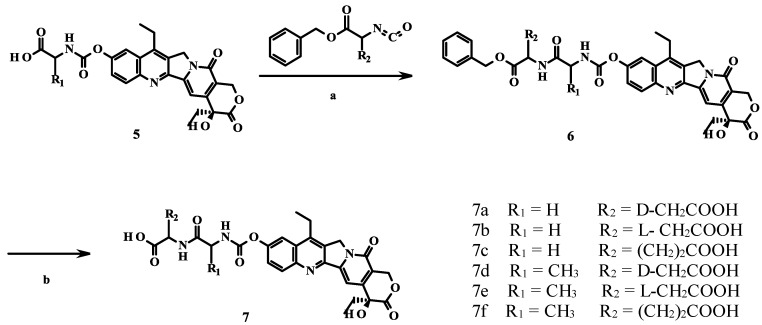
Synthesis of prodrugs **7a**–**f**.

Next, the dipeptide derivatives **7a**–**f** were synthesized by further conjugating the amino acid derivatives **5** with another amino acid in THF under room temperature for 24 h, as depicted in Scheme 2.

Reagents and conditions: (a) (i) Et_3_N, THF, rt, 30 min; (ii) isocyanate, THF, 40 °C, overnight; (b) Pd/C, H_2_, THF/EtOH, rt, overnight.

### 2.2. Cytotoxicity

The *in vitro* antitumor activities were evaluated on human cancer cell lines SGC-7901 and HeLa by the MTT assay, using irinotecan as the reference compound. The results are summarized in Table 1. The prodrugs **5a**–**h** and **7a**–**f** all exhibited much more potent antiproliferative activities against HeLa cells than irinotecan. Compounds **5e**–**5h** and **7b**–**7f** also showed greater antitumor activities against SGC-7901 cells than irinotecan. The most active compounds **5h**, **7c**, **7d**, and **7f** exhibited IC_50_ values that were less than 3.2 nM against HeLa cells, providing an approximately 1000-fold increase in potency compared to irinotecan. Meanwhile, they all showed antiproliferative activity that was 30-fold greater than that of irinotecan against SGC-7901 cells. Compared with the amino acid derivatives, the dipeptide derivatives exhibited greater antitumor activities.

**Table 1:** Antiproliferative activities of the derivatives against human cancer cell lines.

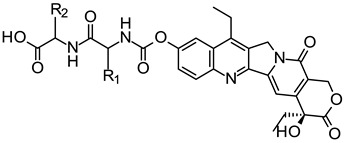				
Compound	R_1_		IC_50_ (μM)	
			SGC-7901	HeLa
**5a**	H		2.69 ± 0.11	(30.4 ± 47.0) × 10^−3^
**5b**	CH_3_		1.75 ± 0.60	(9.85 ± 11.1) × 10^−3^
**5c**	CH(CH_3_)_2_		1.88 ± 0.72	(5.61 ± 40.3) × 10^−3^
**5e**	CH_2_CH(CH_3_)_2_		0.50 ± 0.19	(1.82 ± 64.6) × 10^−3^
**5f**	CH(CH_3_)CH_2_CH_3_		0.76 ± 0.34	(3.64 ± 1.03) × 10^−3^
**5g**	CH_2_COOH (R)		0.98 ± 0.05	(9.07 ± 33.2) × 10^−3^
**5h**	d-CH_2_COOH (S)		0.24 ± 0.95	<(3.20 ± 64.3) × 10^−3^
Irinotecan			7.38 ± 1.24	1.32 ± 0.13
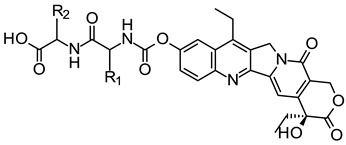				
**Compound**	**R_1_**	**R_2_**	**IC_50_ (μM)**	
			**SGC-7901**	**HeLa**
**7a**	H	d-CH_2_COOH	1.04 ± 0.90	<(3.20 ± 12.0) × 10^−3^
**7b**	H	l-CH_2_COOH	0.61 ± 0.68	(14.8 ± 135.0) × 10^−3^
**7c**	H	(CH_2_)_2_COOH	0.20 ± 0.07	<(3.20 ± 0.40) × 10^−3^
**7d**	CH3	d-CH_2_COOH	0.26 ± 0.64	<(3.20 ± 2.0) × 10^−3^
**7e**	CH3	l-CH_2_COOH	0.21 ± 0.55	(12.9 ± 133.0) × 10^−3^
**7f**	CH3	(CH_2_)_2_COOH	0.27 ± 0.18	(1.57 ± 3.10) × 10^−3^
Irinotecan			7.38 ± 1.24	1.32 ± 0.13

### 2.3. AChE Inhibition Assay

Irinotecan has been reported to be a potent inhibiter of AChE, and inhibition of this enzyme causes acute cholinergic diarrhea. Unlike irinotecan, the compounds we assayed were only weak inhibitors of AChE. As shown in Table 2, the IC_50_ value of irinotecan is 0.2 μM, while compounds **5a**, **5f**, **5g**, **5h**, **7a**, **7c**, **7d**, and **7f** exhibited IC_50_ values greater than 1.36 μM. One possible explanation is that the active site of AChE is present at the bottom of a gorge that is lined with hydrophobic amino acid residues [13]. The terminal dipiperidino moiety in CPT-11 that interacts with amino acid residues within AChE plays a major role in enzyme inhibition, which has been confirmed by X-ray crystallographic studies [14]. Hence, the hydrophobic side chain moiety of isoleucine in **5f** was attracted into the gorge where catalysis occurs, while the others with hydrophilic moiety had a lower binding affinity with it.

**Table 2:** AChE inhibitory activity.

Compound	IC_50_ (μM)	Compound	IC_50_ (μM)
**5a**	19.79	**7c**	>100
**5f**	1.36	**7d**	4.44
**5g**	3.08	**7f**	36.08
**5h**	7.43	Irinotecan	0.20
**7a**	115.7		

### 2.4. Stability and Conversion

The chemical stability of the prodrugs at pH 4.6 or 7.4 in PBS at 37 °C is summarized in Table 3. All the target compounds showed pH-dependent stability. They were stable at pH 4.6 at 37 °C for 12 h. Except for compound **5h**, less than 35% of the prodrugs remained after incubation for 12 h in pH 7.4 buffer. The dipepetide derivatives **7a**–**f** showed much less stability than the amino acid derivatives **5a**–**h**. For example, less than 30% of the prodrugs **7a**–**f** remained, while more than 85% of the prodrugs **5a**–**h** remained after incubation for 1 h in pH 7.4 buffer.

**Table 3:** The chemical stability of the prodrugs in pH 4.6 and pH 7.4 conditions.

Compound	Remaining Prodrugs (%)			
	1 h		12 h	
	pH 4.6	pH 7.4	pH 4.6	pH 7.4
**5a**	100	85.3	98.4	14.8
**5d**	98.1	85.2	92.5	8.6
**5g**	100	93.9	94.5	34.1
**5h**	100	93.4	94.5	52.2
**7a**	100	26.0	96.8	0
**7b**	100	17.6	96.9	0
**7c**	100	29.4	96.4	0
**7d**	100	26.3	95.5	0
**7e**	100	23.4	94.5	0
**7f**	100	14.4	94.7	0

The conversions of the prodrugs to SN-38 in human plasma are summarized in Table 4. All the compounds could be rapidly converted to their active form in plasma. Except for compound **5h**, the conversion rate of the prodrugs was more than 35% after incubation for 1 h and complete conversion was observed after incubation for 12 h in plasma. As at pH 7.4, the conversion rate of the dipeptide derivatives was faster than that of the amino acid derivatives.

**Table 4:** Conversion of the prodrugs to SN-38 in human plasma.

Compound	Conversion (%)		
	1 h	3 h	12 h
**5a**	38.8	74.7	100.0
**5b**	36.5	72.5	100.0
**5c**	80.1	99.4	-
**5d**	38.0	68.9	100.0
**5e**	77.5	99.7	-
**5f**	74.6	99.4	-
**5g**	36.8	64.2	98.5
**5h**	15.3	37.4	85.4
**7a**	95.3	-	-
**7b**	99.6	-	-
**7c**	99.2	-	-
**7d**	97.6	-	-
**7e**	99.6	-	-
**7f**	98.7	-	-

### 2.5. Tumor Growth Inhibitory Activity of **5e** in a Human Colon Xenograft Model in Vivo

The antitumor activity of **5e**, compared with CPT-11 and SN-38, was investigated in human colon adenocarcinoma SW1116 xenografts in nude mice. The model animals were divided into four experimental groups, eight animals per group. Tested compouds were each administered by injection in the tumor section once per day for contiguous 6 days. The injection doses of test compound **5e**, CPT-11, and SN-38 were 60 mg/kg, respectively. Using the vehicle group as the control, we tested the inhibitory activities of these compounds for 6 days and measured the changes in body weight and tumor size every 2 days. The results showed that 5e inhibited tumor growth by 51% on day 6 and had about the same *in vivo* activity compared to CPT-11 (Table 5). SN-38 was less active than **5e** and CPT-11, showing an inhibition rate of only 8.6%. No toxic death occurred after drug treatment, and the body weight losses induced by **5e**, CPT-11, and SN-38 at the given dose were similar.

**Table 5:** Tumor growth inhibitory activity of **5e** in a human colon xenograft model *in vivo* (x ± SD).

Compound	Dose	Tumor Weight	Inhibitory Rate	Body Weight Change
	(mg/kg)	(g/10g)	(%)	(g)
Vehicle		0.35 ± 0.12		+0.95
**5e**	60	0.17 ± 0.05 *	51	−2.72
Irinotecan	60	0.17 ± 0.04 *	51	−3.10
SN-38	60	0.32 ± 0.12	8.6	−1.45

*: *p* < 0.05, compared with vehicle.

### 2.6. Discussion

In order to overcome the drawbacks of the pharmacokinetic properties of irinotecan and find new water soluble camptothecin prodrugs with improved activity and lower side effects, novel prodrugs of SN-38 were designed and synthesized using amino acids or dipeptides as prodrug carriers to replace the 4-piperidinopiperidine moiety of irinotecan. Because of the poor enzymolysis stability of ester bonds directly formed by the 10-OH moiety f SN-38 when reacting with α-COOH of amino acids, carbamate linkages were used to conjugate the 10-OH of SN-38 and the α-NH_2_ of amino acids or dipeptides. Firstly, the water solubility of the designed prodrugs was improved significantly by converting the free carboxyl groups to their carboxylic salts. Next, we tested the chemical stability and plasma stability of these new compounds in different pH conditions and in human plasma, respectively. The results suggested that the new prodrugs could be stored in acidic solution or in solid state and converted to the active SN-38 in physiological conditions or in human plasma easily, indicating the improved pharmacokinetics property compared with that of Irinotecan. Both *in vitro* and *in vivo* biologic evaluation results showed the predominant antitumor activity of these new compounds. Meanwhile, the compounds exhibited only weak inhibitory activity against AChE, reducing the acute cholinergic diarrhea associated with Irinotecan. These results suggest that these compounds could be used as leads for the further design and development of novel anticancer camptothecin derivatives.

## 3. Experimental Section

### 3.1. General Information

Commercially available reagents were purchased from Alfa Aesar (Shanghai, China), and used as supplied without further purification. Reactions were monitored by thin-layer chromatography performed on silica gel GF_254_ pre-coated plate. Visualization was realized by UV light (365 nm). Purification by flash chromatography was realized on silica gel 200–300 mesh. Proton nuclear magnetic resonance (^1^H-NMR) spectra were recorded in DMSO-*d*_6_ at 400 MHz on a JNM-ECA-400 MHz instrument (JEOL Ltd., Tokyo, Japan). Low Resolution Mass Spectroscopy (AB Sciex Pte. Ltd., Framingham, MA, USA) was recorded on an API 150EX LC/MS system, with an electron ionization spray (ESI) technique.

### 3.2. Synthesis

*10-OCO-(Gly-OBzl)-SN-38* (**4a**). H-Gly-OBzl•HCl (1.23 g, 6.12 mmol) was dissolved in a solution of DCM (60 mL) and NaHCO_3_ (saturated aqueous solution 60 mL), and cooled down to 0 °C. After 10 min, BTC (668 mg, 2.25 mmol) was added and the mixture was stirred for 20 min. The mixture was extracted twice with DCM solution. The combined organic extract were dried over anhydrous sodium sulfate, filtered, and evaporated to give the product as colorless oil without further purification. SN-38 (600 mg, 1.53 mmol) was dissolved in THF, and Et_3_N (618 mg, 6.12 mmol) was added. The mixture was set under N_2_ atmosphere and stirred at room temperature for 30 min. Thereafter, the isocyanate mentioned above was added and the reaction mixture was stirred at 40 °C overnight. The mixture was purified by silica gel chromatography with DCM-acetone = 5:1 as eluent to give compound **4a** (736 mg, 82.5% yield). ^1^H-NMR δ_H_ (ppm): 0.87–0.91 (t, *J* = 7.2 Hz, 3H), 1.27–1.31 (t, *J* = 7.6 Hz, 3H), 1.84–1.91 (m, 2H), 3.15–3.20 (q, *J* = 7.6 Hz, 2H), 3.83–3.84 (d, *J* = 6.0 Hz, 2H), 5.30 (s, 2H), 5.43 (s, 2H), 6.55 (s, 1H), 7.31 (s, 1H), 7.61–7.64 (dd, *J* = 9.6 Hz, 2.4 Hz, 1H), 7.95 (d, *J* = 2.4 Hz, 1H), 8.16–8.18 (d, *J* = 9.6 Hz, 1H), 8.28–8.31 (t, *J* = 6.0 Hz, 1H), 12.68 (s, 1H). MS (ESI) *m/z*: 584.4 [M+H]^+^, 606.2 [M+Na]^+^.

*10-OCO-(Gly-OH)-SN-38* (**5a**). Compound **4a** (736 mg, 1.26 mmol) was dissolved in the solution of THF and ethyl alcohol mixed at a ratio of 7:4. Afterwards, 10% Pd/C (294 mg) was added and the stirred overnight under H_2_ atmosphere. The reaction mixture was filtered, the filtrate was evaporated immediately and chromatographed on silica gel (DCM–acetone = 2:1 with 1% TFA) to give compound **5a** as a yellow powder (446 mg, 71.7% yield). ^1^H-NMR δ_H_ (ppm): 0.87–0.91 (t, *J* = 7.2 Hz, 3H), 1.27–1.31 (t, *J* = 7.6 Hz, 3H), 1.84–1.91 (m, 2H), 3.15–3.20 (q, *J* = 7.6 Hz, 2H), 3.83–3.84 (d, *J* = 6.0 Hz, 2H), 5.30 (s, 2H), 5.43 (s, 2H), 6.55 (s, 1H), 7.31 (s, 1H), 7.61–7.64 (dd, *J =* 9.6 Hz, 2.4 Hz, 1H), 7.95 (d, *J* = 2.4 Hz, 1H), 8.16–8.18 (d, *J* = 9.6 Hz, 1H), 8.28–8.31 (t, *J* = 6.0 Hz, 1H), 12.68 (s, 1H). MS(ESI) *m/z*: 494.3 [M+H]^+^, 516.3 [M+Na]^+^.

*10-OCO-(Ala-OH)-SN-38* (**5b**). Compound **5b** was synthesized by a similar procedure as **5a**. H-Ala-OBzl•TosOH (2.26 g, 6.43 mmol) and SN-38 (600 mg, 1.53 mmol) were employed to produced **4b** (yellow powder, 862 mg, 94%), which was then hydrogenated to **5b** with Pd/C, producing a yellow powder (625 mg, 85.4%). δ_H_ (ppm): 0.87–0.90 (t, *J* = 7.2 Hz, 3H), 1.27–1.31 (t, *J* = 7.6 Hz, 3H), 1.38–1.41 (d, *J* = 7.6 Hz, 3H), 1.82–1.91 (m, 2H), 3.15–3.20 (q, *J* = 7.6 Hz, 2H), 4.12–4.15 (m, 1H), 5.31 (s, 2H), 5.44 (s, 2H), 6.55 (s, 1H), 7.31 (s, 1H), 7.58–7.63 (dd, *J* = 9.2 Hz, 2.4 Hz, 1H), 7.94–7.95 (d, *J* = 2.4 Hz, 1 H), 8.14–8.18 (d, *J* = 9.2 Hz, 1 H), 8.35–8.37 (t, *J* = 7.6 Hz, 1H), 12.79 (s, 1H). MS (ESI) *m/z*: 508.4 [M+H]^+^.

*10-OCO-(Val-OH)-SN-38* (**5c**). Compound **5c** was synthesized by a similar procedure as **5a**. H-Val-OBzl•TosOH (1.94 g, 5.12 mmol) and SN-38 (500 mg, 1.28 mmol) were employed to produce **4c** as a yellow powder (797 mg, 100%), then hydrogenated to **5c** with Pd/C, producing a yellow powder (271 mg, 39.7%). ^1^H-NMR δ_H_ (ppm): 0.87–0.90 (t, *J =* 7.2 Hz, 3 H), 0.98–1.01 (q, *J =* 3.2 Hz, 6 H), 1.28–1.32 (t, *J =* 7.6 Hz, 3H), 1.82–1.93 (m, 2H), 2.14–2.19 (m, 1H), 3.16–3.22 (q, *J =* 7.6 Hz, 2H), 3.96–4.00 (dd, *J =* 5.6 Hz, 8.4 Hz, 1H), 5.32 (s, 2H), 5.43 (s, 2H), 6.51 (s, 1H), 7.32 (s, 1H), 7.61–7.63 (dd, *J =* 2.4 Hz, 9.2 Hz, 1H), 7.95 (d, *J =* 2.4 Hz, 1H), 8.17–8.19 (d, *J =* 9.2 Hz, 1H), 8.22–8.44 (d, *J =* 8.4 Hz, 1H), 12.72 (s, 1H). MS (ESI) *m/z*: 536.6 [M+H]^+^.

*10-OCO-(Glu-OH)-SN-38* (**5d**). Compound **5d** was synthesized by a similar procedure as **5a**. H-Glu(OBzl)-OBzl•TosOH (2.55 g, 5.10 mmol) and SN-38 (500 mg, 1.28 mmol) were employed to produce **4d** (yellow powder, 1.03 g, 100%), which was then hydrogenated to **5d** with Pd/C, producing a yellow powder (368 mg, 51.0%). ^1^H-NMR δ_H_ (ppm): δ *=* 0.87–0.90 (t, *J =* 7.2 Hz, 3H), 1.28–1.31 (t, *J =* 7.6 Hz, 3H), 1.86–1.91 (m, 2H), 2.09–2.12 (m, 2H), 2.48–2.51 (m, 2H), 3.18–3.20 (q, *J =* 6.8 Hz, 2H), 4.12–4.13 (m, 1H), 5.32 (s, 2H), 5.43 (s, 2H), 6.54 (s, 1H), 7.32 (s, 1H), 7.62–7.64 (d, *J =* 8.4 Hz, 1H), 7.96 (s, 1H), 8.15–8.17 (d, *J =* 8.0 Hz, 1H), 8.32–8.34 (d, *J =* 7.6 Hz, 1H), 12.58 (s, 2H). MS (ESI) *m/z*: 566.4 [M+H]^+^, 588.4 [M+Na]^+^.

*10-OCO-(Leu-OH)-SN-38* (**5e**). Compound **5e** was synthesized by a similar procedure as **5a**. H-Leu-OBzl•TosOH (2.01 g, 5.10 mmol) and SN-38 (500 mg, 1.28 mmol) were employed to produce **4e** (yellow powder, 600 mg, 73.6%), which was then hydrogenated to **5e** with Pd/C, producing a yellow powder (51 mg, 19.9%). ^1^H-NMR δ_H_ (ppm): 0.94–1.02 (m, 9 H), 1.33–1.36 (t, *J =* 7.6 Hz, 3H), 1.63–1.66 (m, 1H), 1.69–1.73 (m, 2H), 1.89–1.96 (m, 2H), 3.23–3.27 (q, *J =* 7.6 Hz, 2H), 4.12–4.14 (m, 1H), 5.38 (s, 2H), 5.50 (s, 2H), 6.61 (s, 1H), 7.37 (s, 1H), 7.65–7.68 (dd, *J =* 9.2 Hz, 2.8 Hz, 1H), 8.00–8.01 (d, *J =* 2.4 Hz, 1H), 8.22–8.25 (d, *J =* 9.2 Hz, 1H), 8.38–8.40 (d, *J =* 8 Hz, 1H), 12.81 (s, 1H). MS (ESI) *m/z*: 550.4 [M+H]^+^, 572.4 [M+Na]^+^.

*10-OCO-(Ile-OH)-SN-38* (**5f**). Compound **5f** was synthesized by a similar procedure as **5a**. H-Ile-OBzl•TosOH (2.01 g, 5.10 mmol) and SN-38 (500 mg, 1.28 mmol) were employed to produce **4f** (yellow powder, 757 mg, 92.8%), which was then hydrogenated to **5f** with Pd/C, producing a yellow powder (40 mg, 15.0%). ^1^H-NMR δ_H_ (ppm): 0.86–0.97 (m, 9 H), 1.27–1.33 (m, 4 H), 1.47–1.51 (m, 1H), 1.83–1.93 (m, 3H), 3.16–3.22 (q, *J =* 7.6 Hz, 2H), 4.00–4.02 (m, 1H), 5.32 (s, 2H), 5.44 (s, 2H), 6.55 (s, 1H), 7.32 (s, 1H), 7.61–7.64 (dd, *J =* 8.8 Hz, 2.4 Hz, 1H), 7.95–7.96 (d, *J =* 2.4 Hz, 1H), 8.17–8.19 (d, *J =* 8.8 Hz, 1H), 8.27–8.29 (d, *J =* 8.4 Hz, 1H), 12.80 (s, 1H). MS (ESI) *m/z*: 550.3 [M+H]^+^, 572.4 [M+Na]^+^.

*10-OCO-(l-Asp-OH)-SN-38* (**5g**). Compound **5g** was synthesized by a similar procedure as **5a**. H-l-Asp(OBzl)-OBzl•TosOH (2.48 g, 5.10 mmol) and SN-38 (500 mg, 1.28 mmol) were employed to produce **4g** (yellow powder, 920 mg, 98.6%), which was then hydrogenated to **5g** with Pd/C, producing a yellow powder (521 mg, 75.0%). ^1^H-NMR δ_H_ (ppm): 0.86–0.90 (t, *J =* 7.2 Hz, 3H), 1.27–1.31 (t, *J =* 7.6 Hz, 3H), 1.81–1.92 (m, 2H), 2.65–2.72 (q, *J =* 7.6 Hz, 16.8 Hz, 1H), 2.80–2.85 (q, *J =* 5.8 Hz, 16.8 Hz, 1H), 3.16–3.22 (q, *J =* 7.6 Hz, 2H), 4.40–4.44 (m, 1H), 5.34 (s, 2H), 5.44 (s, 2H), 6.56 (s, 1H), 7.32 (s, 1H), 7.36–7.40 (m, 10H), 7.61–7.63 (dd, *J =* 9.2 Hz, 2.4 Hz, 1H), 7.96–7.97 (d, *J =* 2.4 Hz, 1H), 8.18–8.20 (d, *J =* 9.2 Hz, 1H), 8.34–8.36 (dd, *J =* 8.4 Hz, 1H), 12.89 (s, 2H). MS (ESI) *m/z*: 552.3 [M+H]^+^, 574.5 [M+Na]^+^.

*10-OCO-(d-Asp-OH)-SN-38* (**5h**). Compound **5h** was synthesized by a similar procedure as **5a**. H-d-Asp(OBzl)-OBzl•TosOH (2.48 g, 5.10 mmol) and SN-38 (500 mg, 1.28 mmol) were employed to produce **4h** (yellow powder, 810 mg, 86.8%), which was then hydrogenated to **5h** with Pd/C, producing a yellow powder (422 mg, 70.0%). ^1^H-NMR δ_H_ (ppm): 0.86–0.89 (t, *J =* 7.2 Hz, 3H), 1.27–1.30 (t, *J =* 7.6 Hz, 3H), 1.84–1.90 (m, 2H), 2.71–2.73 (q, 1H), 2.80–2.82 (q, 1H), 3.16–3.18 (q, *J =* 7.6 Hz, 2H), 4.44–4.47 (m, 1H), 5.31 (s, 2H), 5.43 (s, 2H), 6.54 (s, 1H), 7.31 (s, 1H), 7.59–7.62 (dd, *J =* 9.2 Hz, 2.4 Hz, 1H), 7.94–7.95 (d, *J =* 2.4 Hz, 1H), 8.16–8.18 (d, *J =* 9.2 Hz, 1H), 8.34–8.36 (d, *J =* 8.4 Hz, 1H), 12.80–12.83 (s, 2H). MS (ESI) *m/z*: 552.3 [M+H]^+^, 574.5 [M+Na]^+^.

*10-OCO-(d-Asp-Gly-OBzl)-SN-38* (**6a**). Under the ice-bath conditions, **5a** (446 mg, 0.90 mmol) was dissolved in THF (100 mL), followed by addition of HOBt (224 mg, 1.80 mmol) and DIEA (582 mg, 4.50 mmol) in sequence. The mixture was placed under a N_2_ atmosphere and stirred for 20 min, and then H-d-Asp (OBzl)-OBzl•TosOH (485.6 mg, 0.99 mmol), and DCC (206 mg, 0.99 mmol) were added. Thereafter, the reaction mixture was stirred at room temperature for 24 h. The mixture was purified by silica gel chromatography with DCM–acetone = 3:1 as eluent to give compound **6a** ( 241 mg, 34%). ^1^H-NMR δ_H_ (ppm): 0.87–0.90 (t, *J =* 7.2 Hz, 3H), 1.28–1.32 (t, *J =* 7.6 Hz, 3H), 1.86–1.89 (m, 2H), 2.88–2.89 (q, 1H), 2.95–2.96 (q, 1H), 3.14–3.15 (q, *J =* 7.6 Hz, 2H), 3.84–3.89 (d, *J =* 5.6 Hz, 2H), 4.84–4.86 (q, 1H), 4.55–4.59 (q, 1H), 5.13 (s, 2H), 5.21 (s, 2H), 5.27 (s, 2H), 5.45 (s, 2H), 6.57 (s, 1H), 7.35 (s, 11H), 7.63–7.65(dd, *J =* 8.8 Hz, 2.0 Hz, 1H), 7.94 (d, *J =* 2.0 Hz, 1H), 8.14–8.16 (d, *J =* 8.4 Hz, 1H), 8.25 (s, 1H), 8.66–8.68 (d, *J =* 8.0 Hz, 1H). MS (ESI) *m/z*: 789.7 [M+H]^+^, 811.7 [M+Na]^+^.

*10-OCO-(d-Asp-Gly-OH)-SN-38* (**7a**). Compound **6a** (241 mg, 0.31 mmol) was dissolved in a solution of THF and ethyl alcohol mixed at the ratio of 7:4. Afterwards, 10% Pd/C (96.4 mg) was added and the mixture was stirred overnight under a H_2_ atmosphere. The reaction mixture was filtered, the filtrate was evaporated immediately and was chromatographed on silica gel (DCM–acetone = 2:1 with 1% TFA) to give compound **7a** (95 mg, 51% yield), as a yellow powder. ^1^H-NMR δ_H_ (ppm): 0.87–0.90 (t, *J =* 7.2 Hz, 3H), 1.28–1.32 (t, *J =* 7.6 Hz, 3H), 1.86–1.89 (m, 2H), 2.64–2.66 (q, 1H), 2.70–2.73(q, 1H), 3.18–3.20 (q, *J =* 7.6 Hz, 2H), 3.78–3.80 (d, *J =* 5.6 Hz, 2H), 4.59–4.62(q, 1H), 5.33 (s, 2H), 5.44 (s, 2H), 6.52 (s, 1H), 7.33 (s, 1H), 7.64–7.66 (dd, *J =* 8.8 Hz, 2.0 Hz, 1H), 7.96 (d, *J =* 2.0 Hz, 1H), 8.13 (s, 1H), 8.17–8.19 (d, *J =* 8.8 Hz, 1H), 8.32–8.34 (d, *J =* 8.0 Hz, 1H), 12.64 (s, 2H). MS (ESI) *m/z*: 609.4 [M+H]^+^, 631.4 [M+Na]^+^.

*10-OCO-(l-Asp-Gly-OH)-SN-38* (**7b**). Compound **7b** was synthesized by a similar procedure as **7a**. H-l-Asp(OBzl)-OBzl•TosOH (336 mg, 0.69 mmol) and compound **4a** (311 mg, 0.63 mmol) were employed to produce **6b** (yellow powder, 240 mg, 48.3%), which was then hydrogenated to **7b** with Pd/C, producing a yellow powder (115 mg, 72.7%). ^1^H-NMR δ_H_ (ppm): 0.86–0.90 (t, *J =* 7.2 Hz, 3H), 1.27–1.31 (t, *J =* 7.6 Hz, 3H), 1.81–1.92 (m, 2H), 2.60–2.65 (q, 1H), 2.69–2.73 (q, 1H), 3.18–3.20 (q, *J =* 7.6 Hz, 2H), 3.78–3.79 (d, *J =* 6.0 Hz, 2H), 4.55–4.60 (q, 1H), 5.34 (s, 2H), 5.44 (s, 2H), 6.56 (s, 1H), 7.32 (s, 1H), 7.64–7.67 (dd, *J =* 9.2 Hz, 2.4 Hz, 1H), 7.96–7.97 (d, *J =* 2.4 Hz, 1H), 8.17–8.20 (d, *J =* 9.2 Hz, 1H), 8.21–8.24 (t, *J =* 6.0 Hz, 1H), 8.33–8.35 (d, *J =* 7.6 Hz, 1H), 12.79 (s, 2H). MS (ESI) *m/z*: 609.3 [M+H]^+^, 631.4 [M+Na]^+^.

*10-OCO-(Glu-Gly-OH)-SN-38* (**7c**). Compound **7c** was synthesized by a similar procedure as **7a**. H-l-Glu(OBzl)-OBzl•HCL (382 mg, 1.05 mmol) and compound **4a** (471 mg, 0.96 mmol) were employed to produce **6c** (yellow powder, 440 mg, 57.5%), which was then hydrogenated to **7c** with Pd/C, producing a yellow powder (230 mg, 72.8%). ^1^H-NMR δ_H_ (ppm): 0.86–0.90 (t, *J =* 7.2 Hz, 3H), 1.27–1.31 (t, *J =* 7.6 Hz, 3H), 1.83–2.00 (m, 4H), 2.29–2.31(m, 2H), 3.17–3.19 (q, *J =* 7.6 Hz, 2H), 3.79–3.82 (t, *J =* 5.6 Hz, 2H), 4.29–4.30 (m, 1H), 5.32 (s, 2H), 5.44 (s, 2H), 6.56 (s, 1H), 7.32 (s, 1H), 7.64–7.66 (dd, *J =* 9.2 Hz, 2.4 Hz, 1H), 7.96 (d, *J =* 2.4 Hz, 1H), 8.14–8.19 (m, 2H), 8.31 (d, 1H), 12.55 (s, 2H). MS (ESI) *m/z*: 623.4 [M+H]^+^, 645.4 [M+Na]^+^.

*10-OCO-(d-Asp-Ala-OH)-SN-38* (**7d**). Compound **7d** was synthesized by a similar procedure as **7a**. H-d-Asp(OBzl)-OBzl•TosOH (652 mg, 1.34 mmol) and compound **4b** (619 mg, 1.22 mmol) were employed to produce **6d** (yellow powder, 700 mg, 71.3%), which was then hydrogenated to **7d** with Pd/C, producing a yellow powder (90 mg, 40.0%). ^1^H-NMR δ_H_ (ppm): 0.87–0.91 (t, *J =* 7.2 Hz, 3H), 1.22–1.34 (m, 6H), 1.86 (m, 2H), 3.19–3.26 (m, 2H), 3.59 (m, 2H), 4.19 (m, 1H), 4.52–4.59 (q, 1H), 5.33 (s, 2H), 5.43 (s, 2H), 6.55 (s, 1H), 7.31 (s, 1H), 7.63 (dd, *J =* 9.2 Hz, 2.8 Hz, 1H), 7.95 (d, *J =* 2.8 Hz, 1H), 8.16–8.31 (m, 3H), 12.71 (s, 2H). MS (ESI) *m/z*: 623.4 [M+H]^+^, 645.3 [M+Na]^+^.

*10-OCO-(l-Asp-Ala-OH)-SN-38* (**7e**). Compound **7e** was synthesized by a similar procedure as **7a**. H-l-Asp(OBzl)-OBzl•TosOH (392 mg, 0.77 mmol) and compound **4b** (392 mg, 0.77 mmol) were employed to produce **6e** (yellow powder, 400 mg, 64.6%), which was then hydrogenated to **7e** with Pd/C, producing a yellow powder (124 mg, 42.6%). ^1^H-NMR δ_H_ (ppm): 0.87–0.91 (t, *J =* 7.2 Hz, 3H), 1.29–1.31 (t, *J =* 7.6 Hz, 3H), 1.33–1.35 (d, *J =* 7.6 Hz, 3H), 1.82–1.93 (m, 2H), 2.62–2.67 (q, 1H), 2.70–2.76 (q, 1H), 3.15–3.20 (q, *J =* 7.6 Hz, 2H), 4.19–4.24 (m, 1H), 4.58–4.60 (m, 1H), 5.31 (s, 2H), 5.44 (s, 2H), 6.52 (s, 1H), 7.32 (s, 1H), 7.62–7.65 (dd, *J =* 9.2 Hz, 2.4 Hz, 1H), 7.94–7.95 (d, *J =* 2.4 Hz, 1H), 8.14–8.20 (m, 2H), 8.27–8.29 (d, *J =* 8.0 Hz, 1H), 12.64 (s, 2H). MS (ESI) *m/z*: 623.4 [M+H]^+^, 645.5 [M+Na]^+^.

*10-OCO-(Glu-Ala-OH)-SN-38* (**7f**). Compound **7f** was synthesized by a similar procedure as **7a**. H-l-Glu(OBzl)-OBzl•HCl (493 mg, 1.36 mmol) and compound **4b** (625 mg, 1.23 mmol) were employed to produce **6f** (yellow powder, 690 mg, 69.0%), which was then hydrogenated to **7f** with Pd/C, producing a yellow powder (260 mg, 50.3%). ^1^H-NMR δ_H_ (ppm): 0.87–0.91 (t, *J =* 7.2 Hz, 3H), 1.29–1.31 (t, *J =* 7.6 Hz, 3H), 1.33–1.35 (d, *J =* 7.2 Hz, 3H), 1.82–1.91 (m, 2H), 2.01–2.03 (m, 2H), 2.29–2.34 (m, 2H), 3.15–3.20 (q, *J =* 7.6 Hz, 2H), 4.19–4.22 (t, *J =* 7.2 Hz, 1H), 4.27–4.29 (m, 1H), 5.31 (s, 2H), 5.44 (s, 2H), 6.51 (s, 1H), 7.32 (s, 1H), 7.62–7.65 (dd, *J =* 9.2 Hz, 2.4 Hz, 1H), 7.94 (d, *J =* 2.4 Hz, 1H), 8.14–8.18 (m, 2H), 8.24–8.26 (d, *J =* 8.0 Hz, 1H), 12.42 (s, 2H). MS (ESI) *m/z*: 637.3 [M+H]^+^, 659.4 [M+Na]^+^.

### 3.3. Cytotoxicity Study

The *in vitro* antitumor activity was evaluated on two human cancer cell lines with the MTT method. SGC-7901 is a human gastric adenocarcinoma cancer cell line and HeLa is a human cervical carcinoma cell line. Cells were provided by the Jilin Province Tumor Institute. Cells were maintained in IMDM medium supplemented with 10% fetal calf serum, 2 mM glutamine, 100 U/mL penicillin, and 100 μg/mL streptomycin, at 37 °C in a humidified atmosphere containing 5% CO_2_. The human cancer lines SGC-7901 and HELA at a concentration of 2.5 × 10^4^ μg·mL^−1^ were incubated in a 96-well microtiter plate (100 μL in every well) for 24 h (37 °C, 5%CO_2_). Then the cells were incubated continuously for 72 h following various concentrations of test drugs were added. The concentrations are 50 μM, 10 μM, 2 μM, 0.4 μM, 0.08 μM, 0.016 μM, 0.0032 μM, respectively. After the 100 μL 0.05% MTT solution was added to each well, the plate was incubated for a further 4 h before removal of medium, the media removed. Formazan crystals were dissolved with 100 μL DMSO, then the absorbance was detected at 490 nm.

### 3.4. Acetylcholinesterase Inhibition Assay

The AChE inhibition by all compounds was studied according to a modification of the method of Ellman *et al*. [15]. The activity was assayed with AChE from electric eel in the presence or absence of the test compounds in phosphate buffer (pH7.3). After the incubation at 37 °C for 1 min, 0.38 mM 5,5-dithiobisnitrobenzoic acid and 0.56 mM ATCh were added to the buffer. The changes in absorbance were read at 412 nm, per 15 s intervals for 3 min by a spectrophotometer (UV-2500, Shimadzu Corp., Kyoto, Japan).

### 3.5. Stability Test

The stability of all compounds was investigated in PBS and human plasma by HPLC using a C18 analytical column. The mobile phase was composed of water and acetonitrile at a ratio of 70:30, respectively, containing 1% trifluoroacetic acid. The amount of the compound and its metabolite SN-38 were flowed at the rate of 0.8 mL·min^−1^ and detected by the UV wavelength of 370 nm. Stability test in PBS: each compound was dissolved in PBS (pH 7.4 and pH 4.6), incubated at 37 °C and analyzed at 1, 3, 6, 12 h. Stability test in plasma: The test compounds were dissolved in DMSO and diluted to 1 mM by 0.9% physiological saline. Forty μL of each solution was added into plasma with a final concentration of 0.1 mM. The mixture was incubated in a 37 °C water bath following the vigorous vortex mixing for 15 s. Samples were analyzed at 1, 3, 6, 12 h. 400 μL of cold acetonitrile (containing 1% trifluoroacetic acid) was added to each sample, vortex-mixed for 15 s and centrifuged at 3500 rpm for 15 min at 4 °C.

## 4. Conclusions

In summary, 14 novel SN-38 prodrugs were prepared by conjugating amino acids or dipeptides to the 10-hydroxy group of SN-38 via a carbamate linkage. These prodrugs were found to have much greater antitumor activities than SN-38. The synthesized compounds completely regenerated SN-38 in pH 7.4 buffer or in human plasma, so higher antitumor activity and less interpatient pharmacokinetic variability than with irinotecan treatment are expected. The ability of these prodrugs to inhibit AChE activity was also significantly reduced; thus, the administration of these compounds should cause less severe side effects owing to their diminished inhibition of AChE.

## Supplementary Materials

Supplementary materials can be accessed at: http://www.mdpi.com/1420-3049/19/12/19718/s1.
