# Supplementary material for: Synthesis and Biological Evaluation of Novel 10-Substituted-7-ethyl-10-hydroxycamptothecin (SN-38) Prodrugs
Source: Molecules. 2014 Nov 27;19(12):19718–31. doi: 10.3390/molecules191219718 (PMC6270839; doi:10.3390/molecules191219718)

Supplementary Materials

Figure S1. ^{1}H-NMR spectra of **4a**.

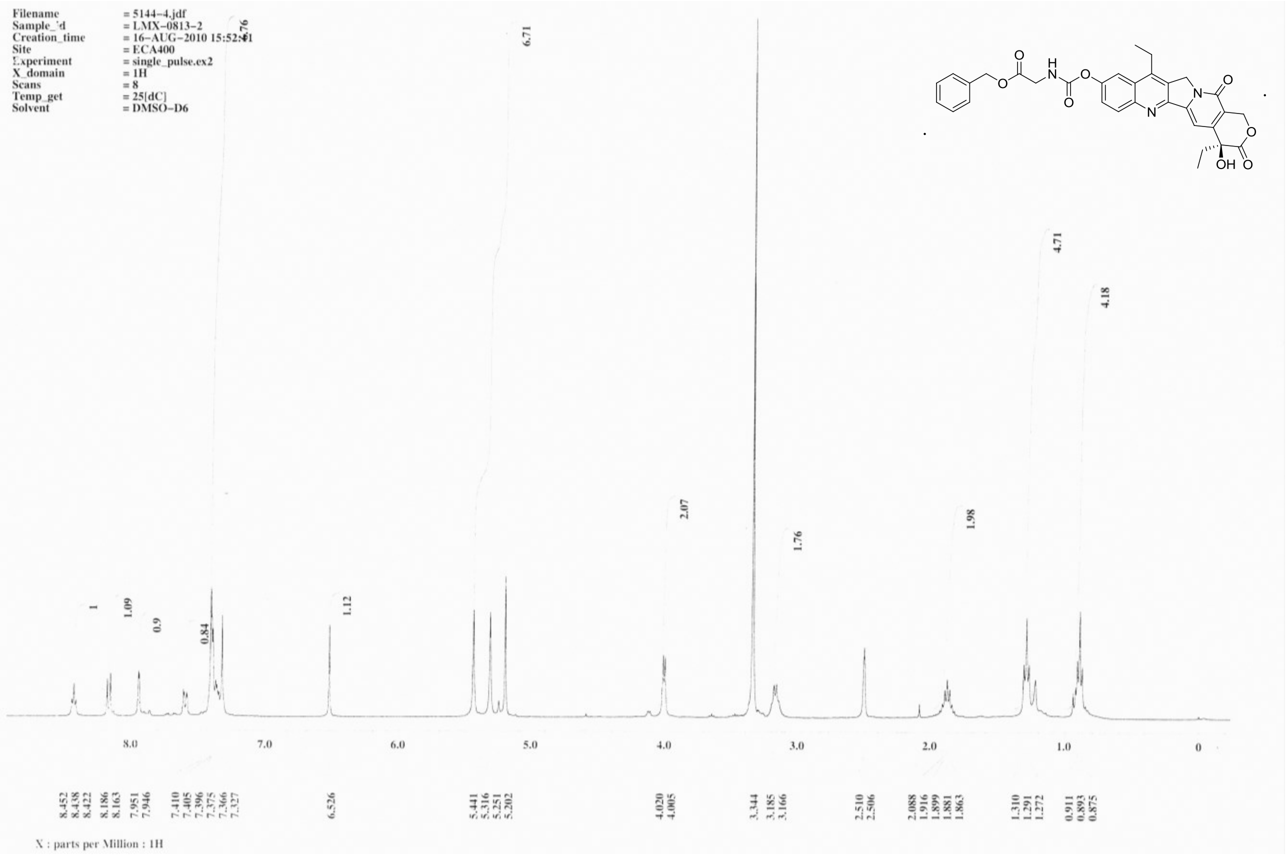

Figure S2. MS spectra of **4a**.

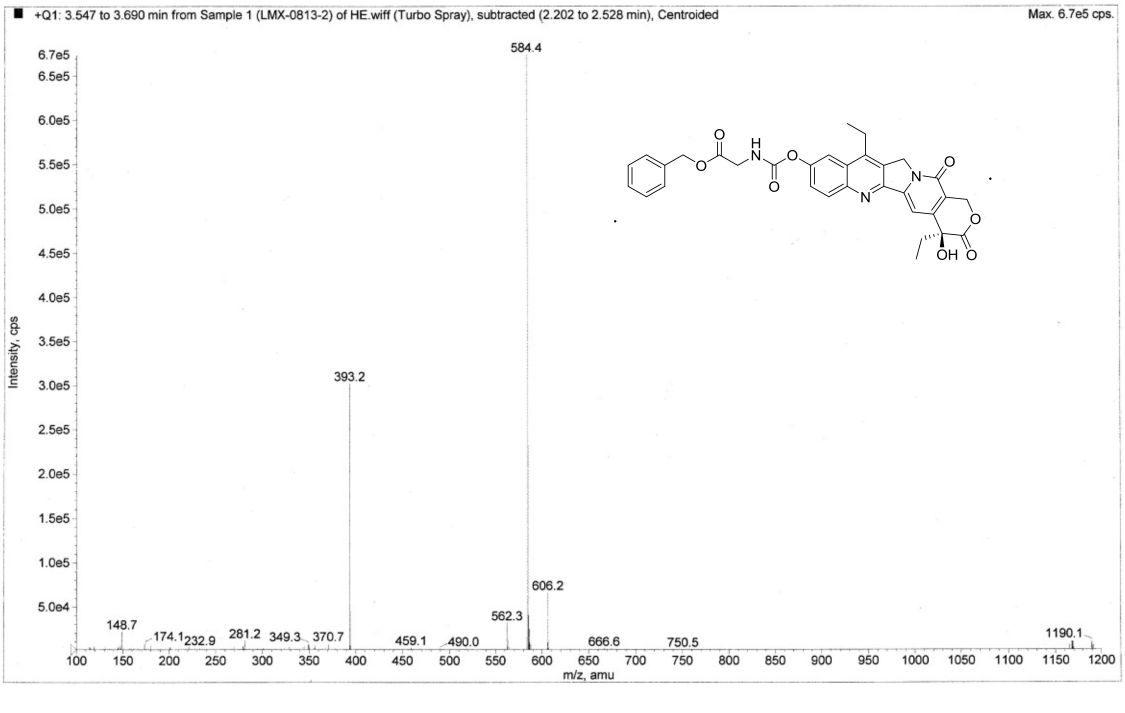

Figure S3. ^{1}H-NMR spectra of **5a**.

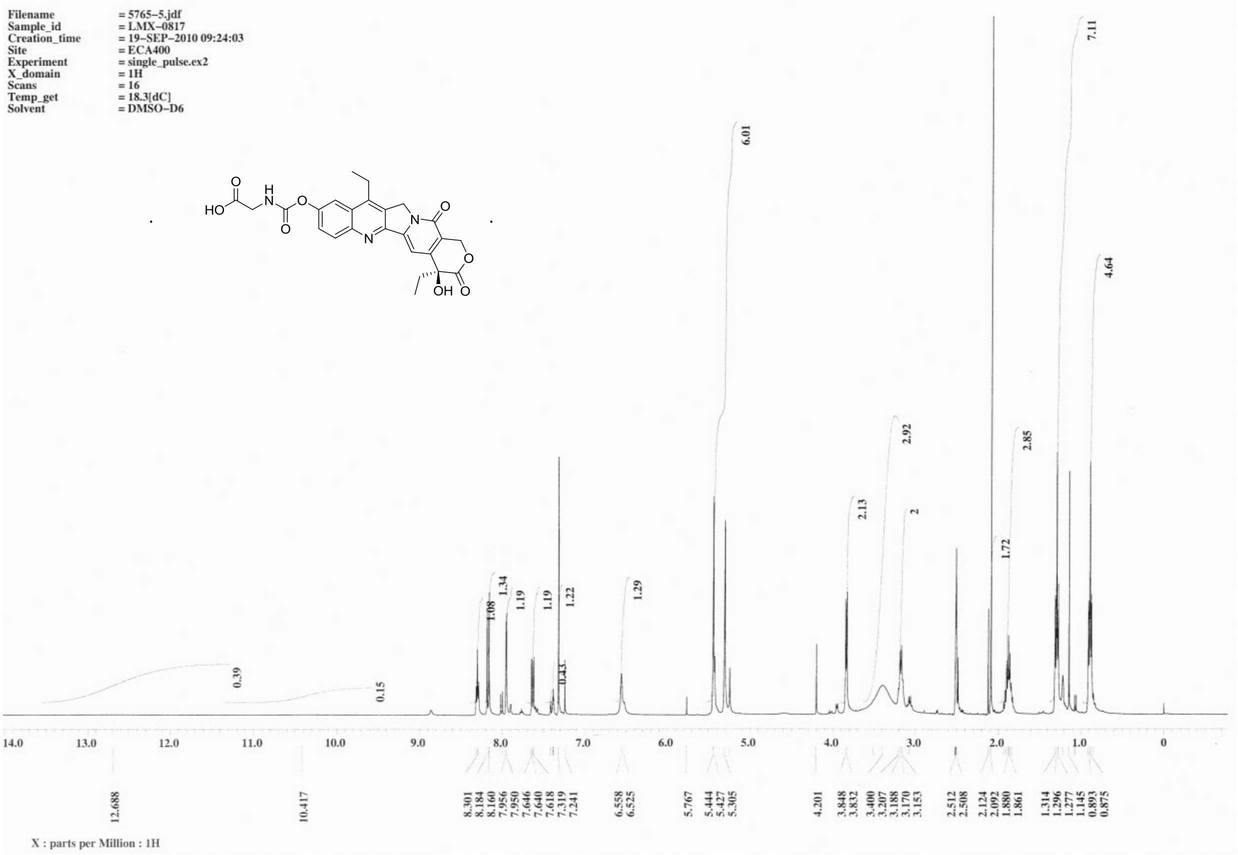

Figure S4. MS spectra of **5a**.

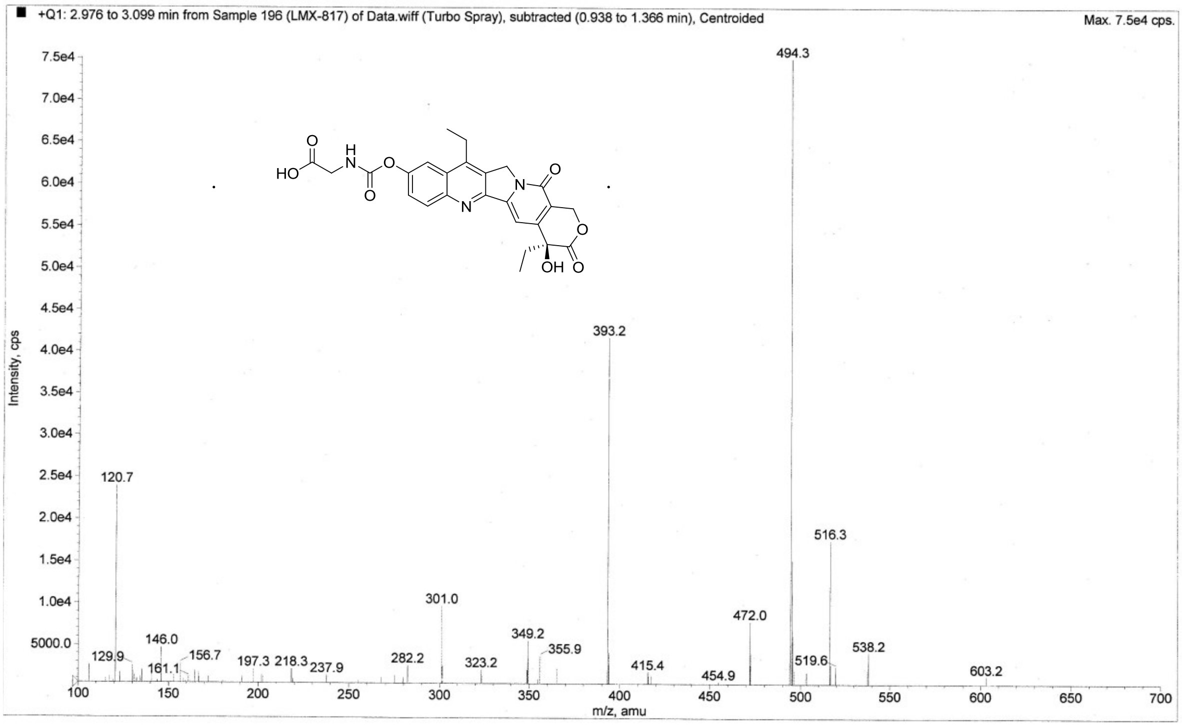

Figure S5. ^{1}H-NMR spectra of **4b**.

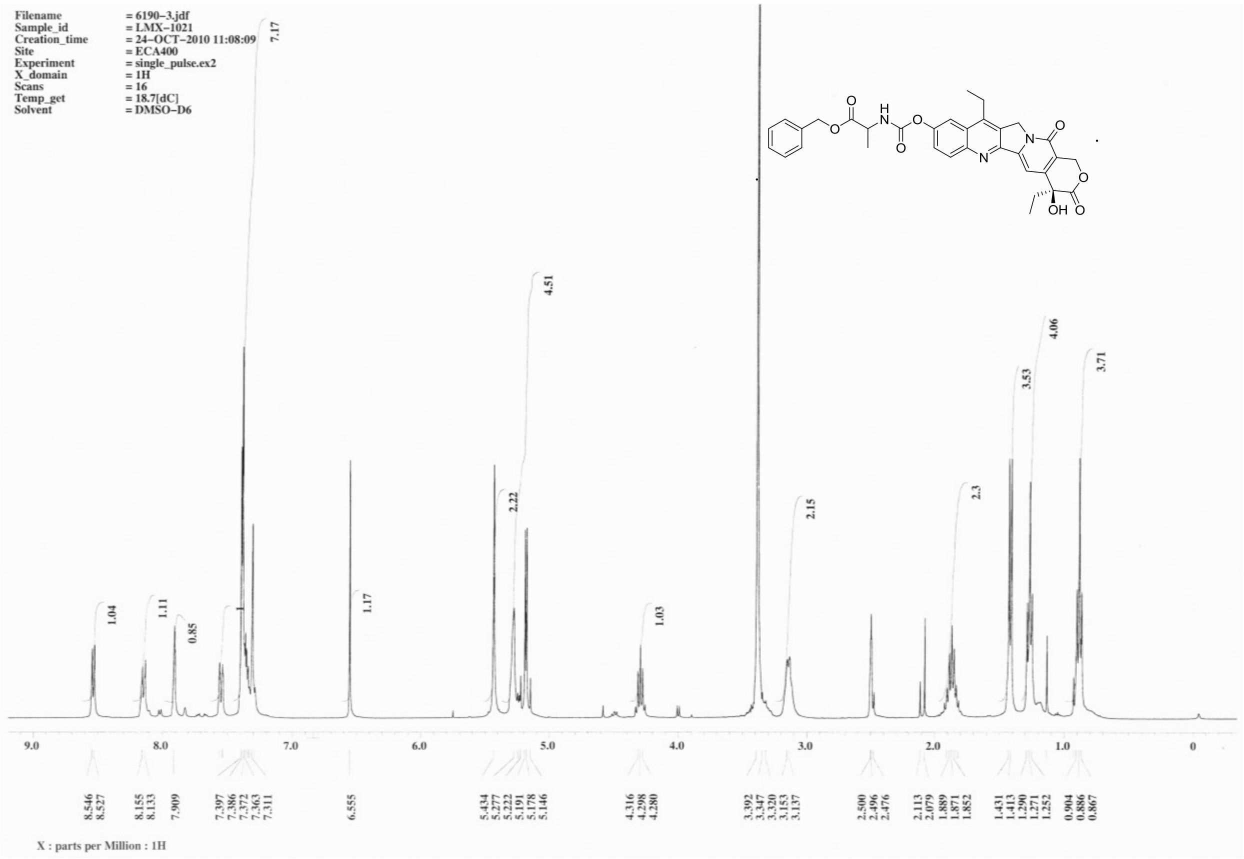

Figure S6. MS spectra of **4b**.

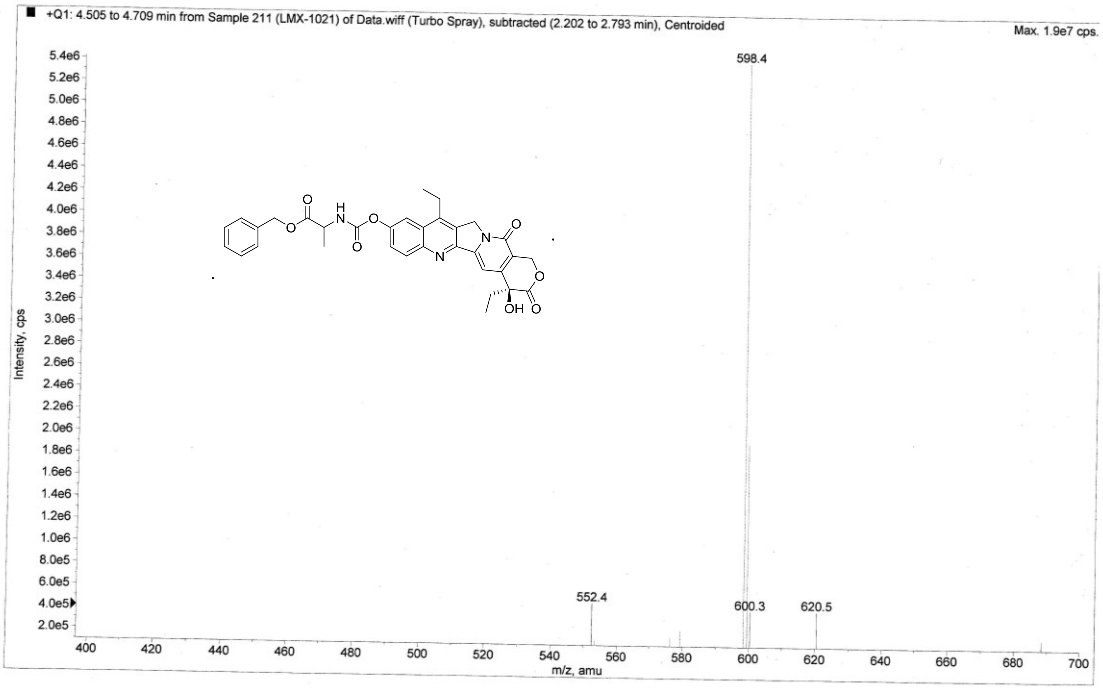

Figure S7. ^{1}H-NMR spectra of **5b**.

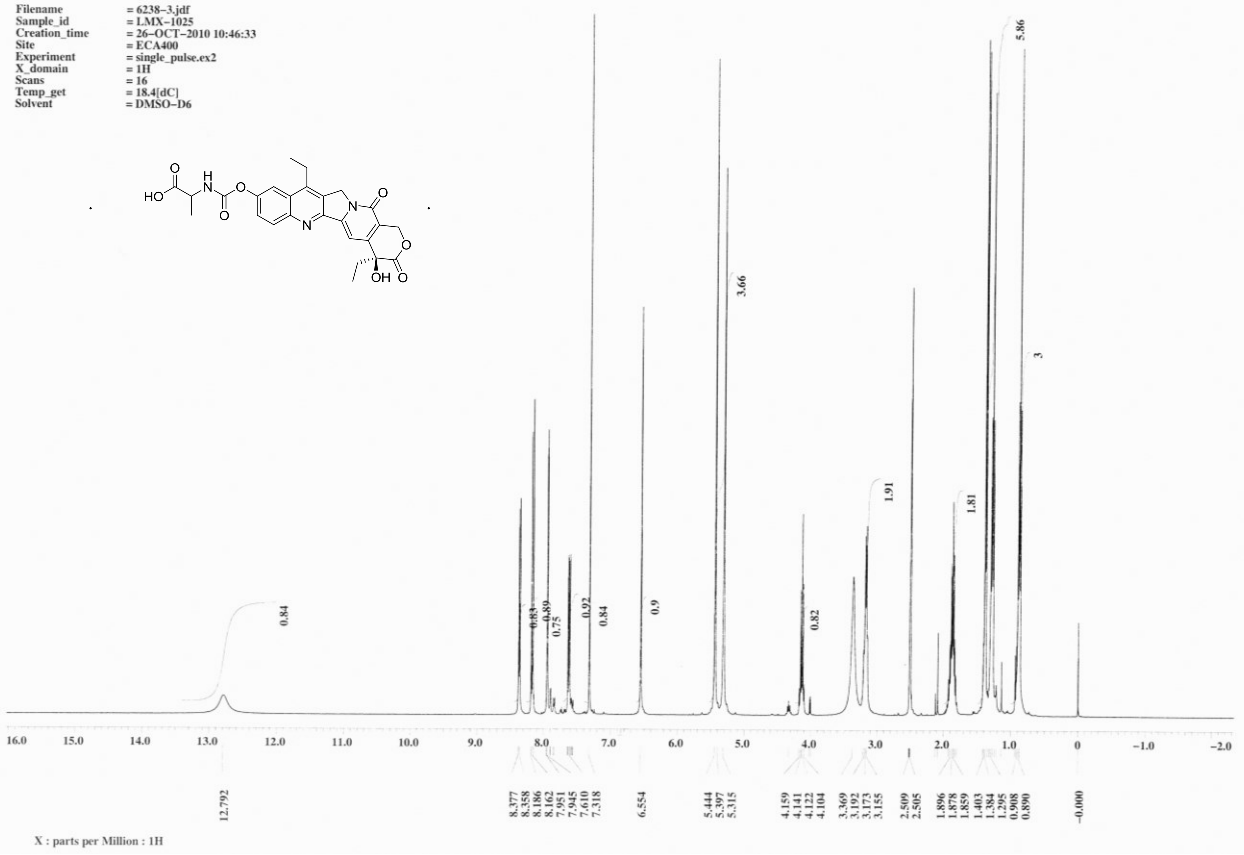

Figure S8. MS spectra of **5b**.

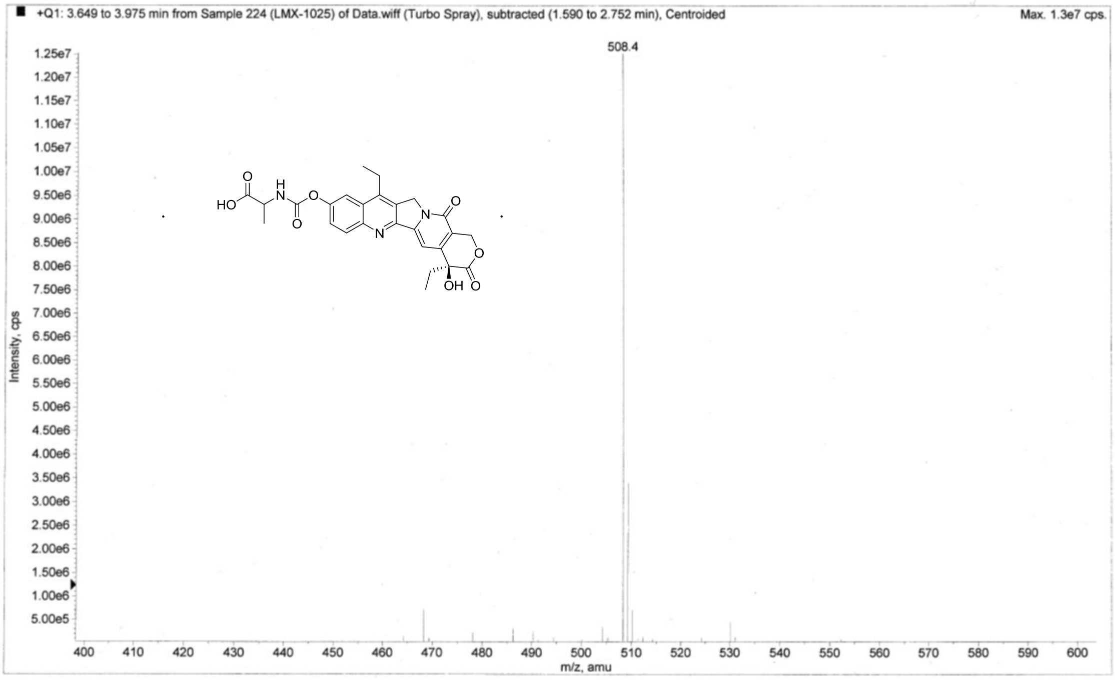

Figure S9. ^{1}H-NMR spectra of **4c**.

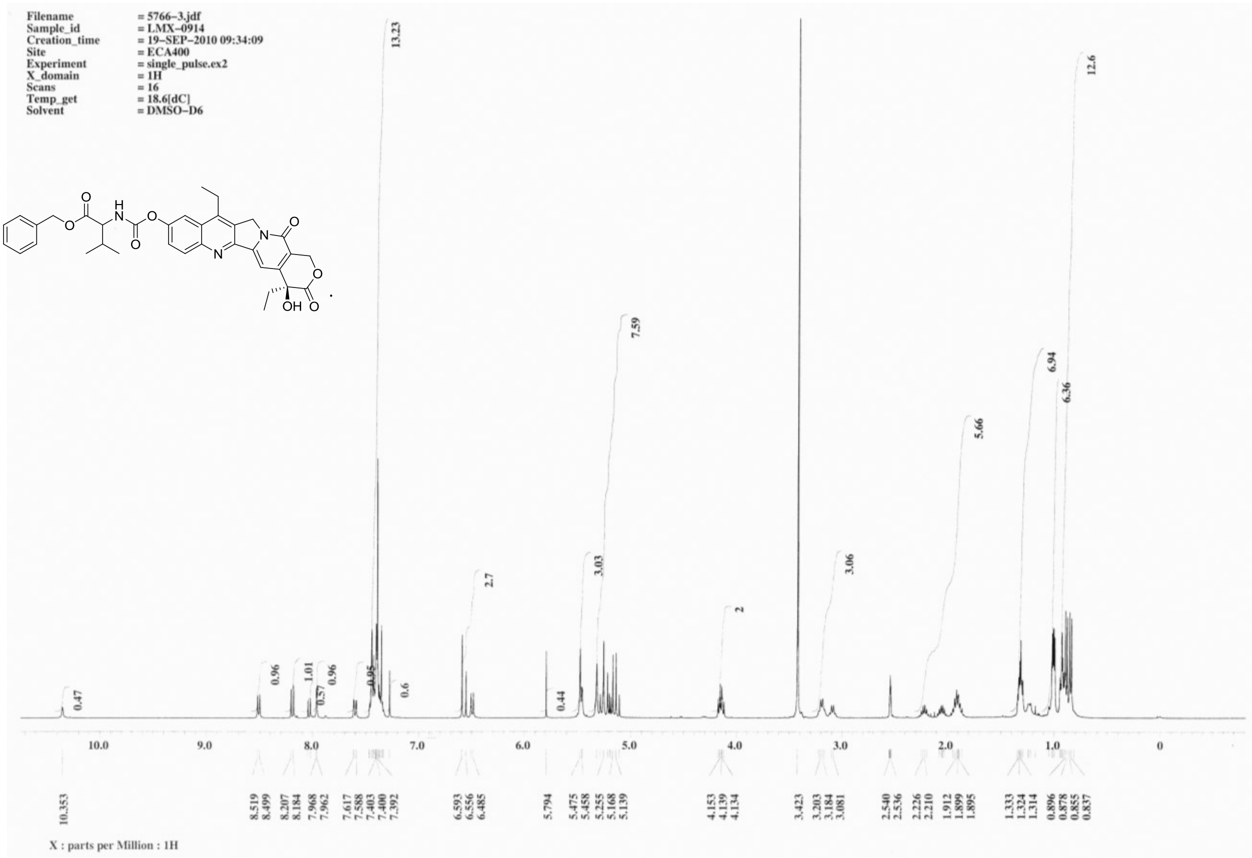

Figure S10. MS spectra of **4c**.

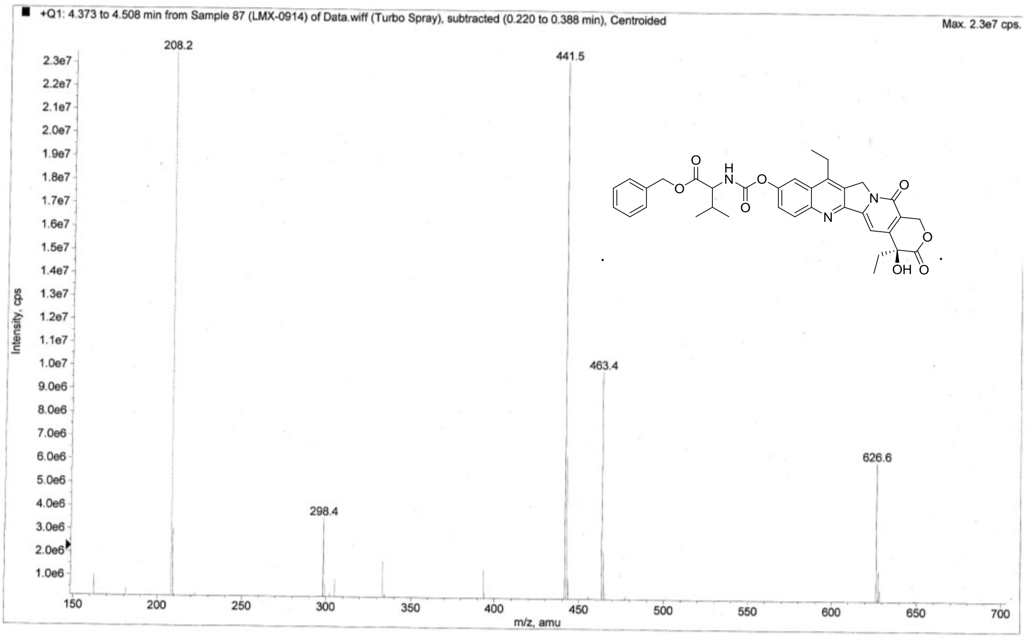

Figure S11. ^{1}H-NMR spectra of **5c**.

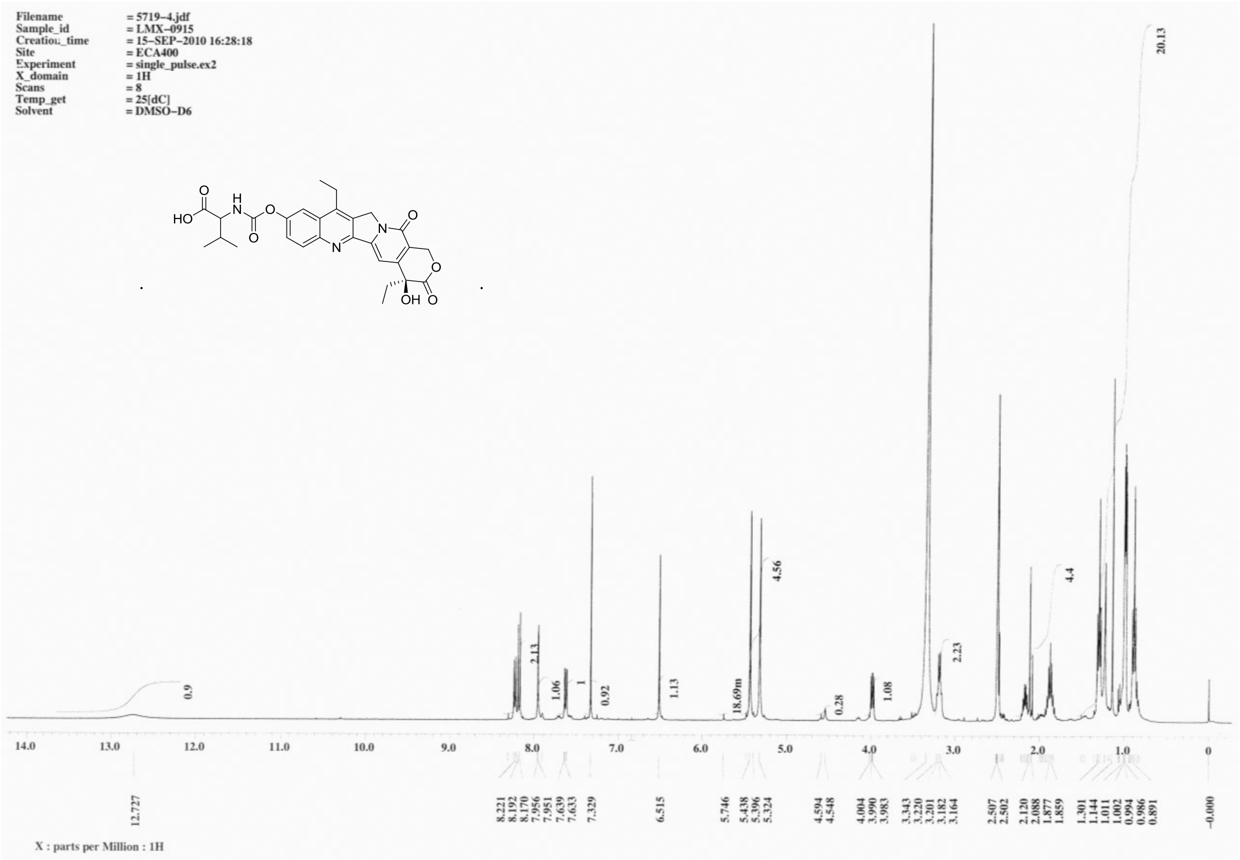

Figure S12. MS spectra of **5c**.

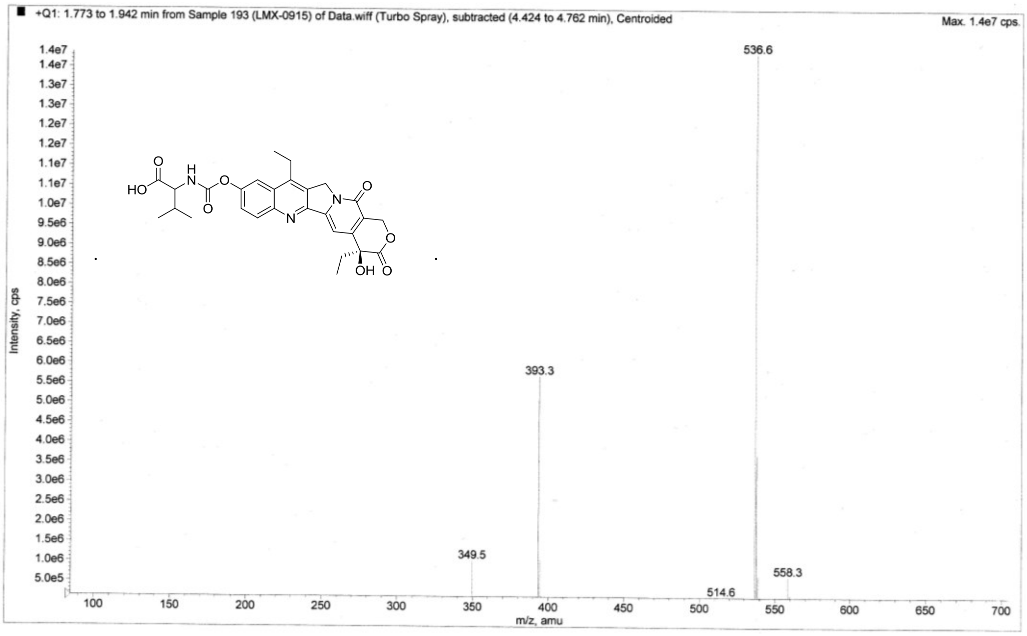

Figure S13. ^{1}H-NMR spectra of **4d**.

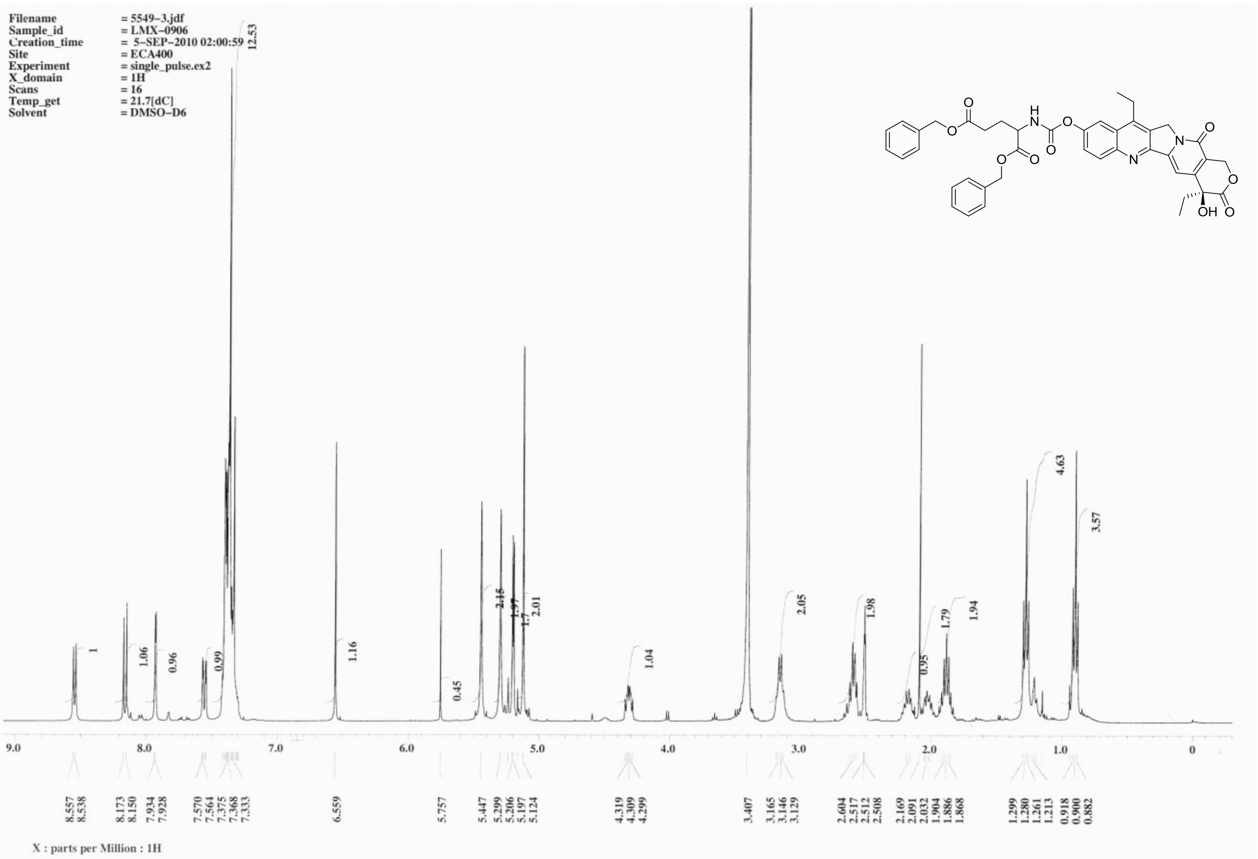

Figure S14. MS spectra of **4d**.

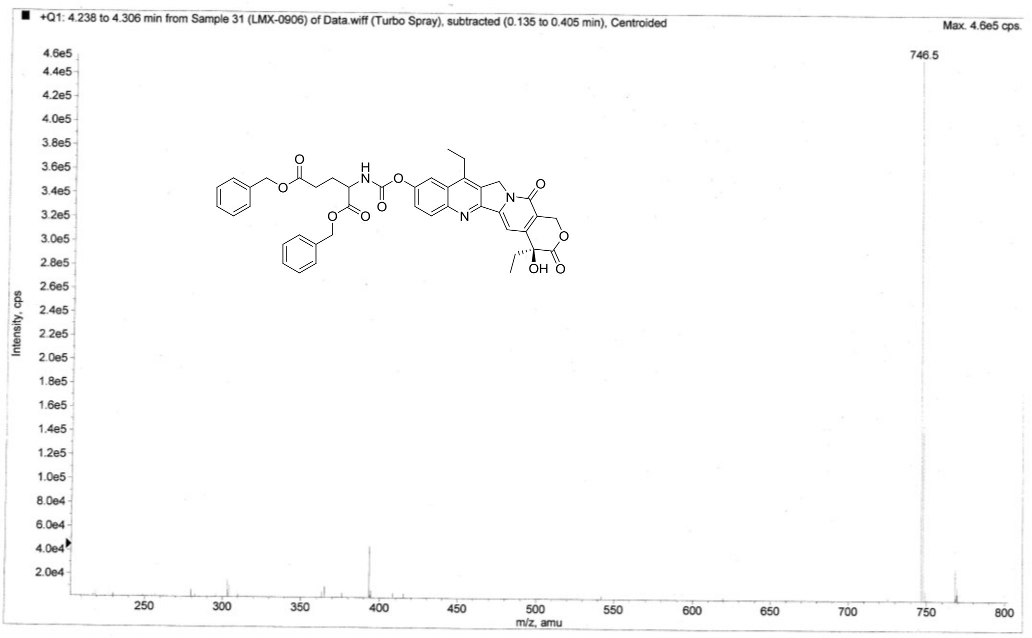

Figure S15. ^{1}H-NMR spectra of **5d**.

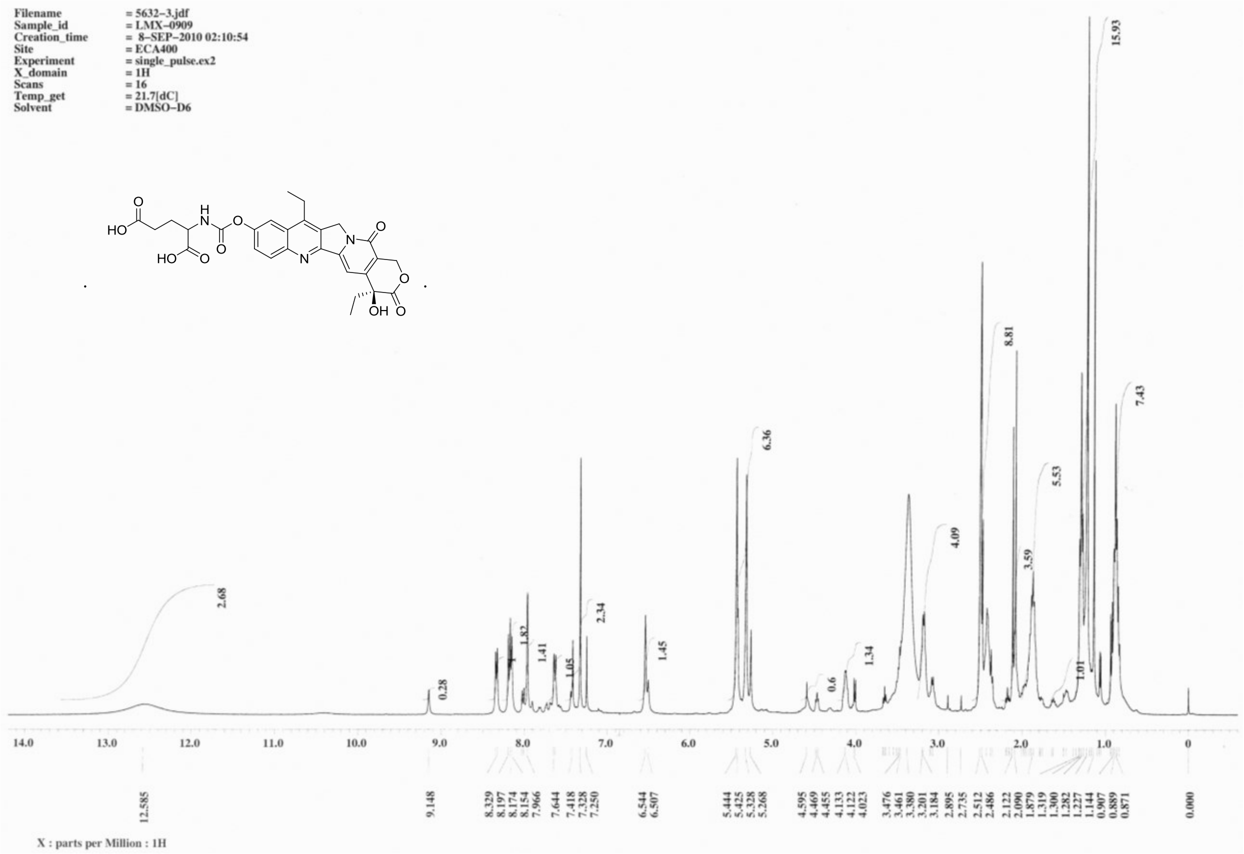

Figure S16. MS spectra of **5d**.

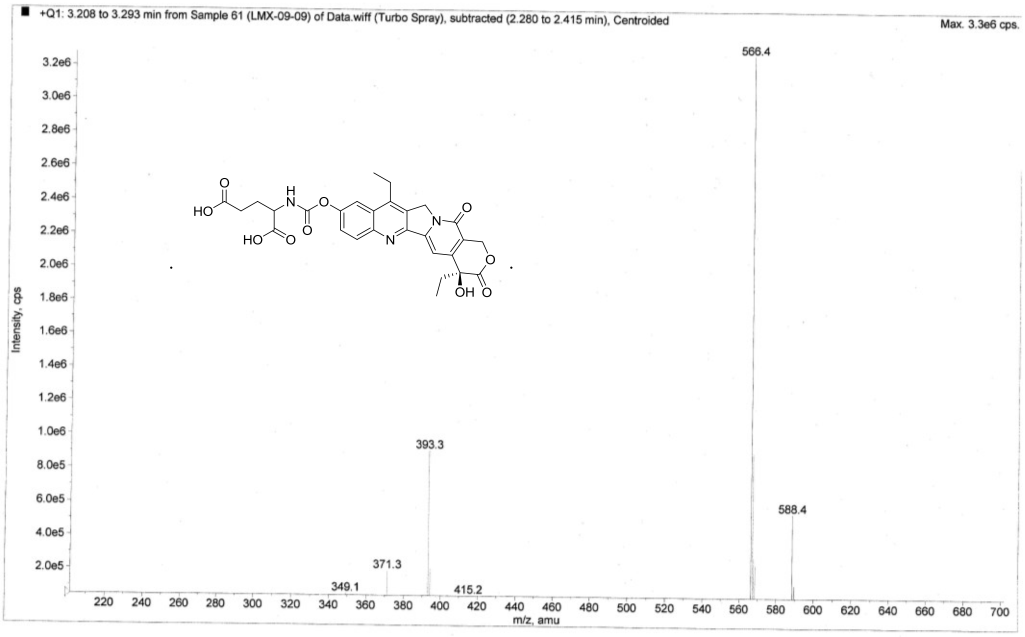

Figure S17. ^{1}H-NMR spectra of **4e**.

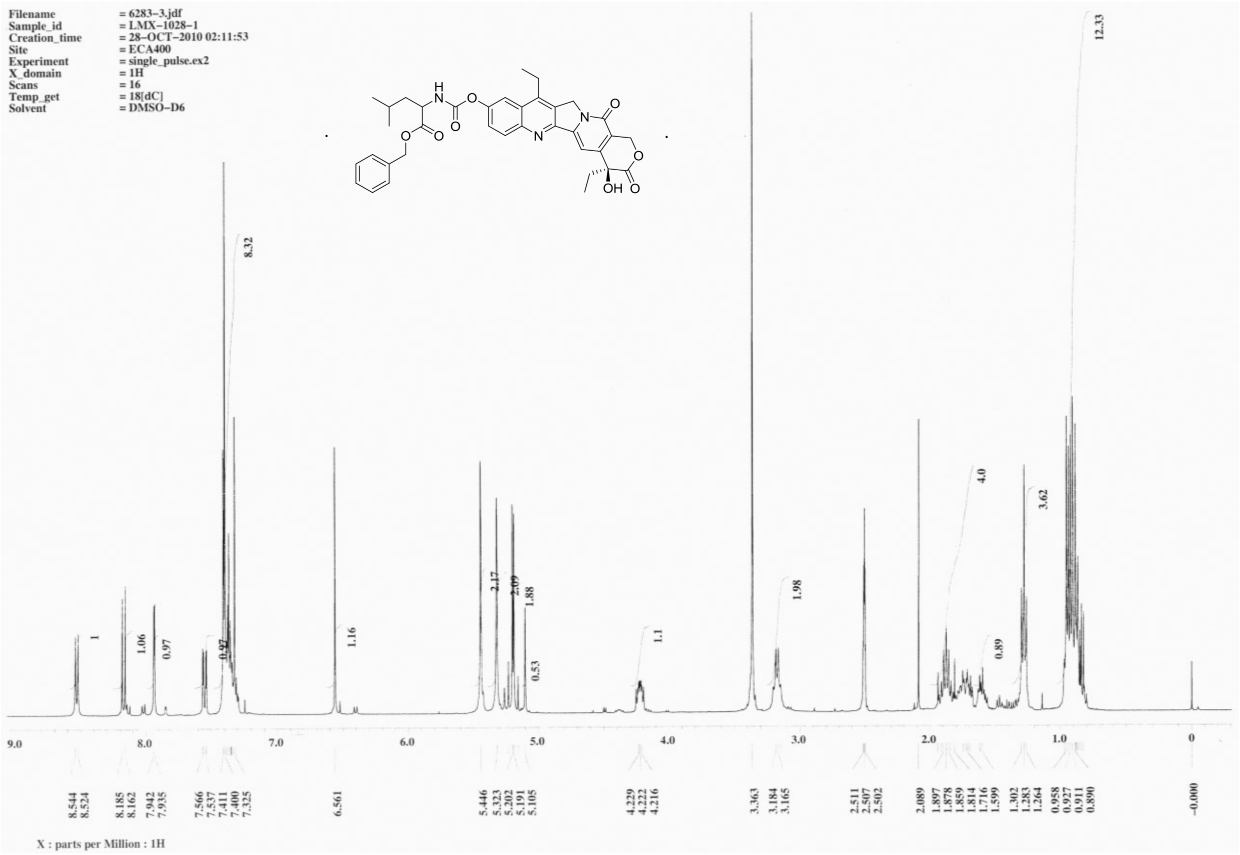

Figure S18. MS spectra of **4e**.

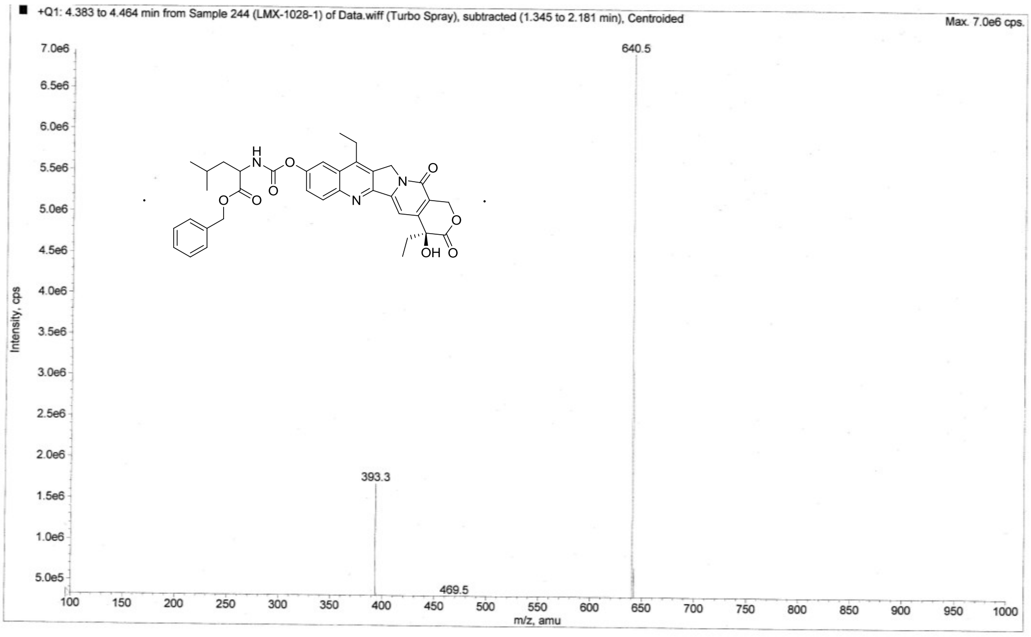

Figure S19. ^{1}H-NMR spectra of **5e**.

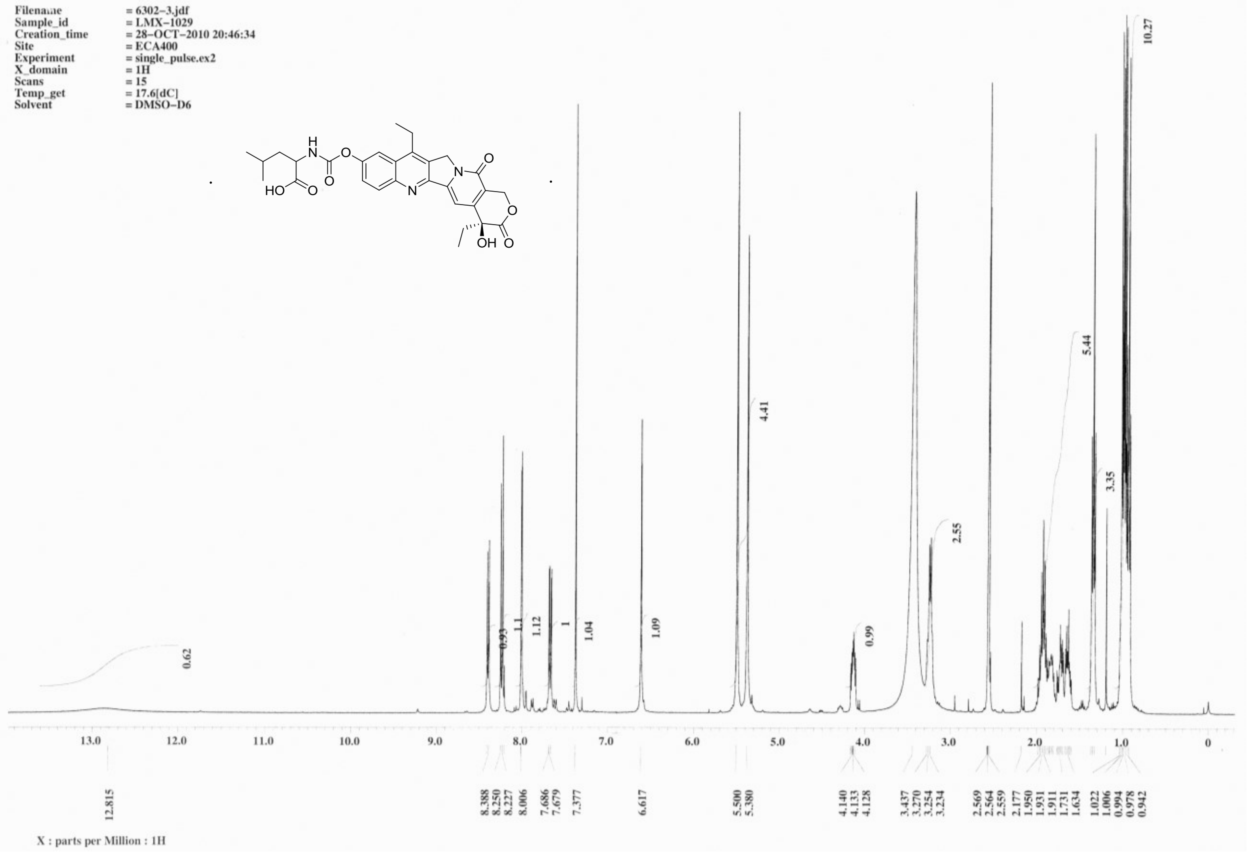

Figure S20. MS spectra of **5e**.

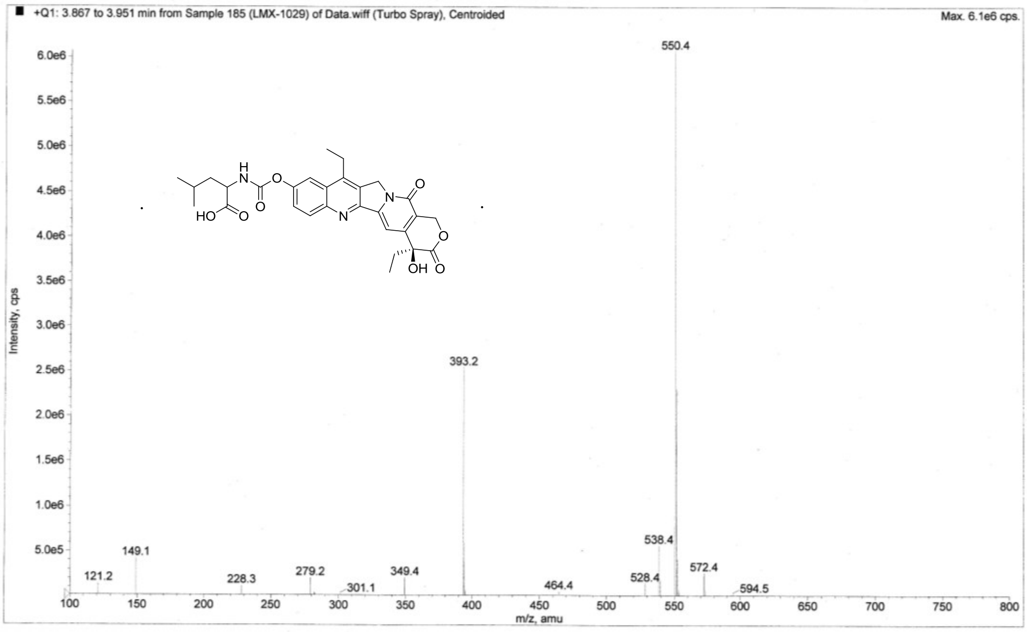

Figure S21. ^{1}H-NMR spectra of **4f**.

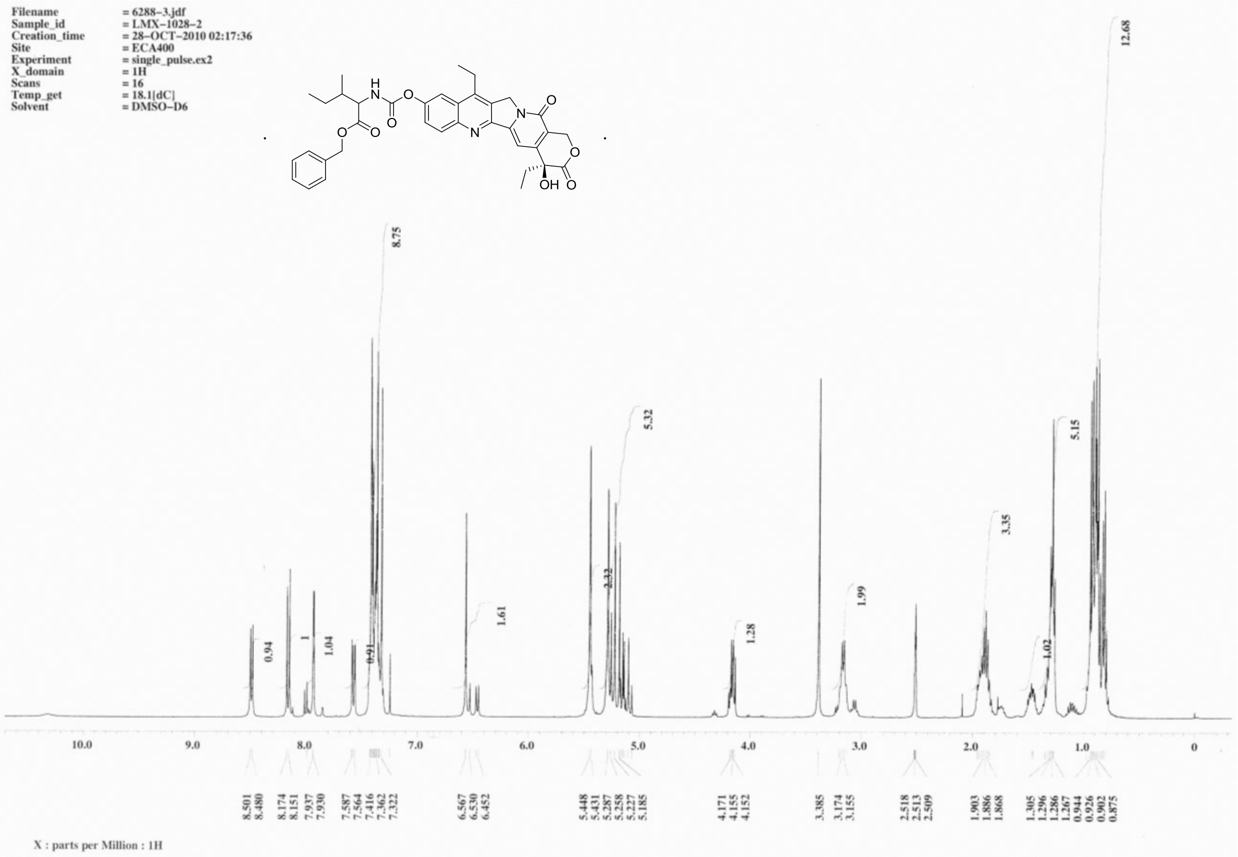

Figure S22. MS spectra of **4f**.

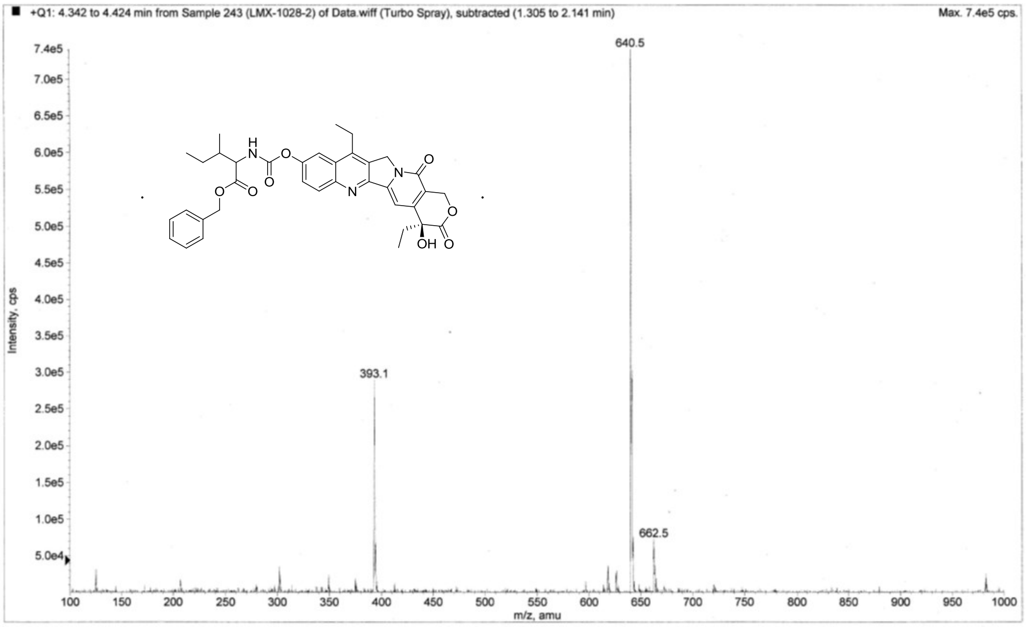

Figure S23. ^{1}H-NMR spectra of **5f**.

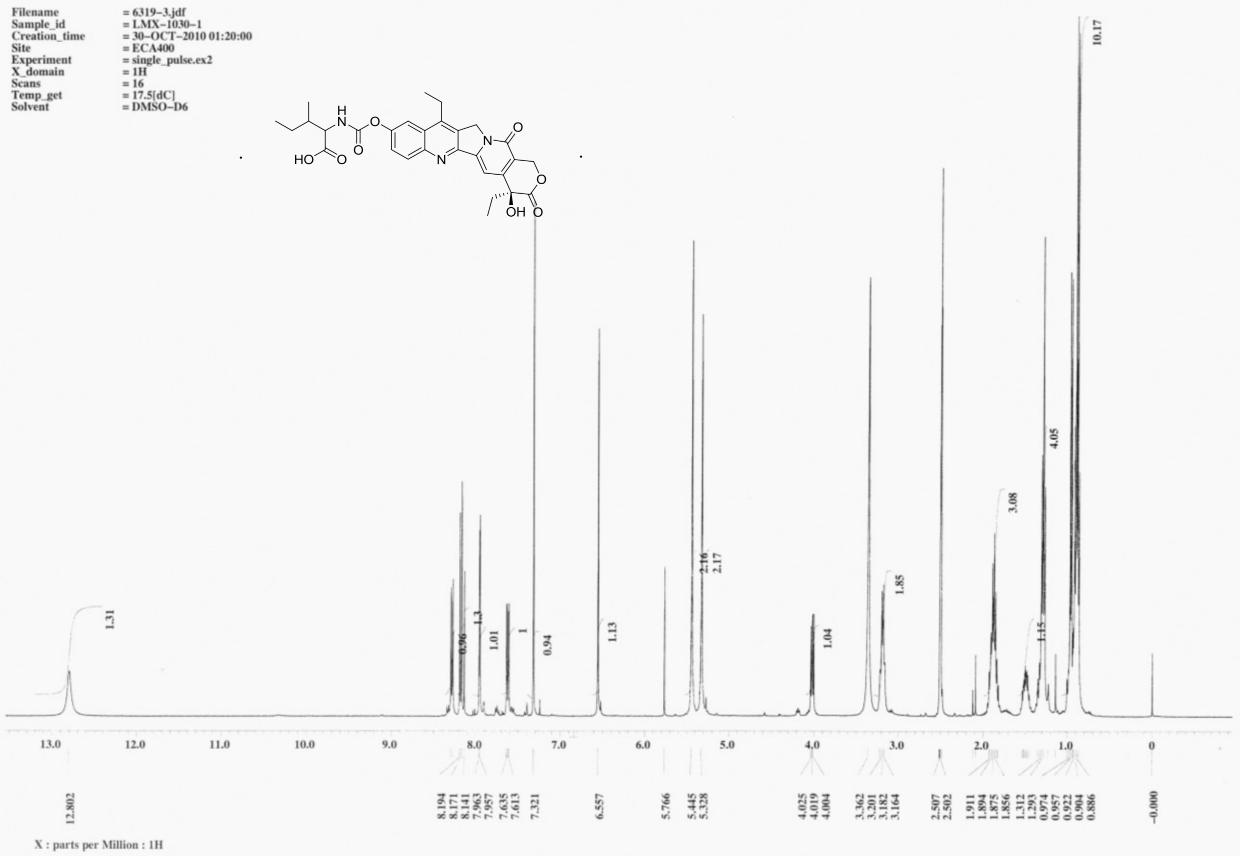

Figure S24. MS spectra of **5f**.

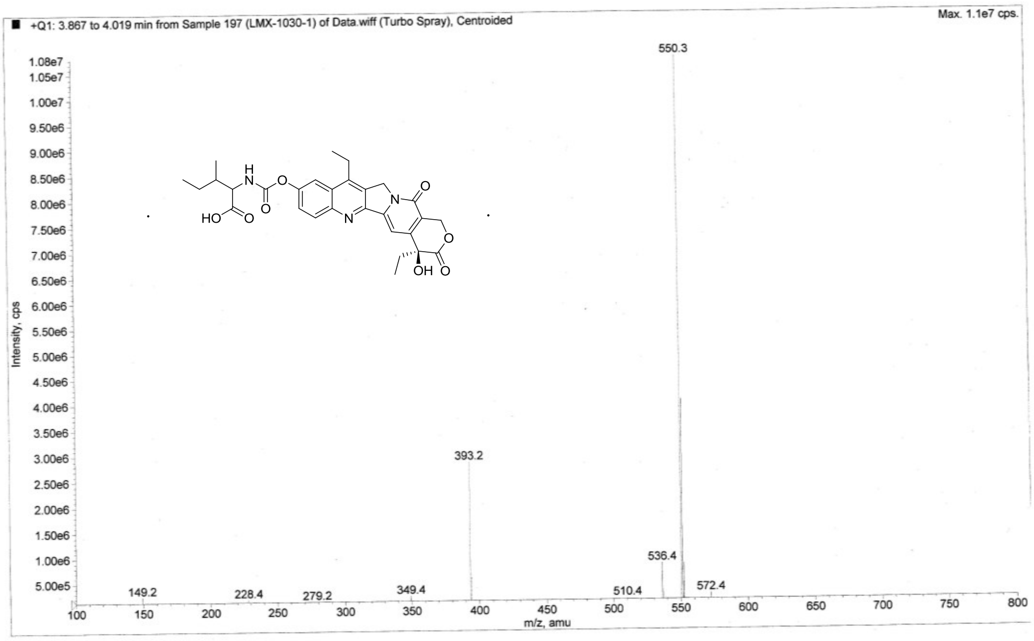

Figure S25. ^{1}H-NMR spectra of **4g**.

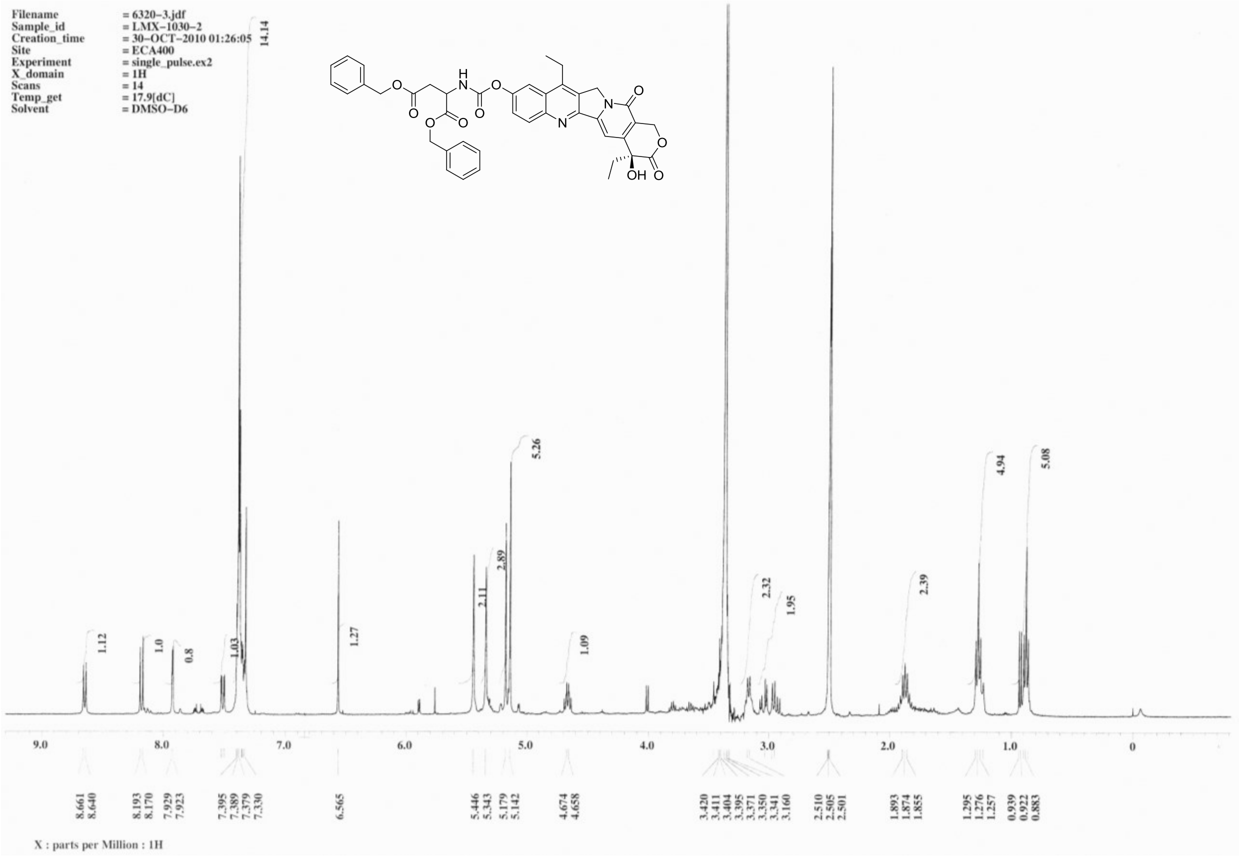

Figure S26. MS spectra of **4g**.

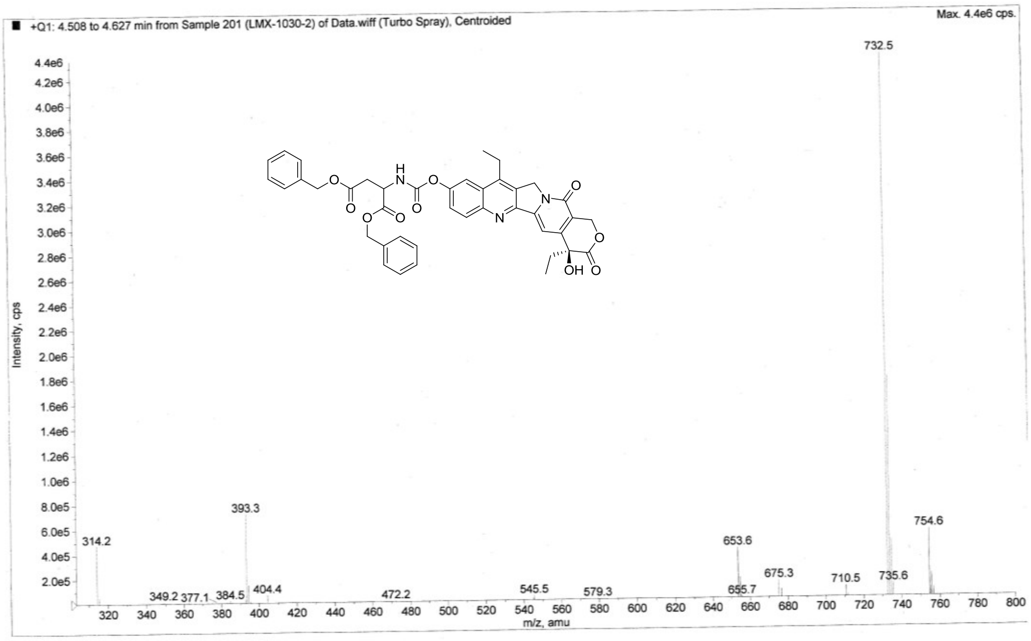

Figure S27. ^{1}H-NMR spectra of **5g**.

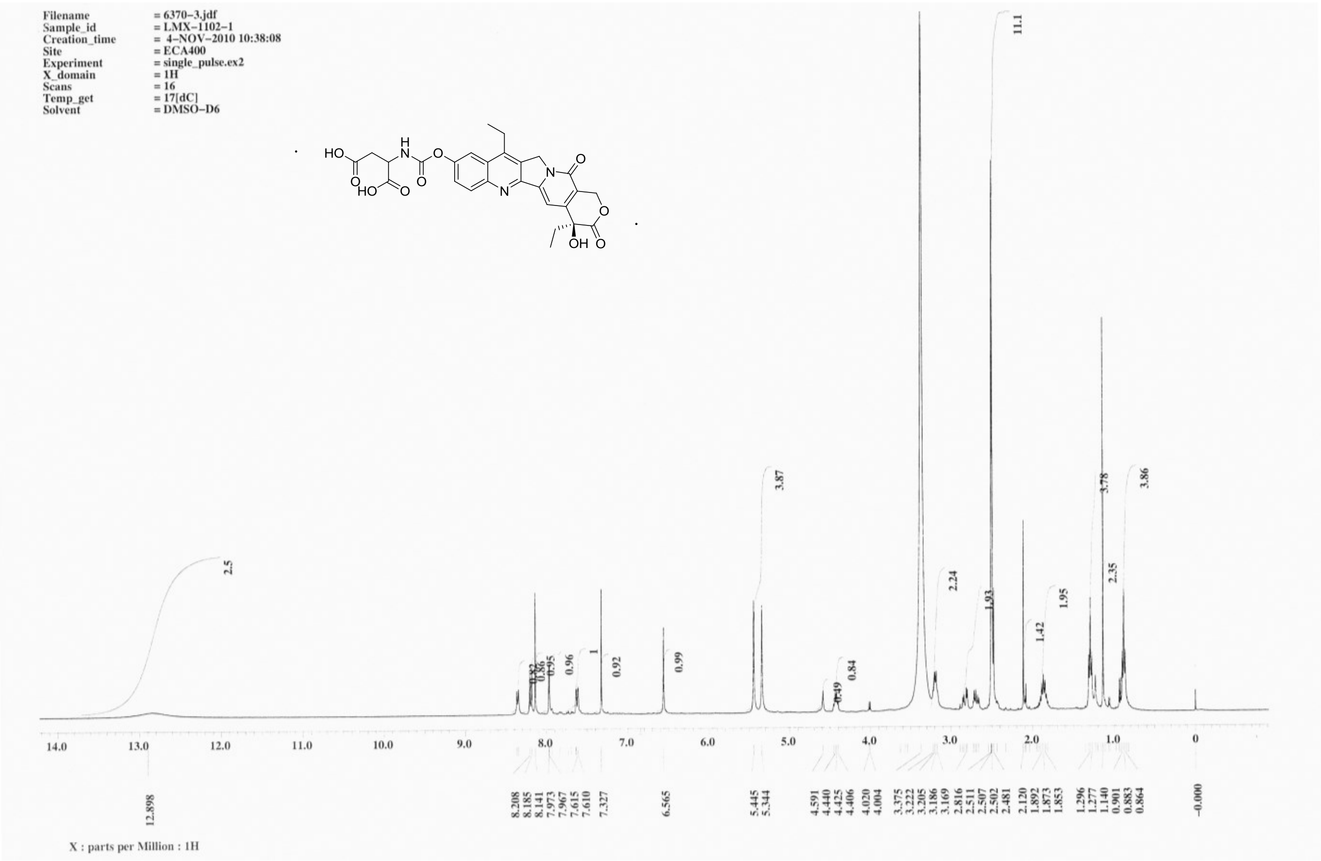

Figure S28. MS spectra of **5g**.

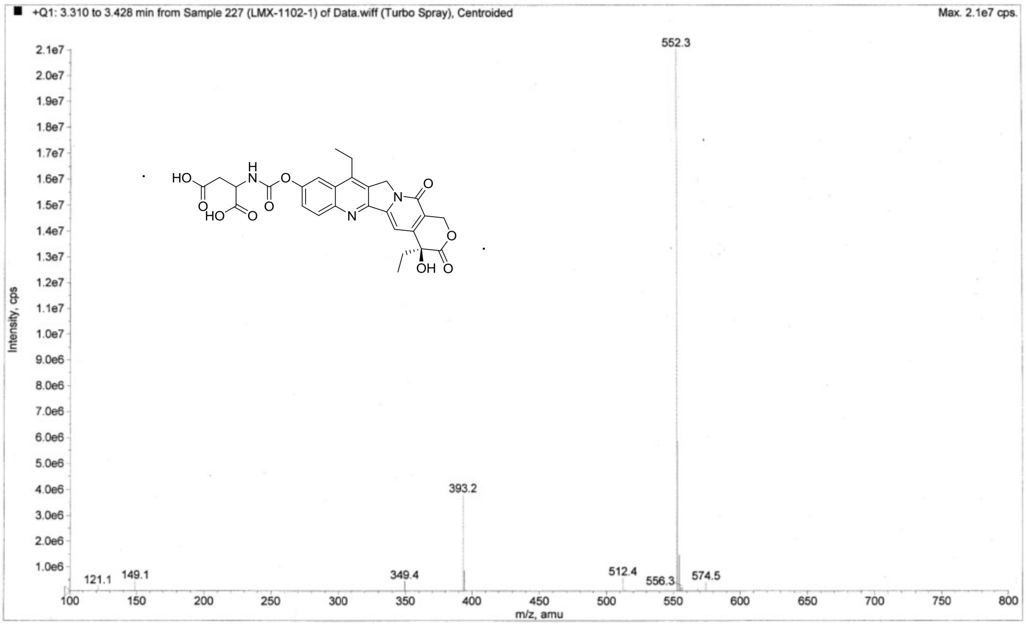

Figure S29. ^{1}H-NMR spectra of **4h**.

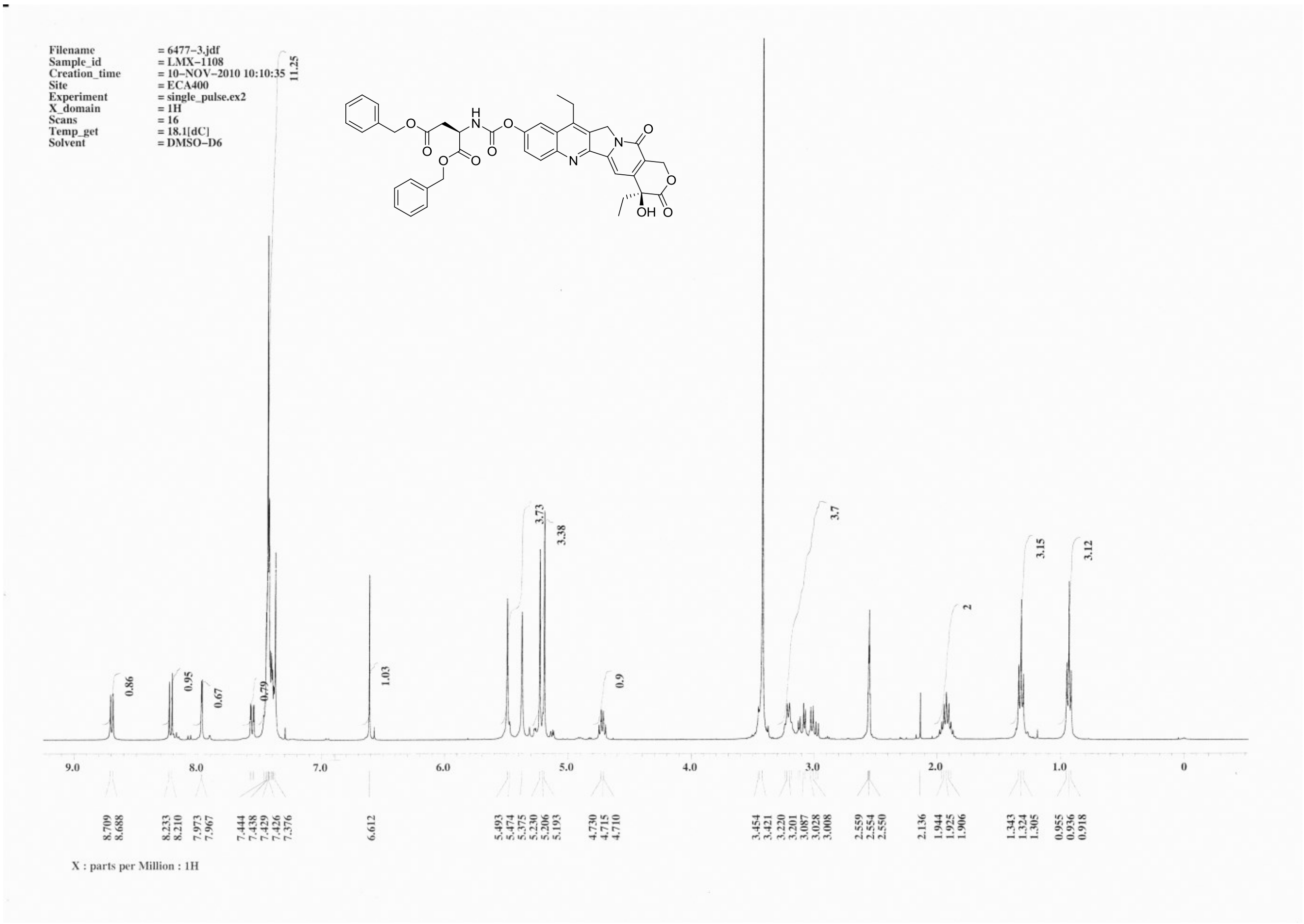

Figure S30. MS spectra of **4h**.

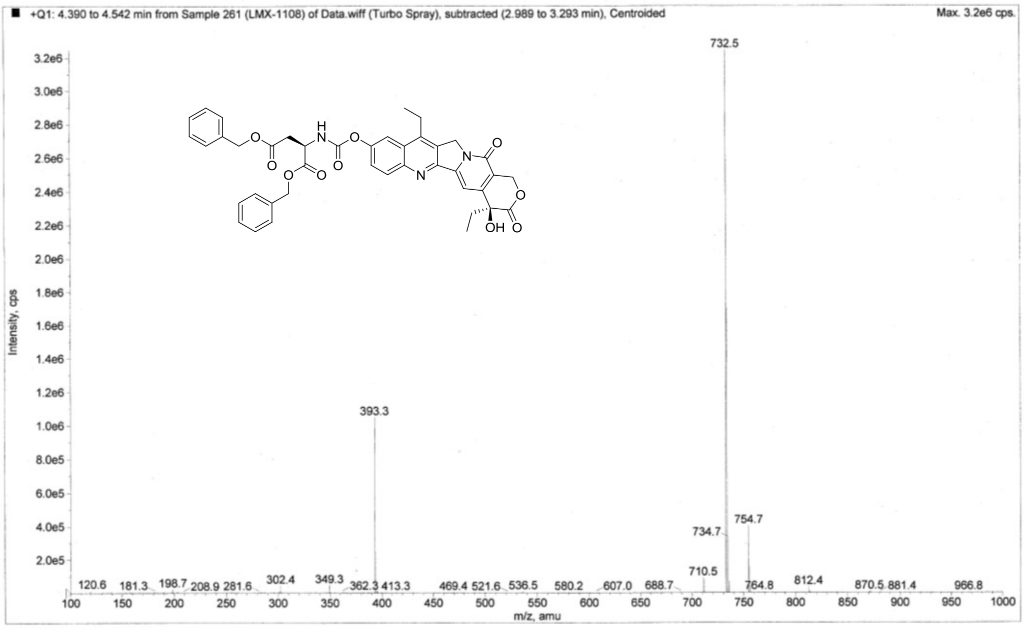

Figure S31. ^{1}H-NMR spectra of **5h**.

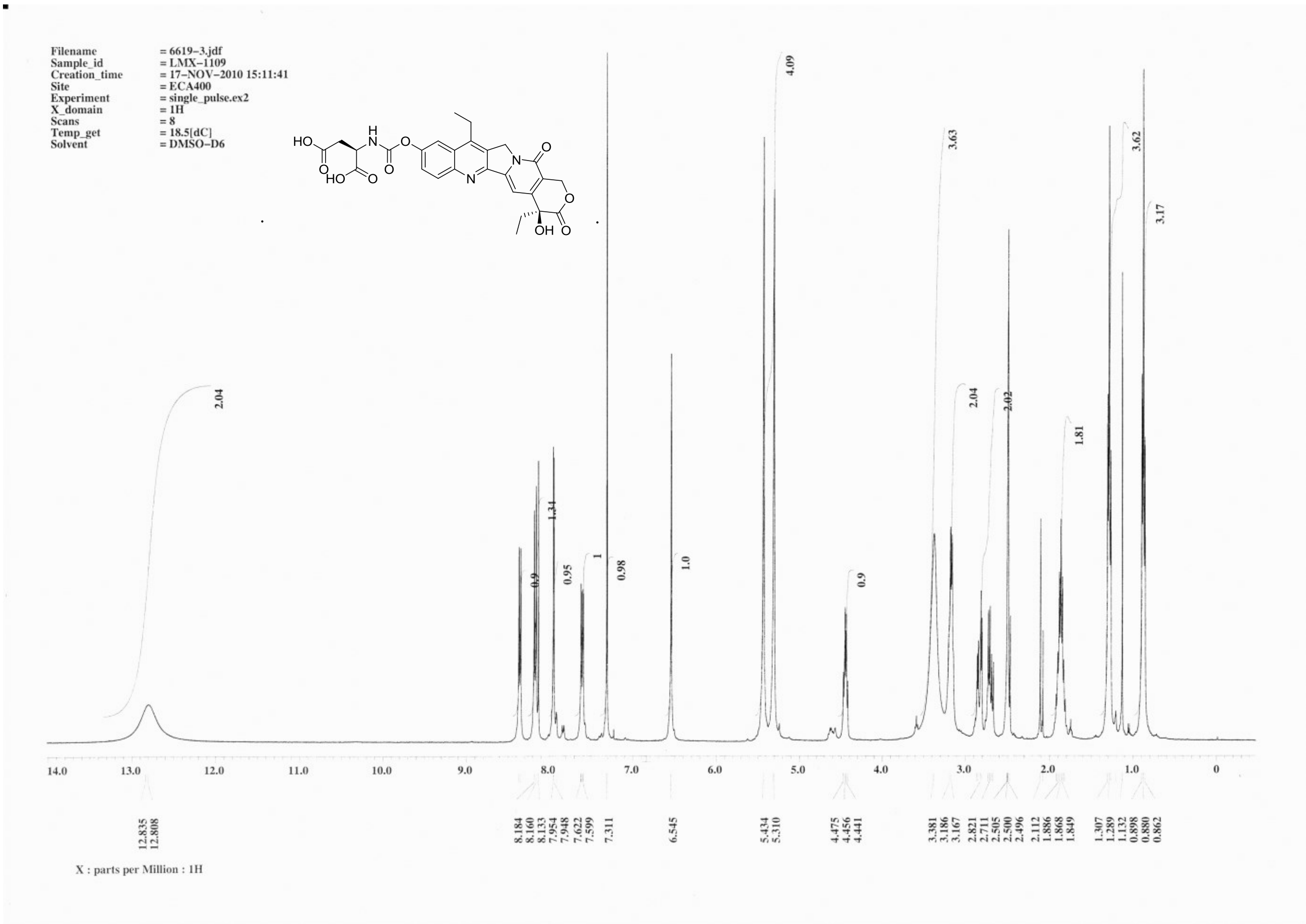

Figure S32. MS spectra of **5h**.

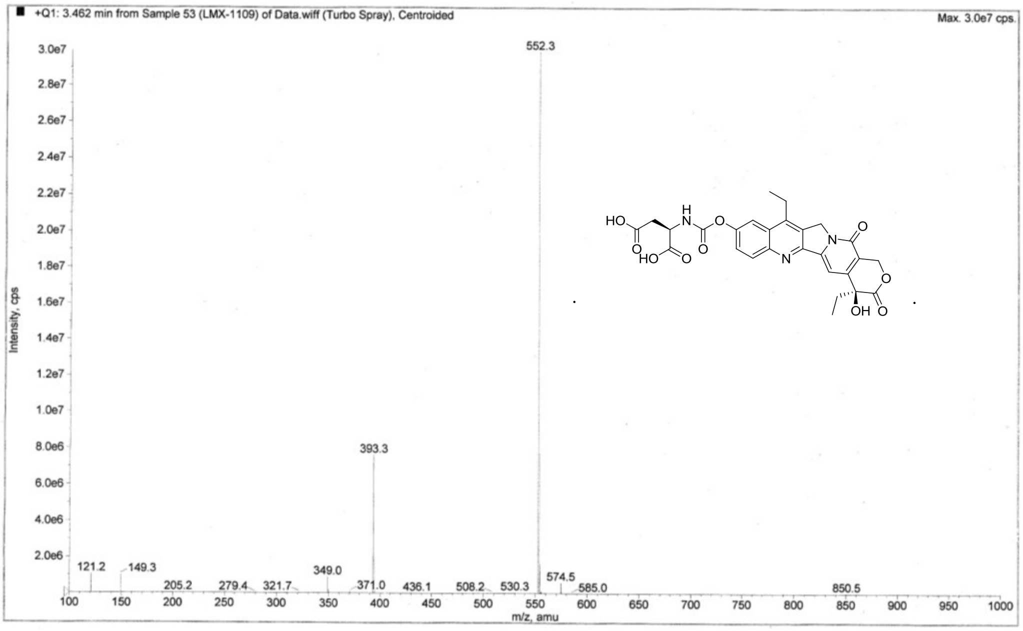

Figure S33. ^{1}H-NMR spectra of **6a**.

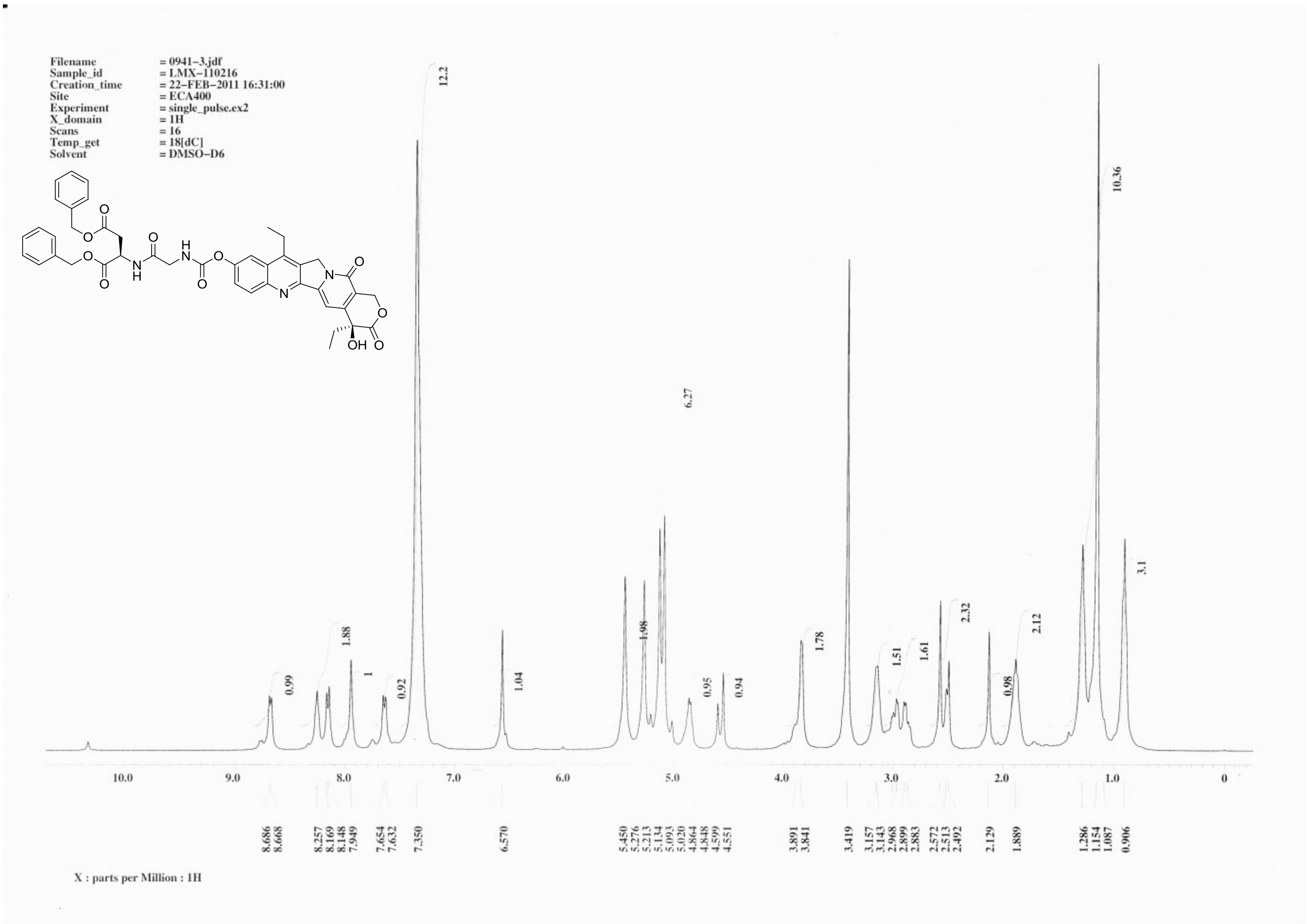

Figure S34. MS spectra of **6a**.

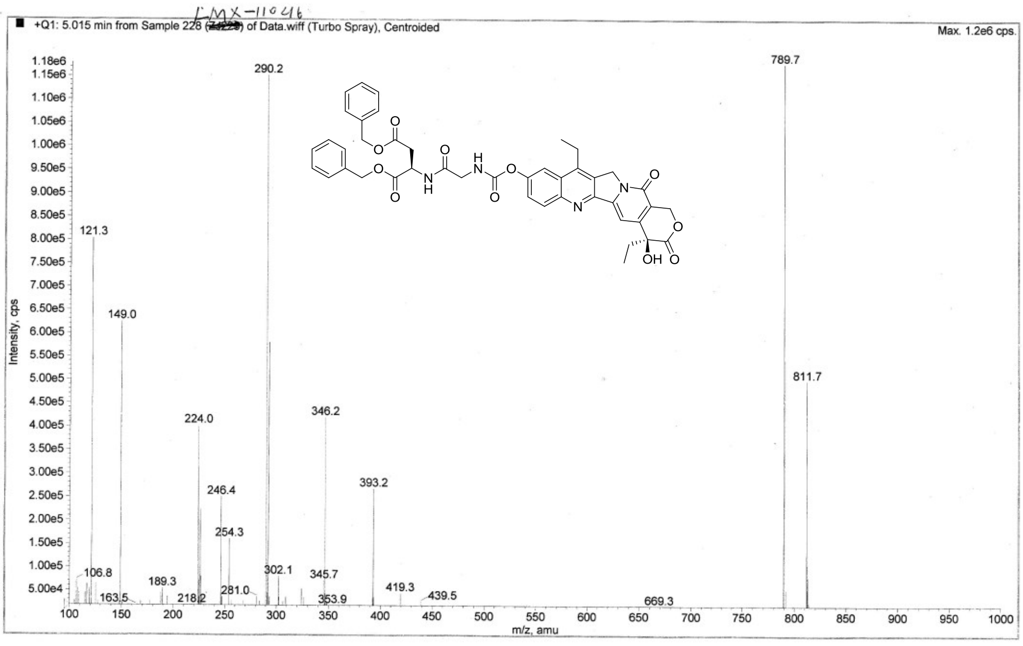

Figure S35. ^{1}H-NMR spectra of **7a**.

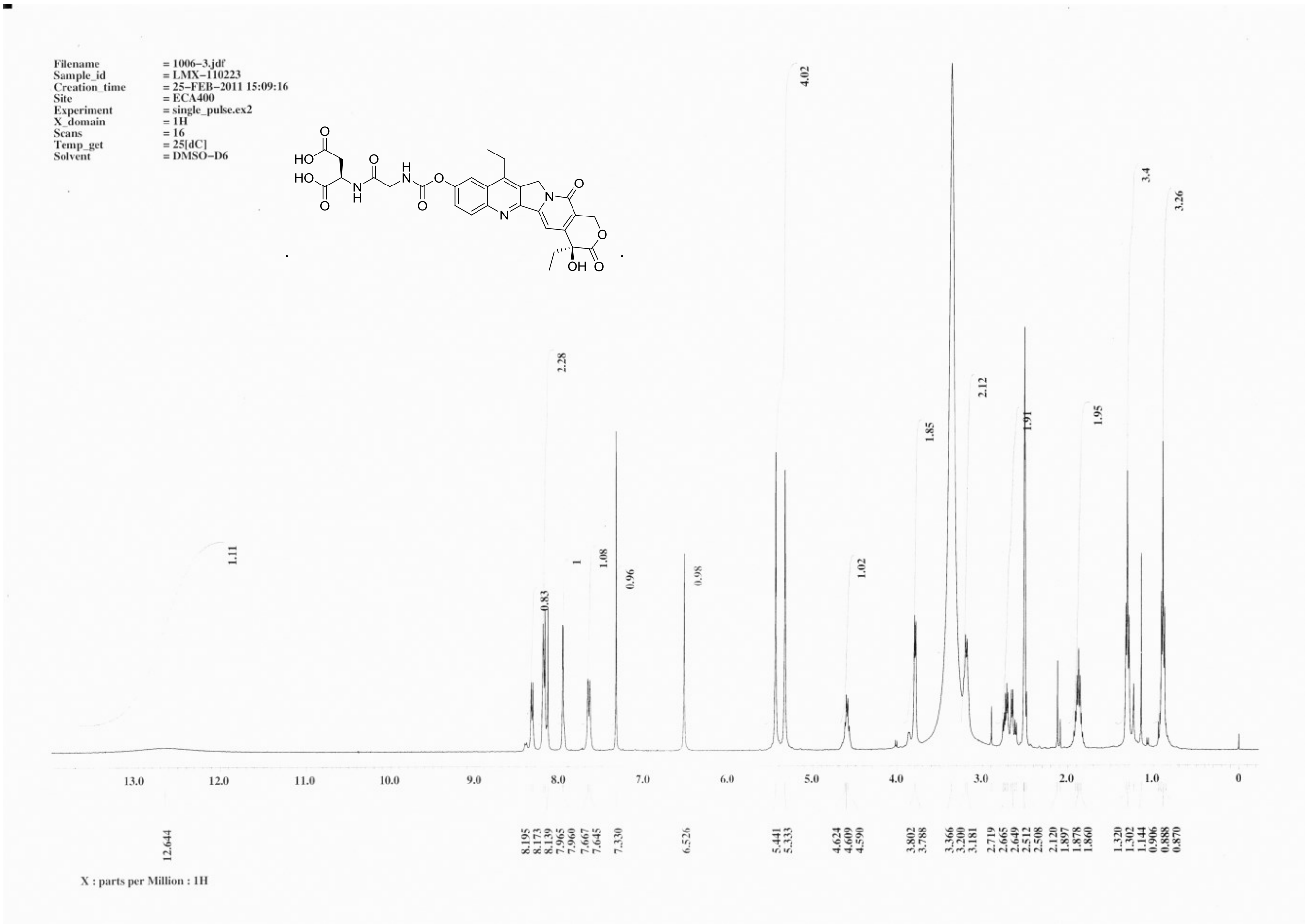

Figure S36. MS spectra of **7a**.

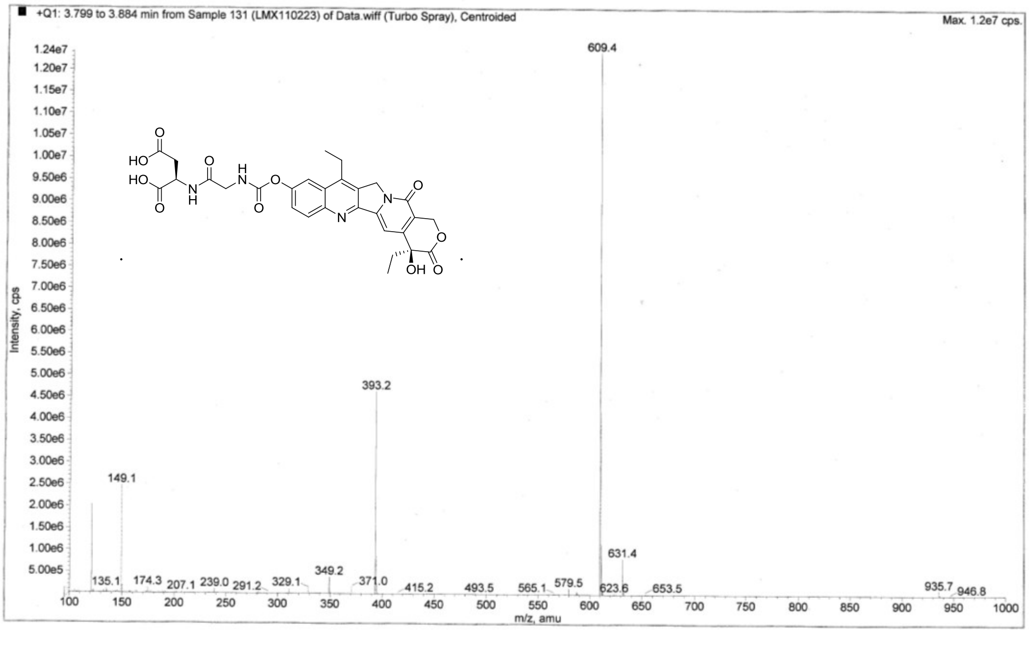

Figure S37. ^{1}H-NMR spectra of **6b**.

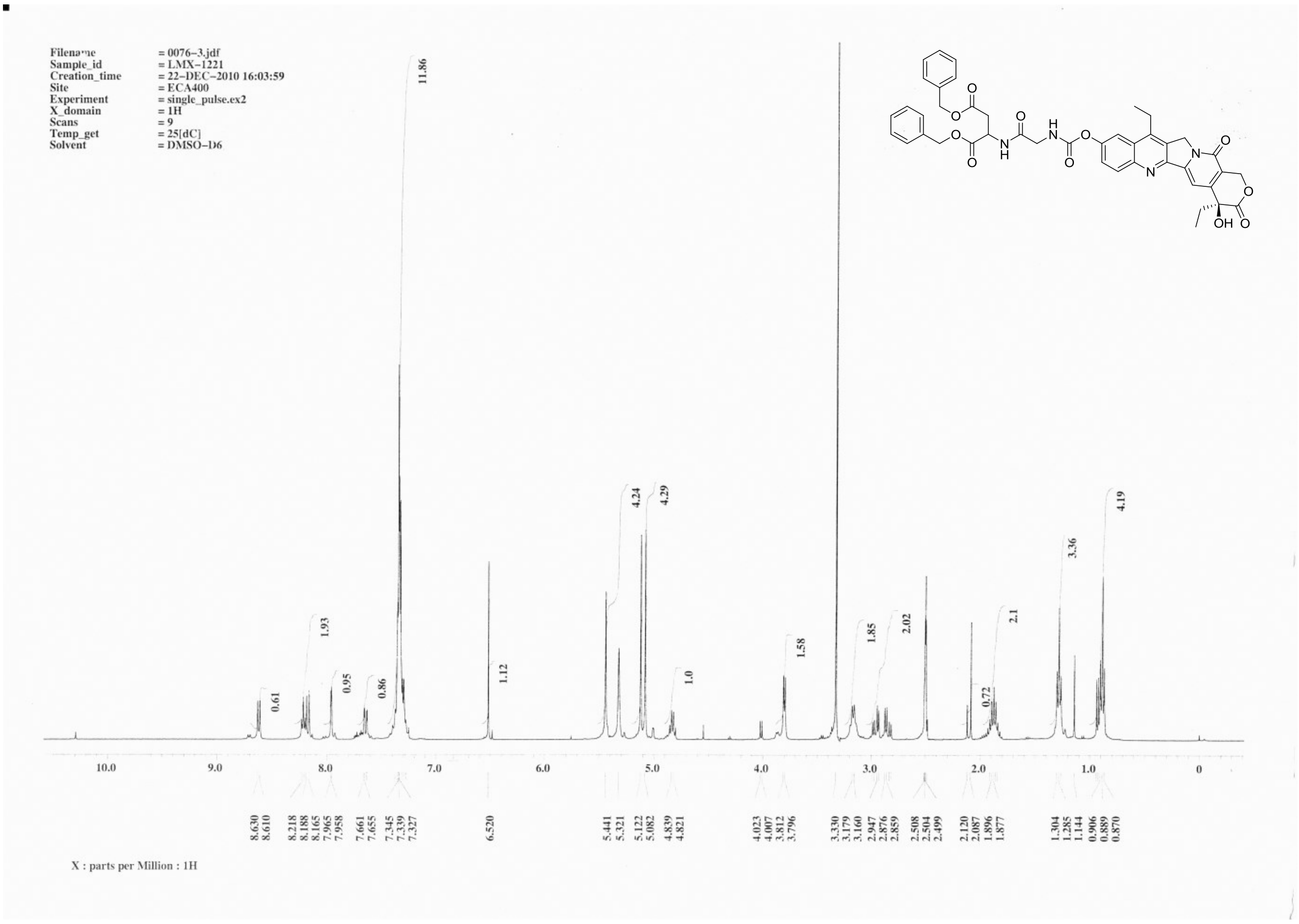

Figure S38. MS spectra of **6b**.

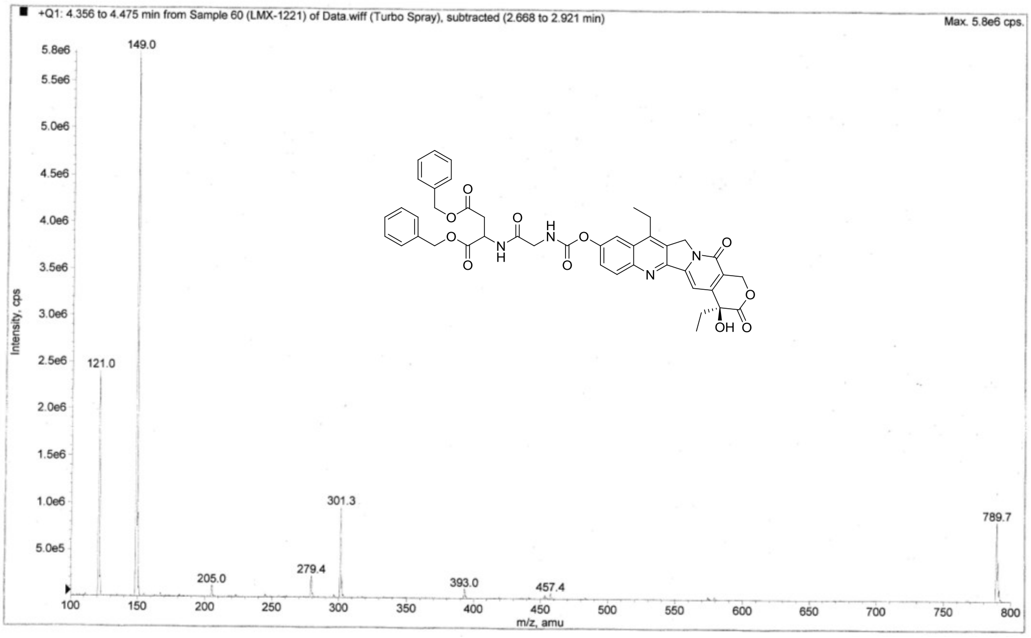

Figure S39. ^{1}H-NMR spectra of **7b**.

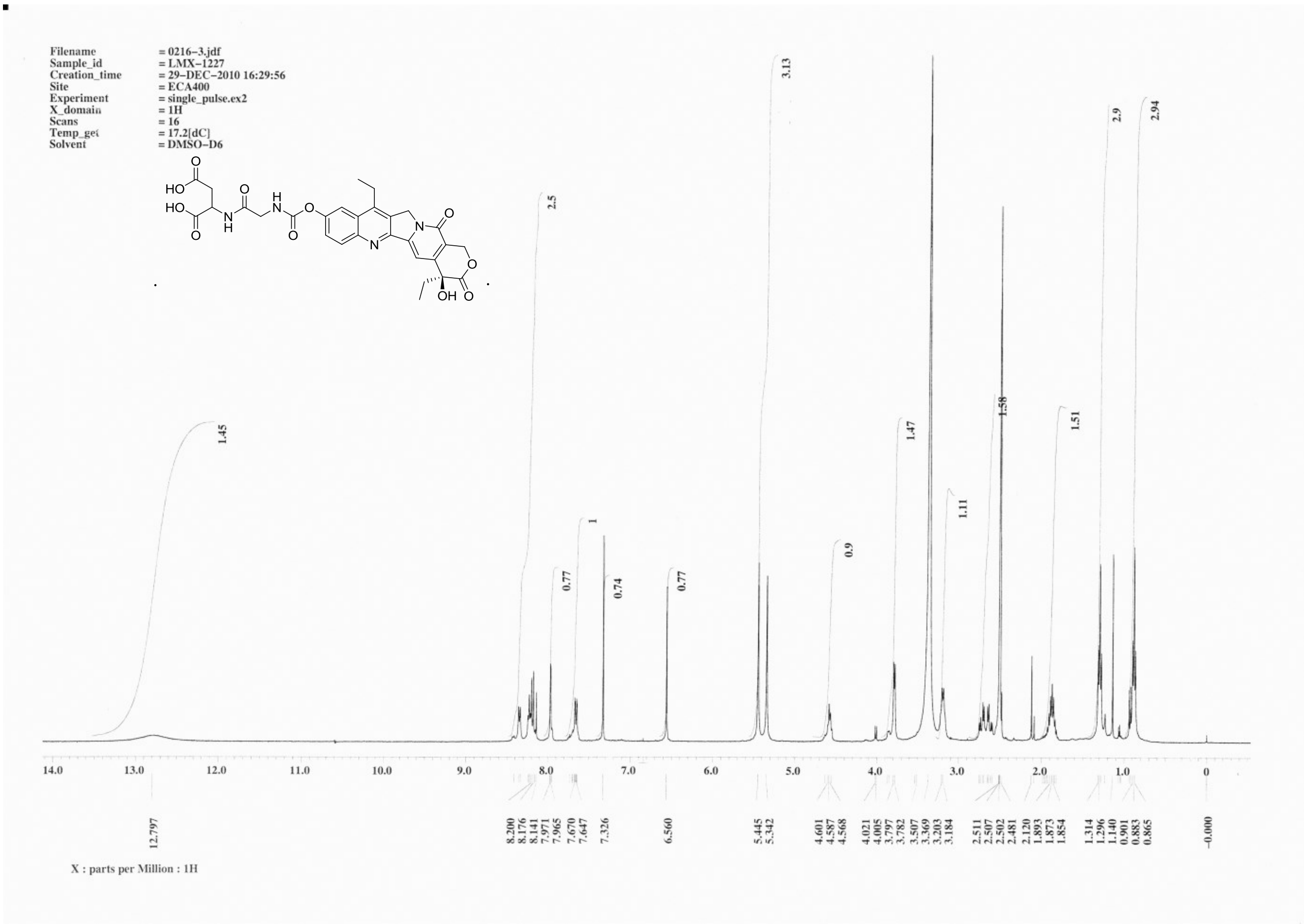

Figure S40. MS spectra of **7b**.

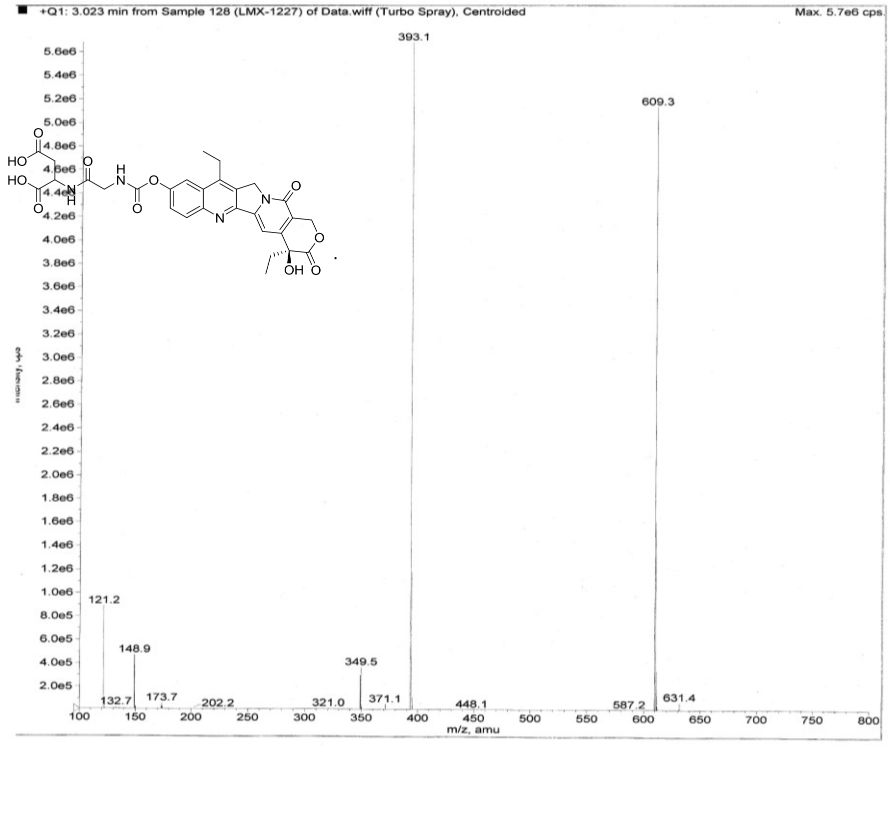

Figure S41. ^{1}H-NMR spectra of **6c**.

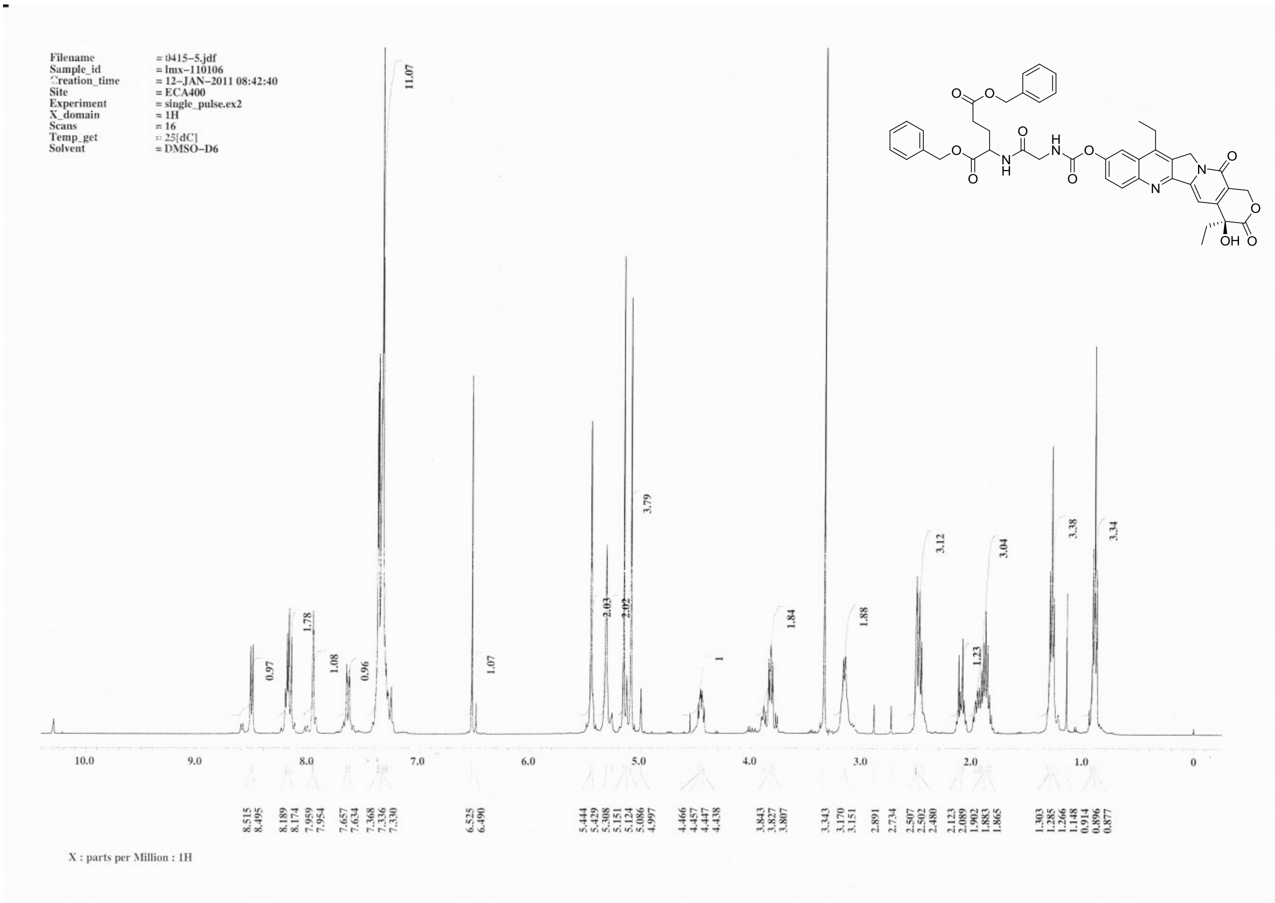

Figure S42. ^{1}H-NMR spectra of **7c**.

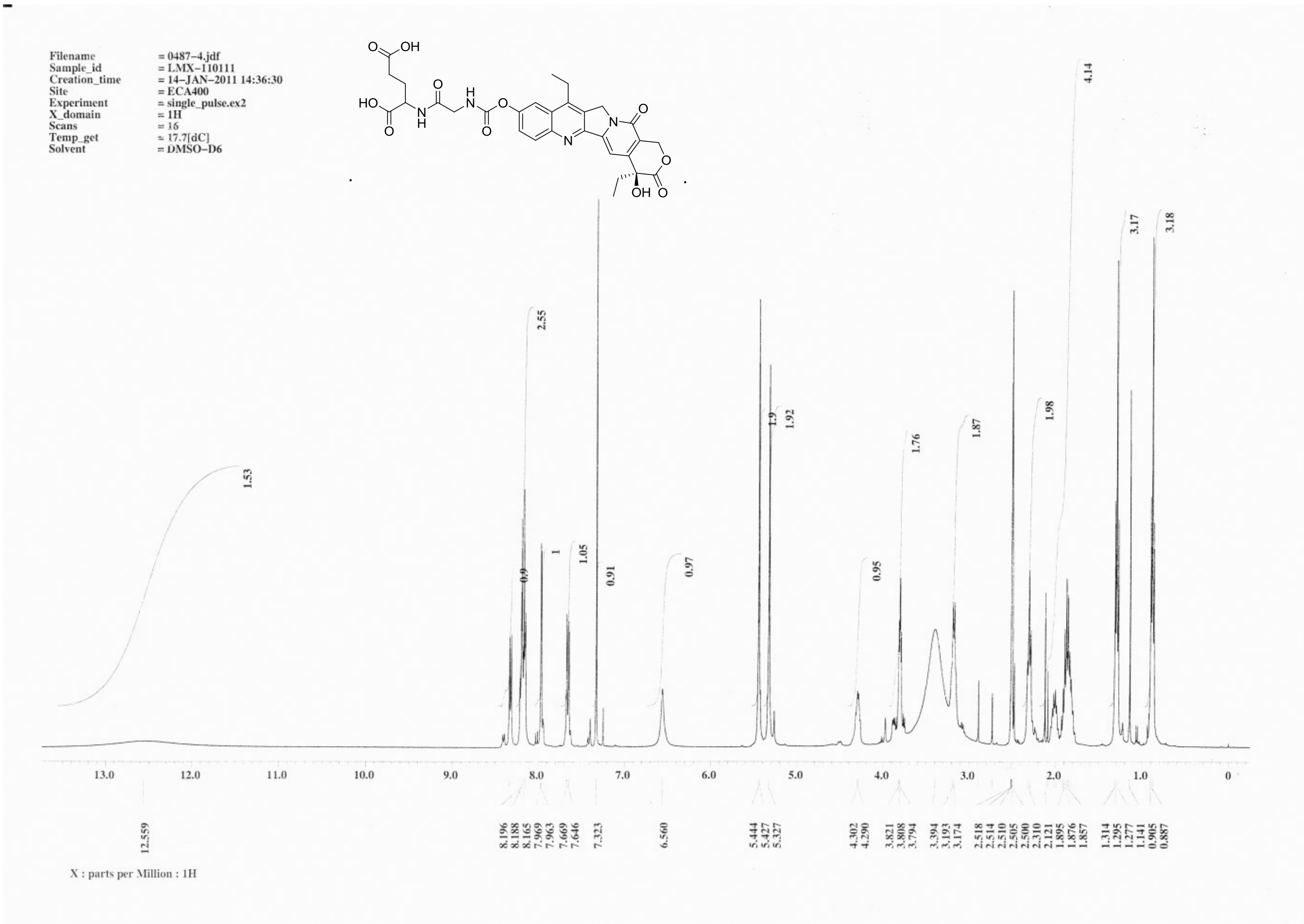

Figure S43. MS spectra of **7c**.

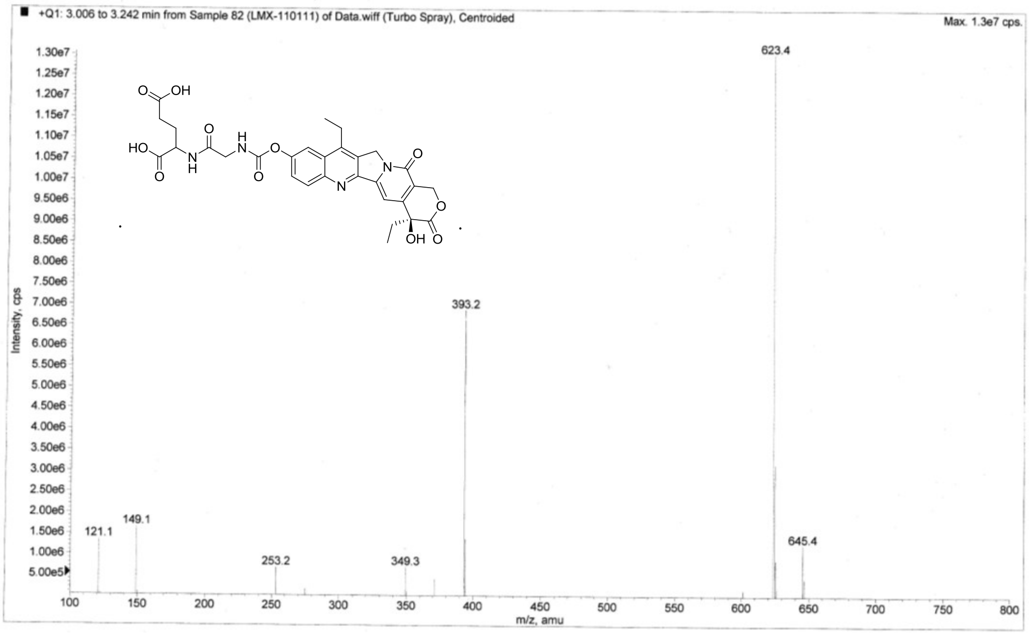

Figure S44. ^{1}H-NMR spectra of **6d**.

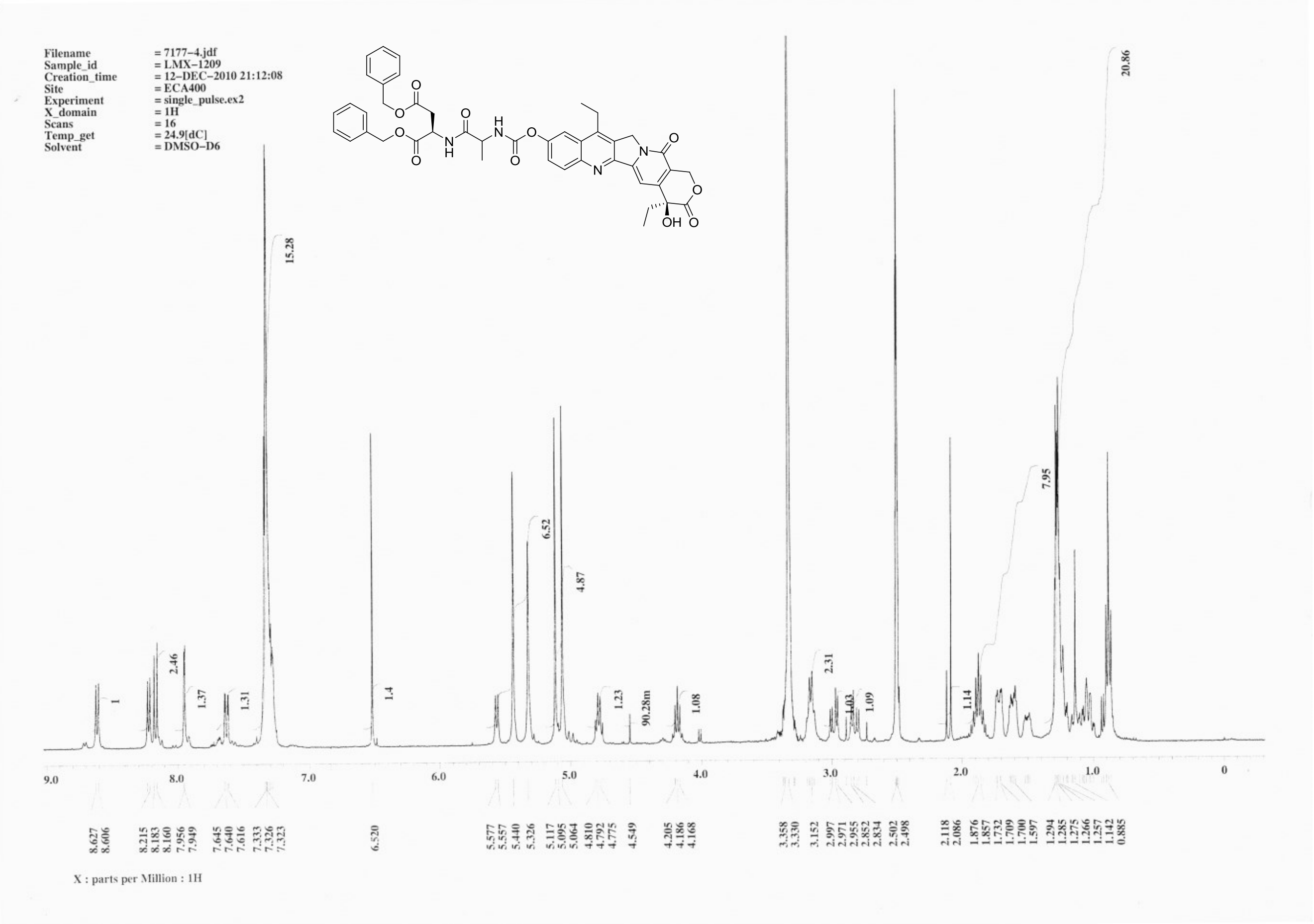

Figure S45. MS spectra for of **6d**.

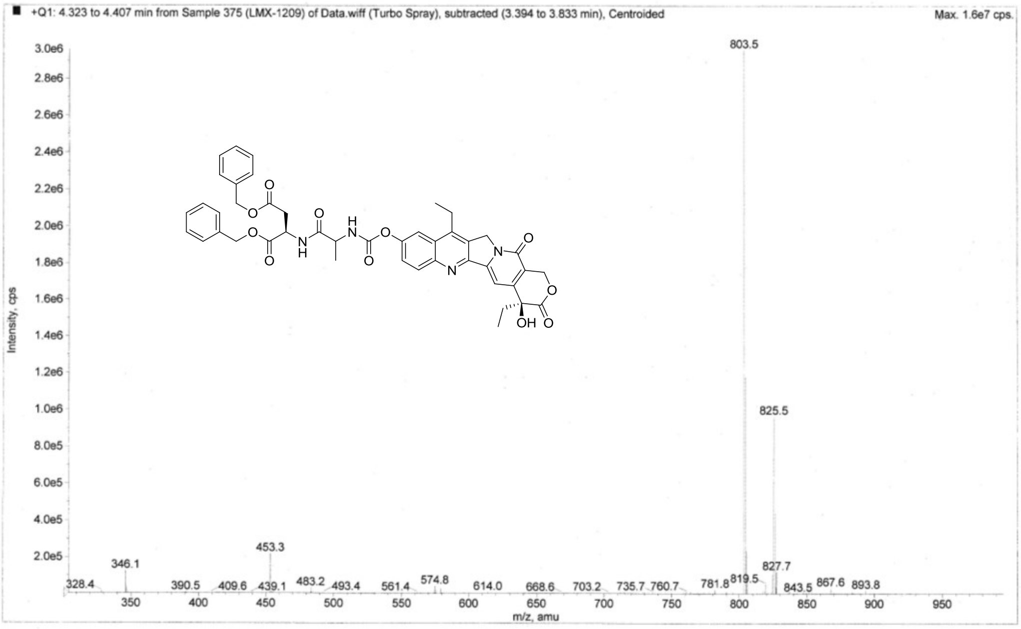

Figure S46. ^{1}H-NMR spectra of **7d**.

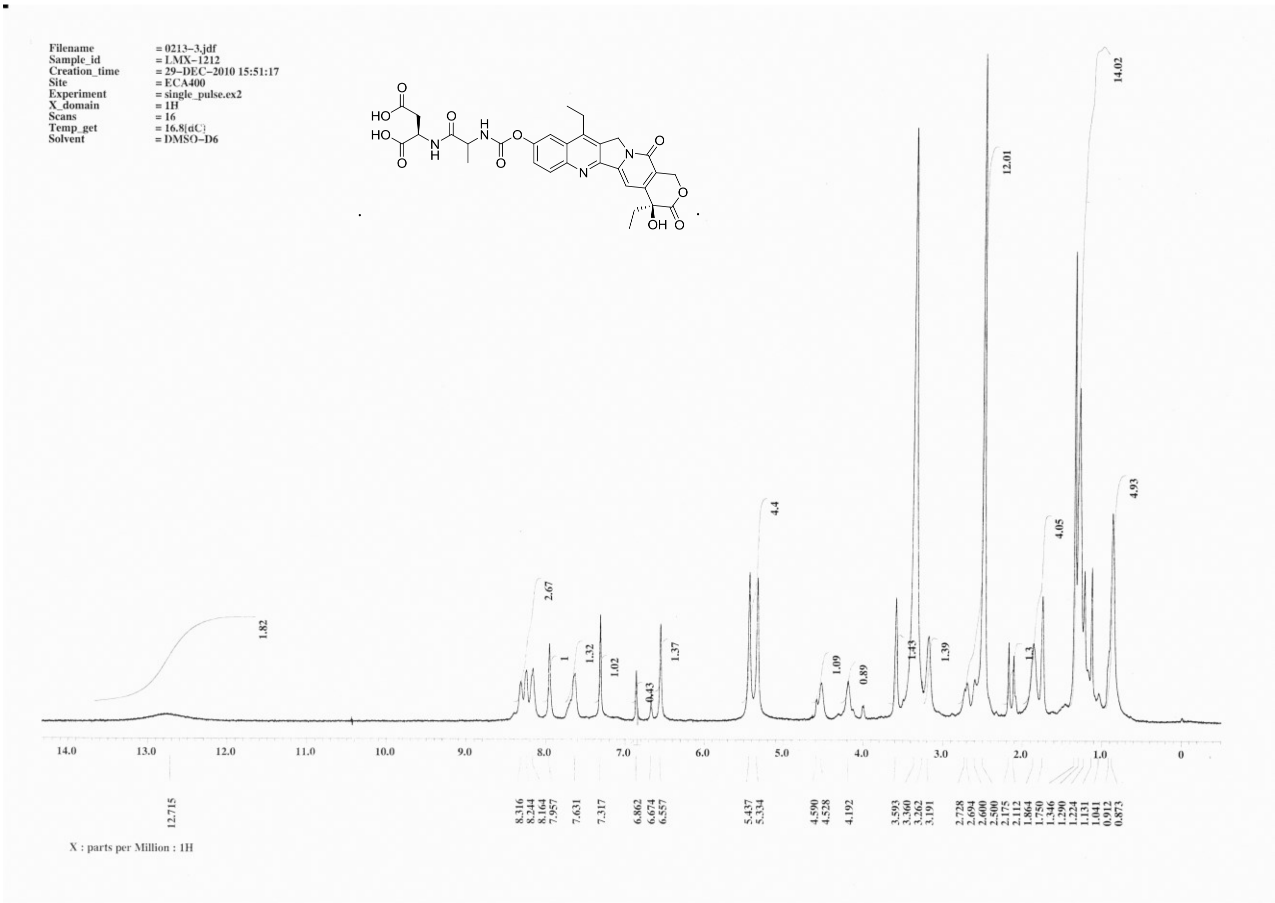

Figure S47. MS spectra of **7d**.

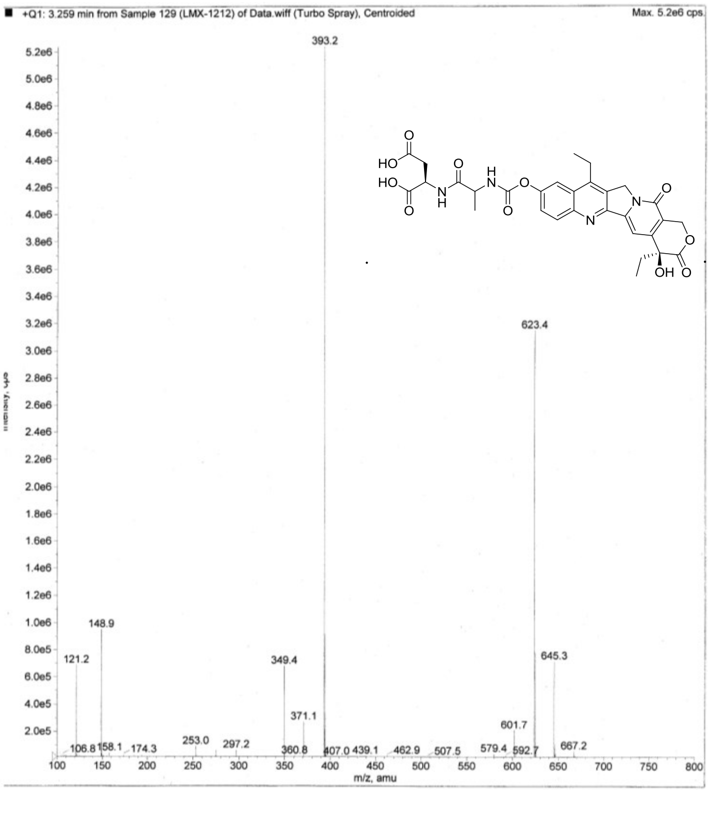

Figure S48. ^{1}H-NMR spectra of **6e**.

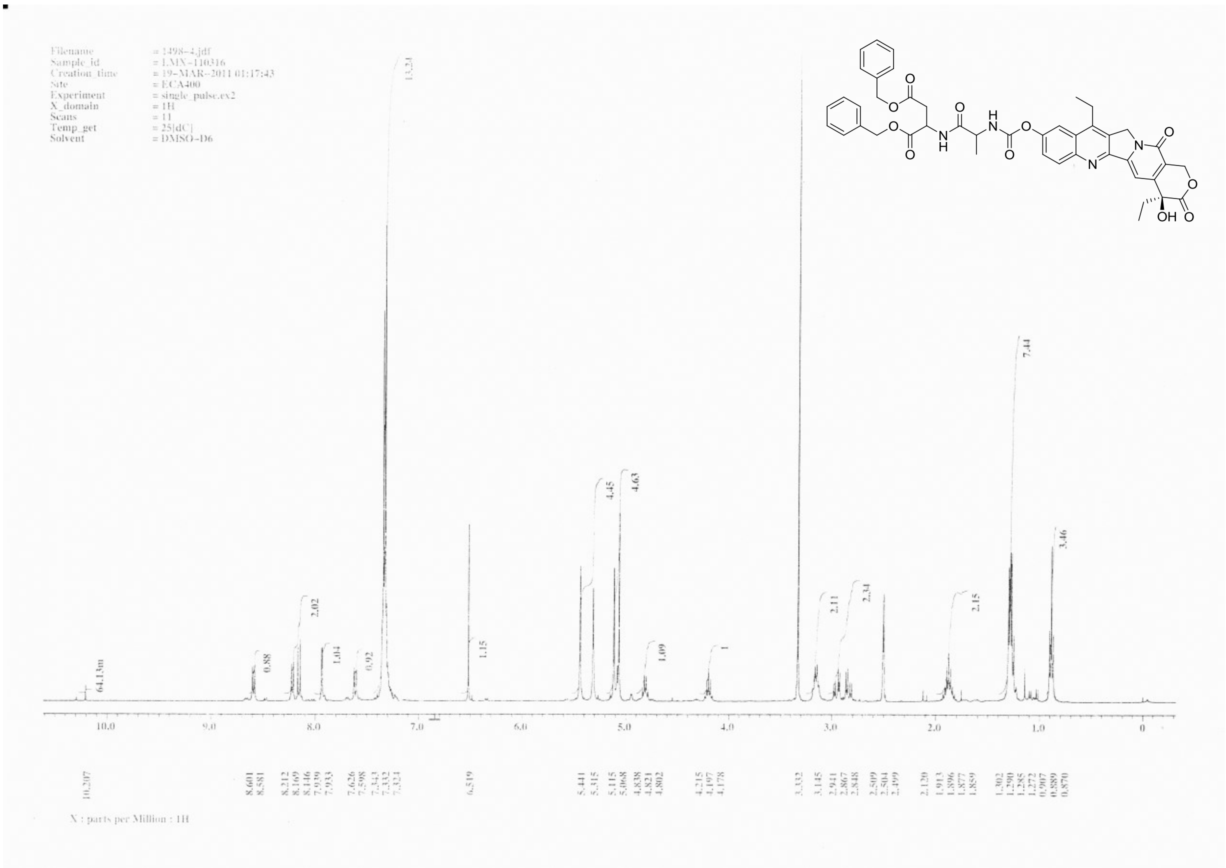

Figure S49. MS spectra of **6e**.

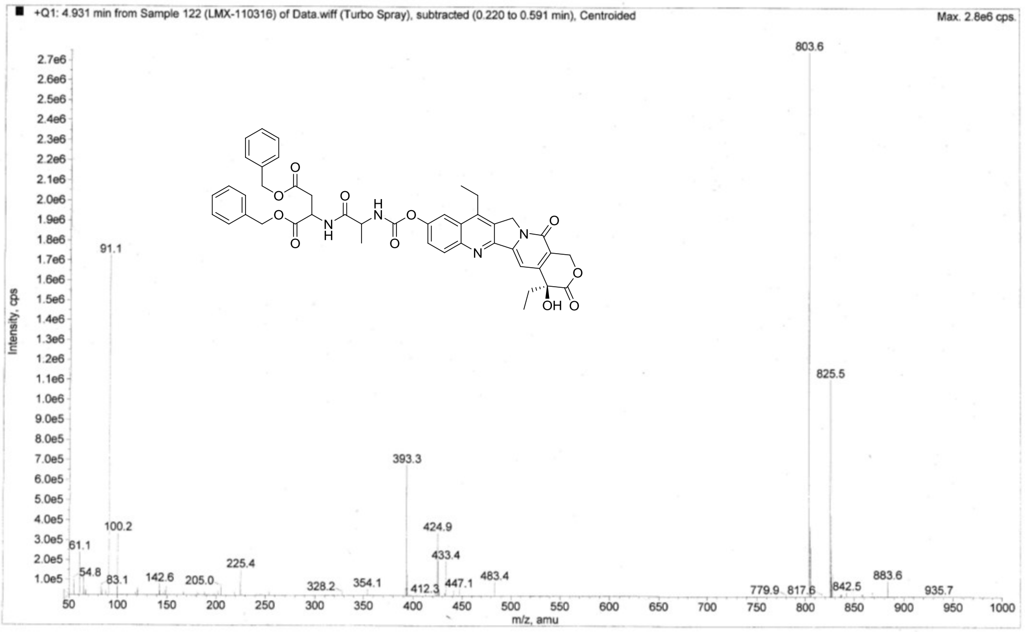

Figure S50. ^{1}H-NMR spectra of **7e**.

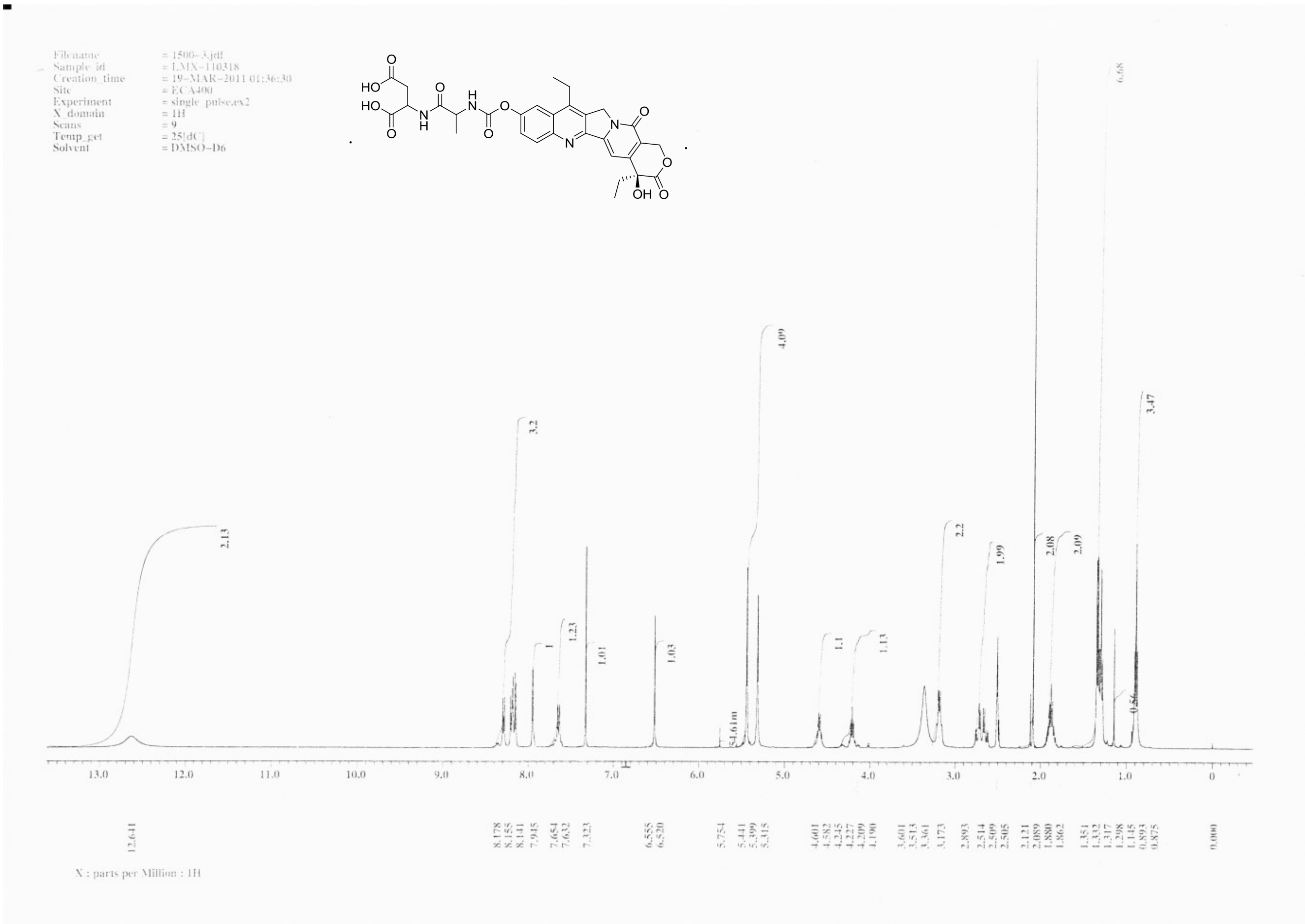

Figure S51. MS spectra of **7e**.

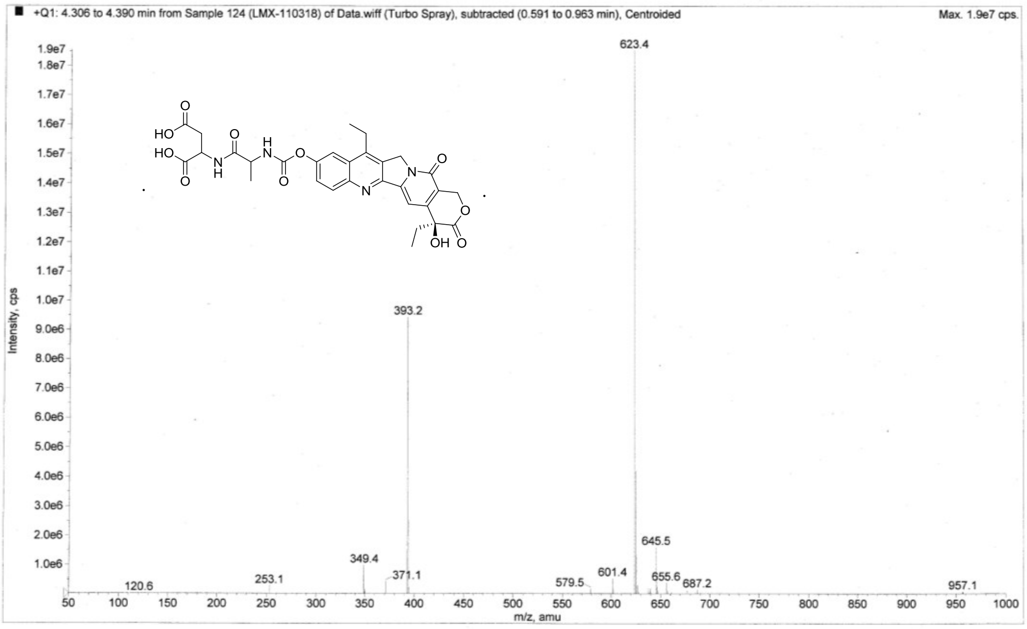

Figure S52. ^{1}H-NMR spectra of **6f**.

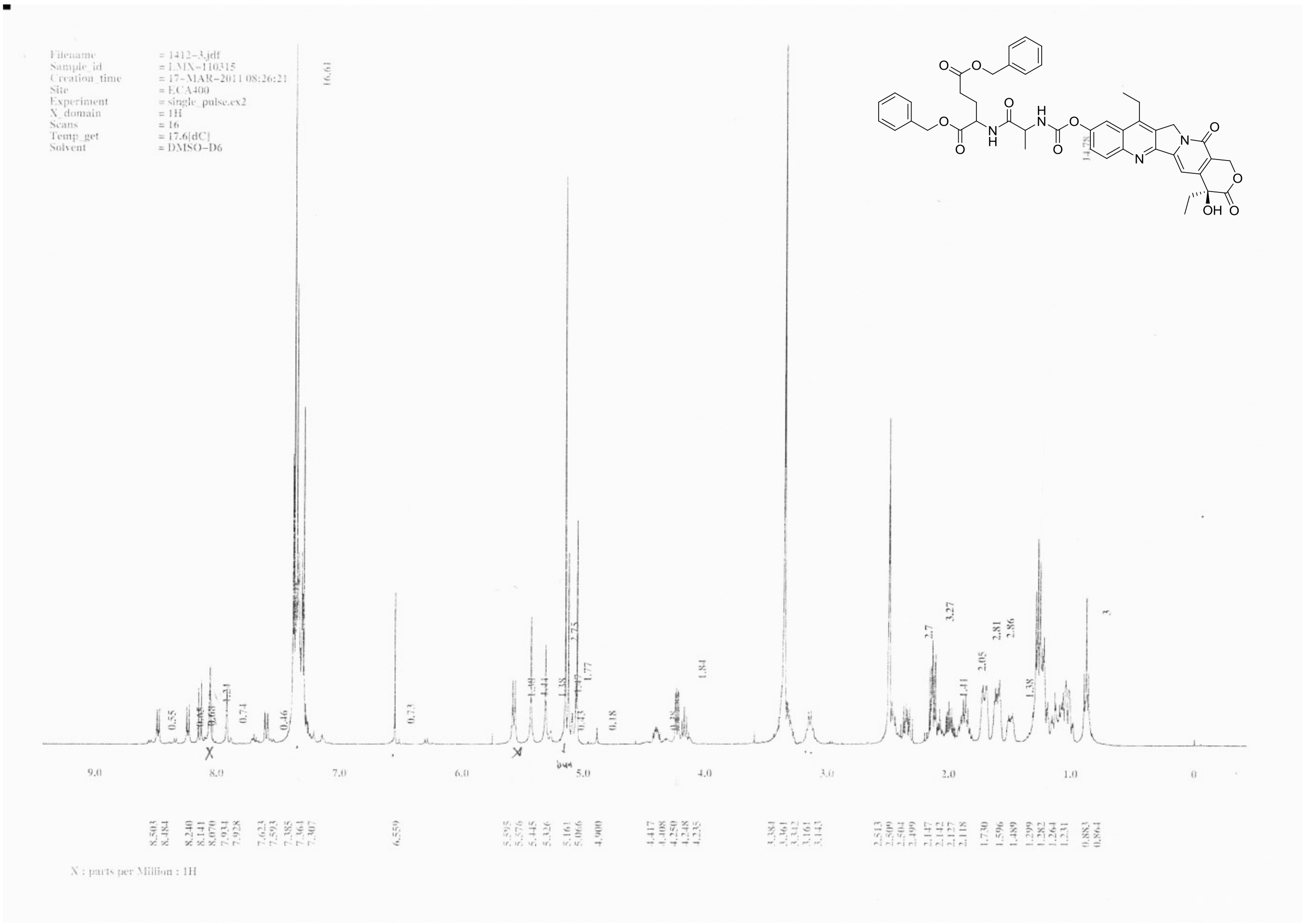

Figure S53. MS spectra of **6f**.

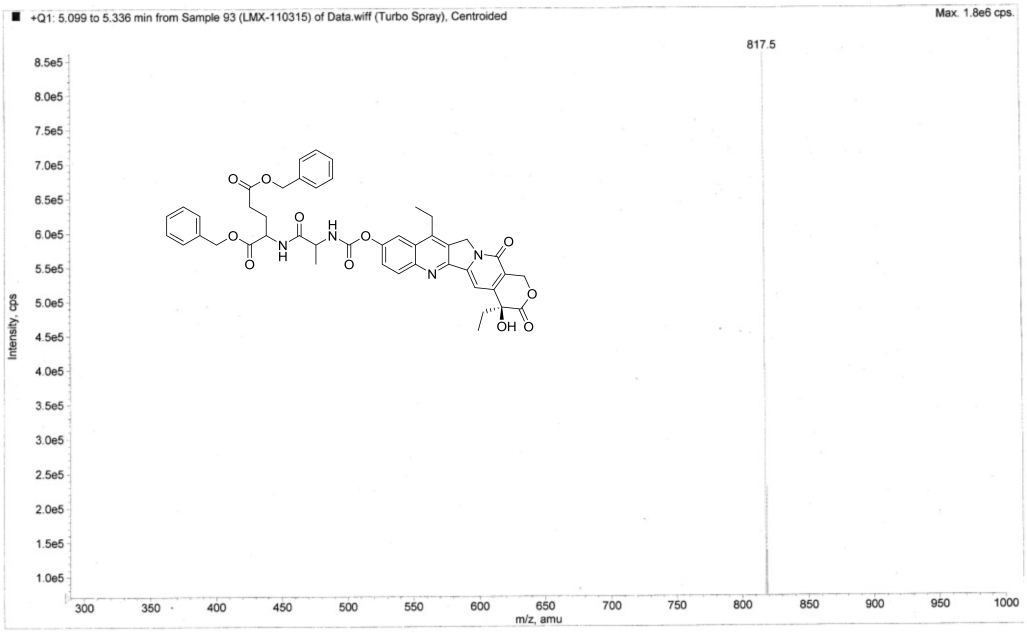

Figure S54. ^{1}H-NMR spectra of **7f**.

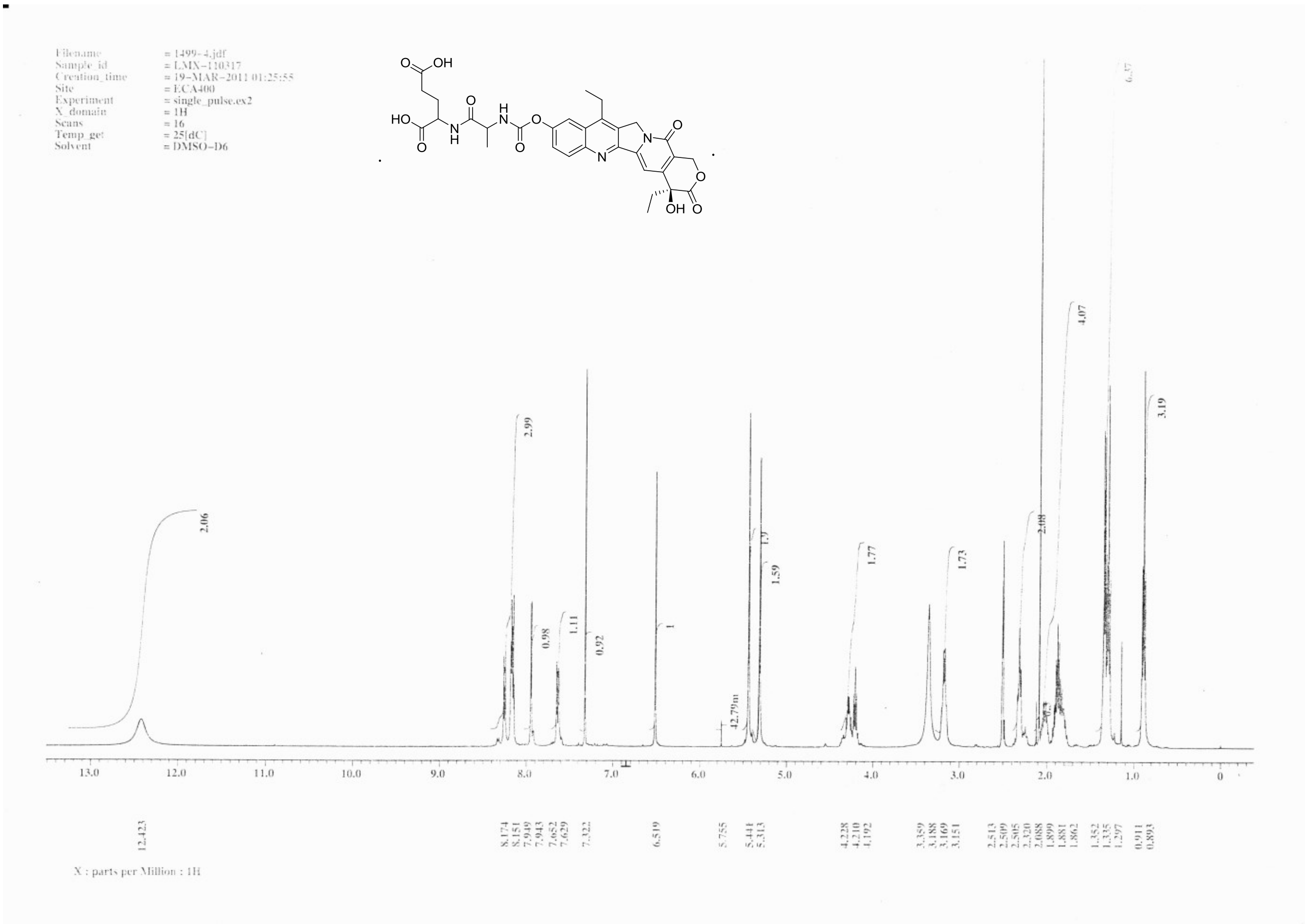

Figure S55. MS spectra of **7f**.

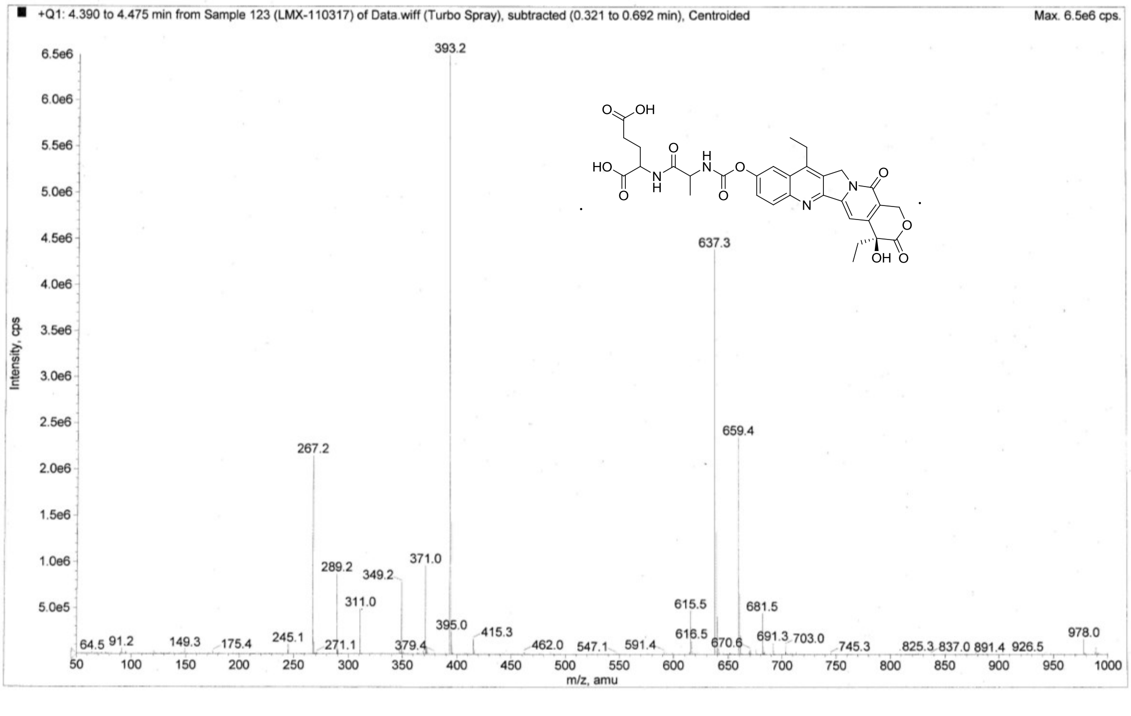

Figure S56. HRMS spectra of **5a**.

Qualitative Analysis Report

**Data Filename**	866-5.d	**Sample Name**	5a
**Instrument Name**	TOF G6230A	**Acquired Time**	2014.10.22
**Acq Method**	YCL.M	**Acquired SW**	6200 series TOF/6500 series
**IRM Calibration Status**	**Success**		

User Chormatograms

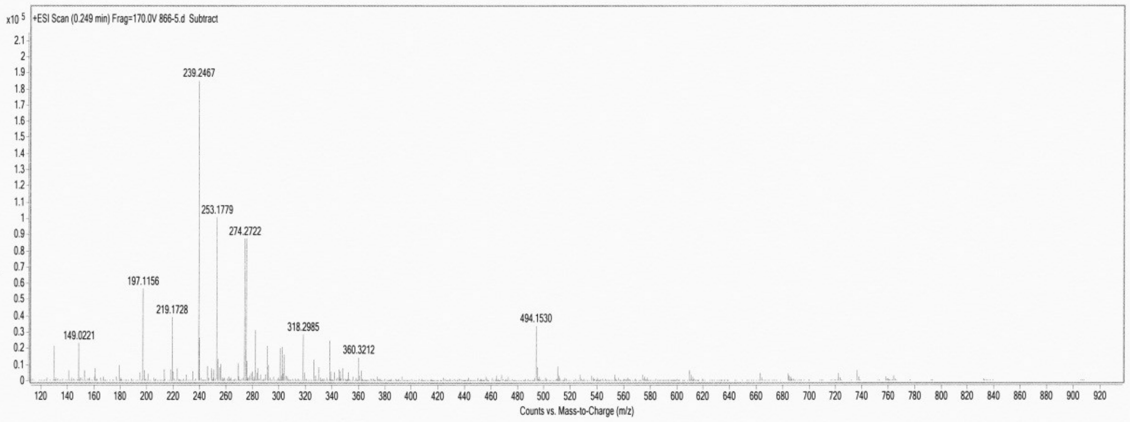

Figure S57. HRMS spectra of **5b**.

Qualitative Analysis Report

**Data Filename**	853-3.d	**Sample Name**	5b
**Instrument Name**	TOF G6230A	**Acquired Time**	2014.10.21
**Acq Method**	YCL.M	**Acquired SW**	6200 series TOF/6500 series
**IRM Calibration Status**	**Success**		

User Chormatograms

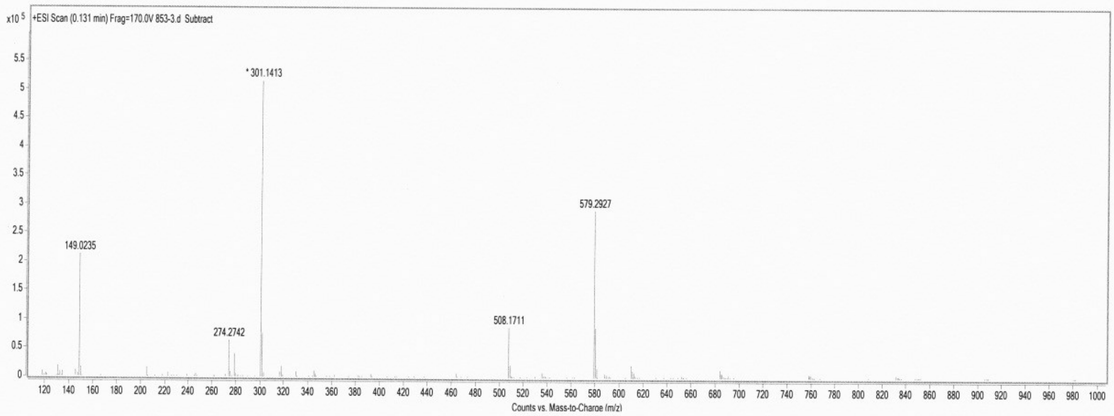

Figure S58. HRMS spectra of **5c**.

Qualitative Analysis Report

**Data Filename**	854-2.d	**Sample Name**	5c
**Instrument Name**	TOF G6230A	**Acquired Time**	2014.10.21
**Acq Method**	YCL.M	**Acquired SW**	6200 series TOF/6500 series
**IRM Calibration Status**	**Success**		

User Chormatograms

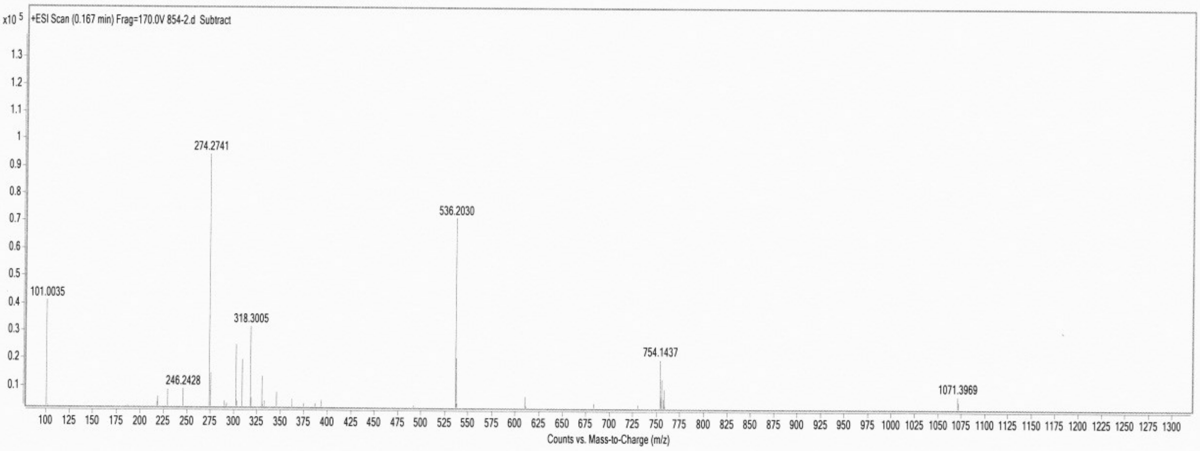

Figure S59. HRMS spectra of **5d**.

Qualitative Analysis Report

**Data Filename**	855-2.d	**Sample Name**	5d
**Instrument Name**	TOF G6230A	**Acquired Time**	2014.10.21
**Acq Method**	YCL.M	**Acquired SW**	6200 series TOF/6500 series
**IRM Calibration Status**	**Success**		

User Chormatograms

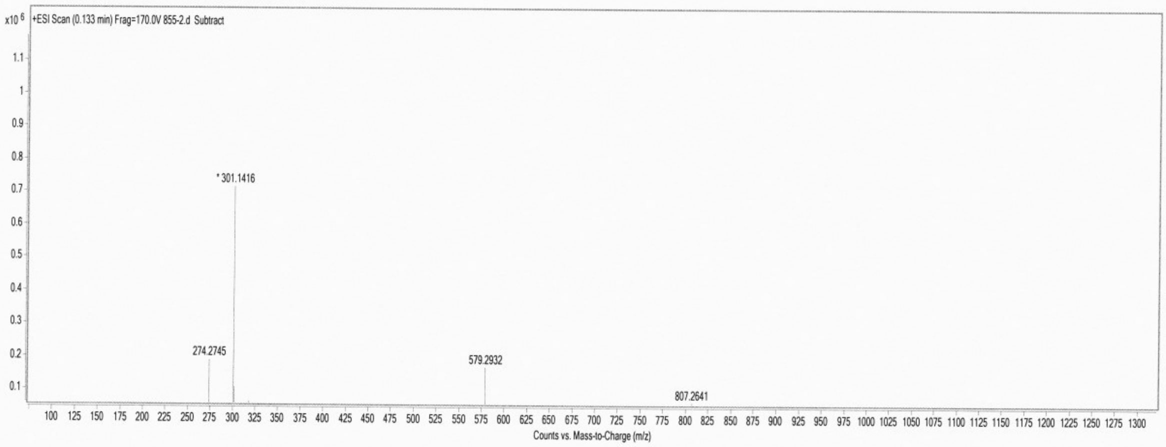

Figure S60. HRMS spectra of **5e**.

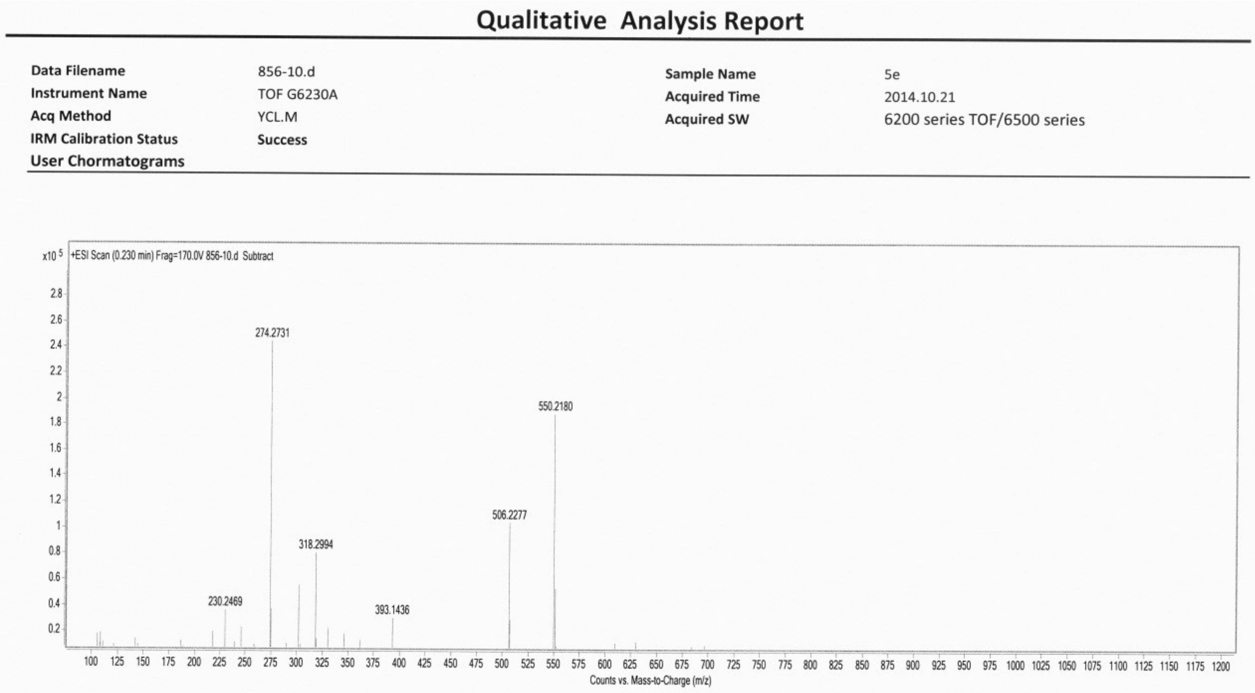

Figure S61. HRMS spectra of **5f**.

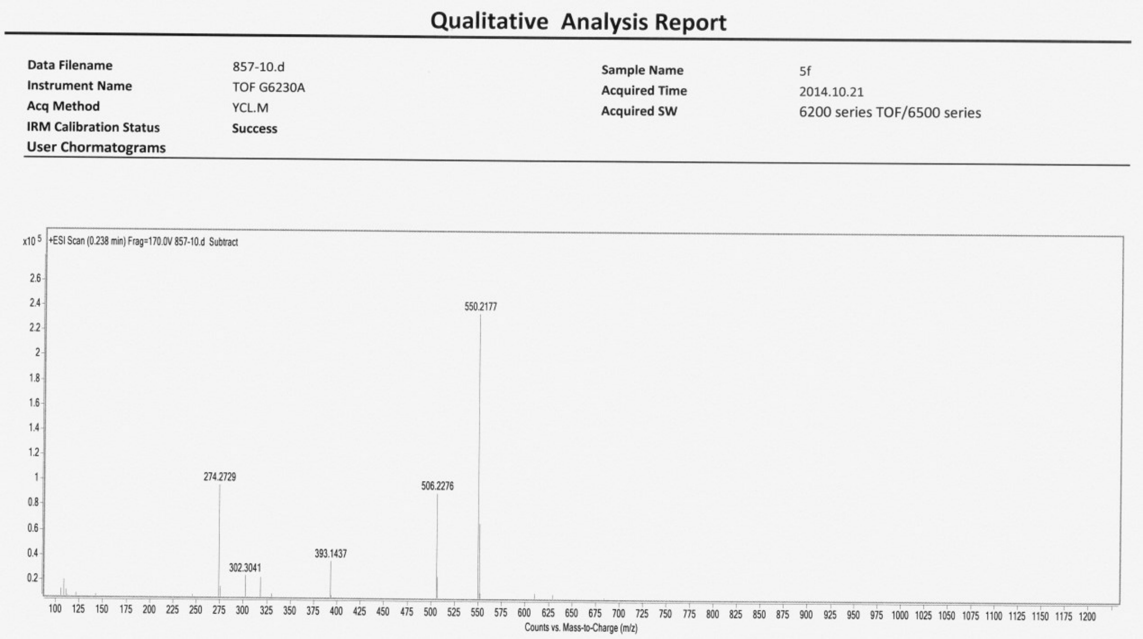

Figure S62. HRMS spectra of **5g**.

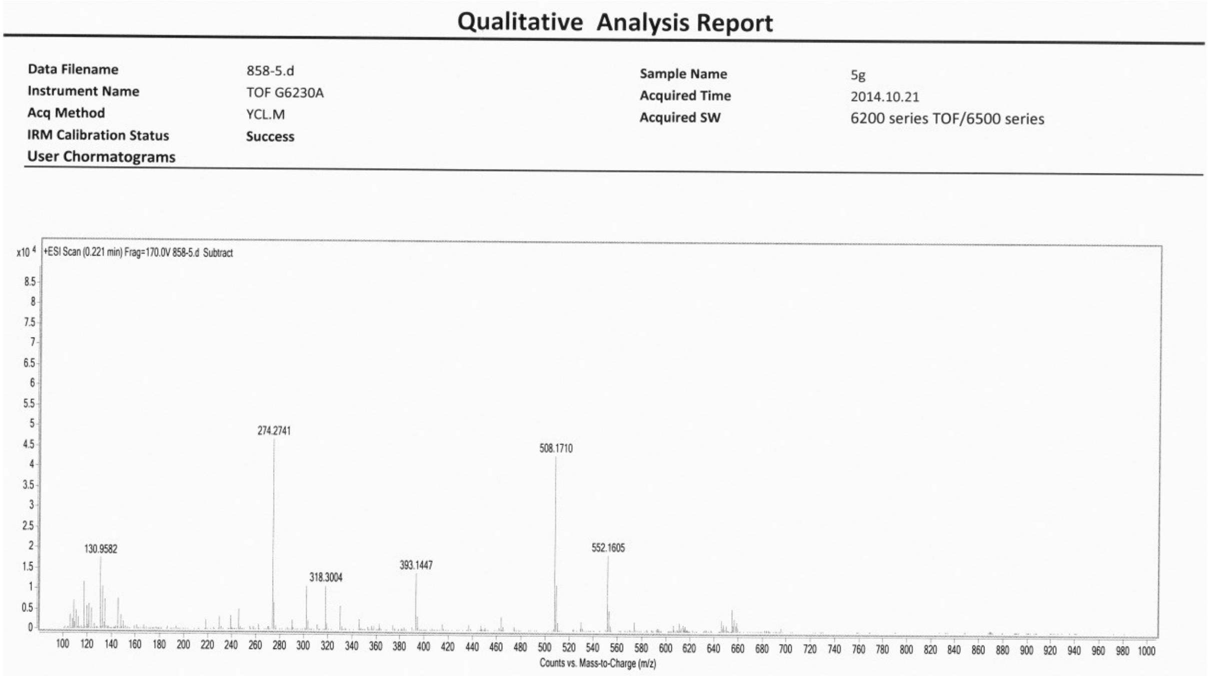

Figure S63. HRMS spectra of **5h**.

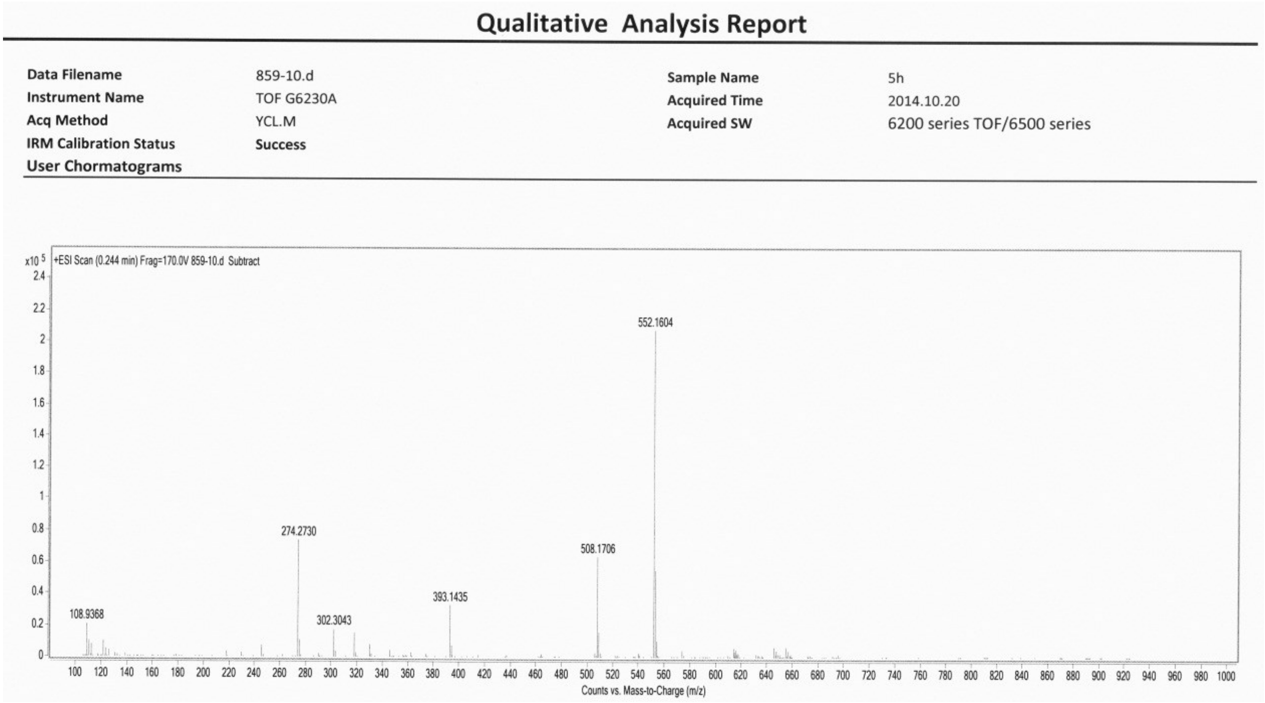

Figure S64. HRMS spectra of **7a**.

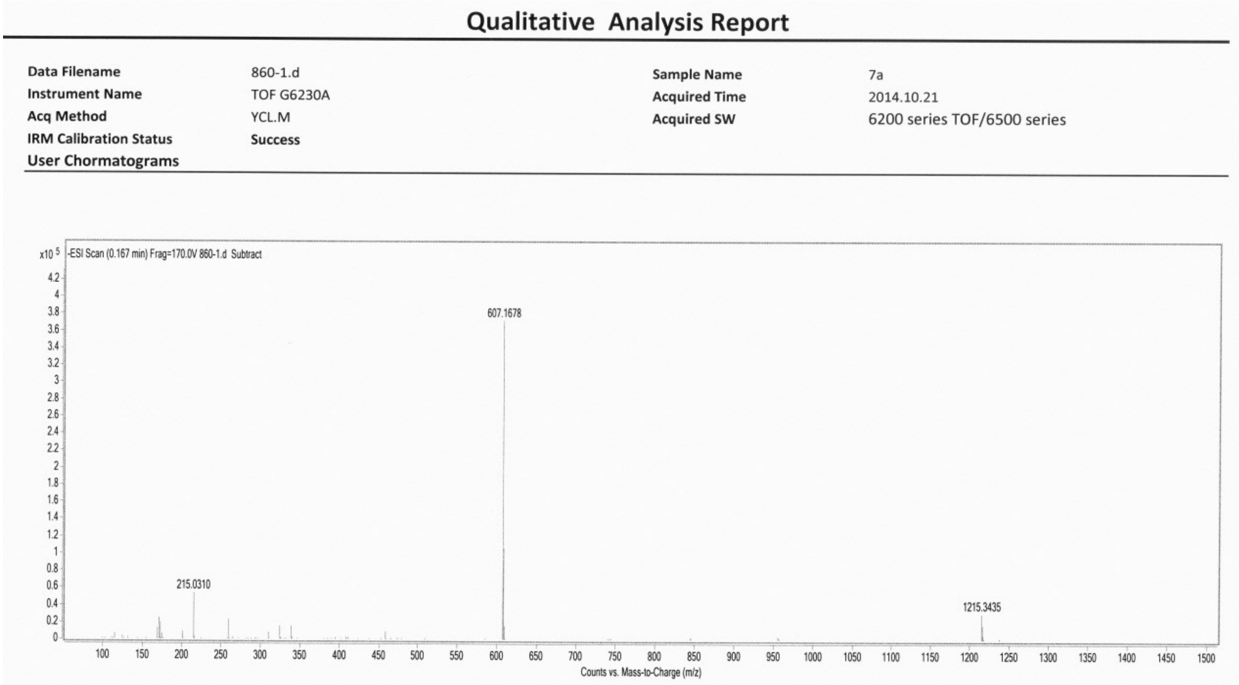

Figure S65. HRMS spectra of **7b**.

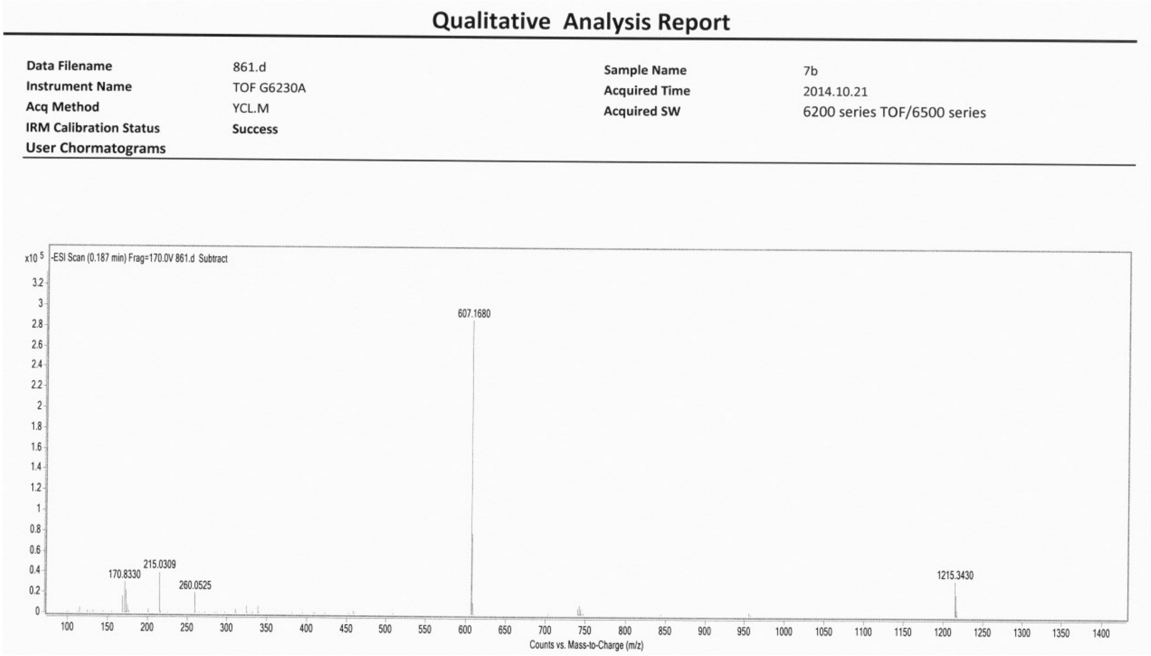

Figure S66. HRMS spectra of **7c**.

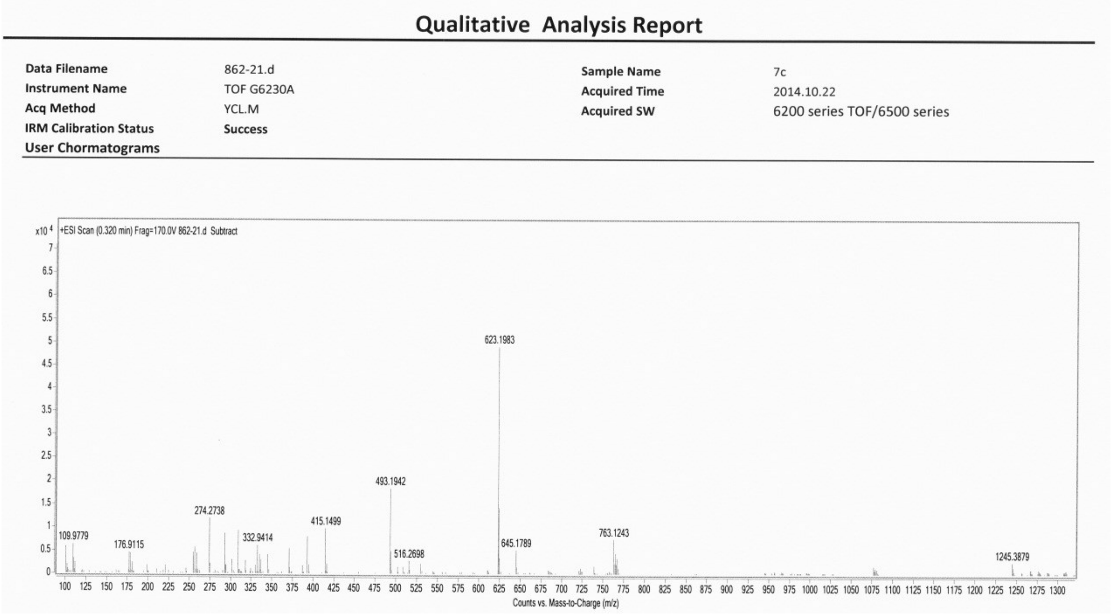

Figure S67. HRMS spectra of **7d**.

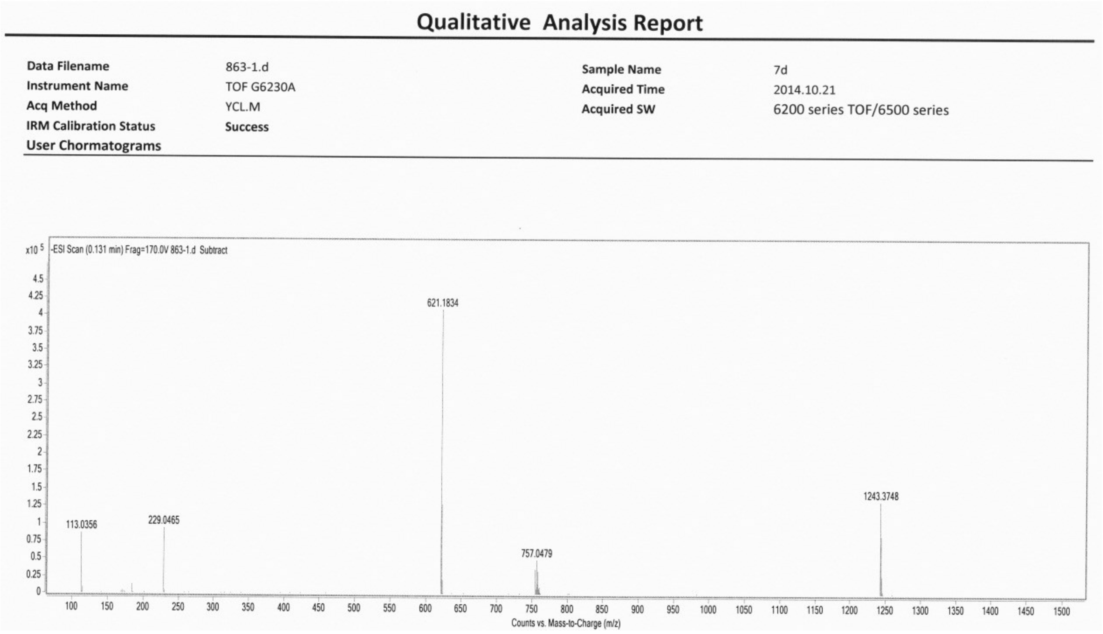

Figure S68. HRMS spectra of **7e**.

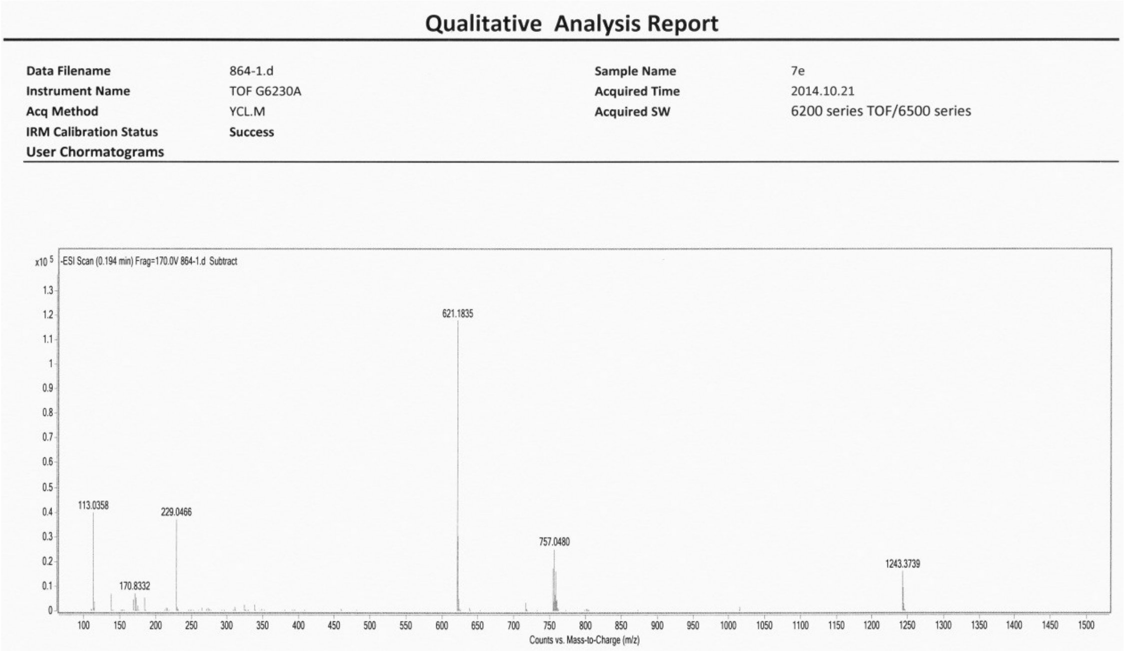

Figure S69. HRMS spectra of **7f**.

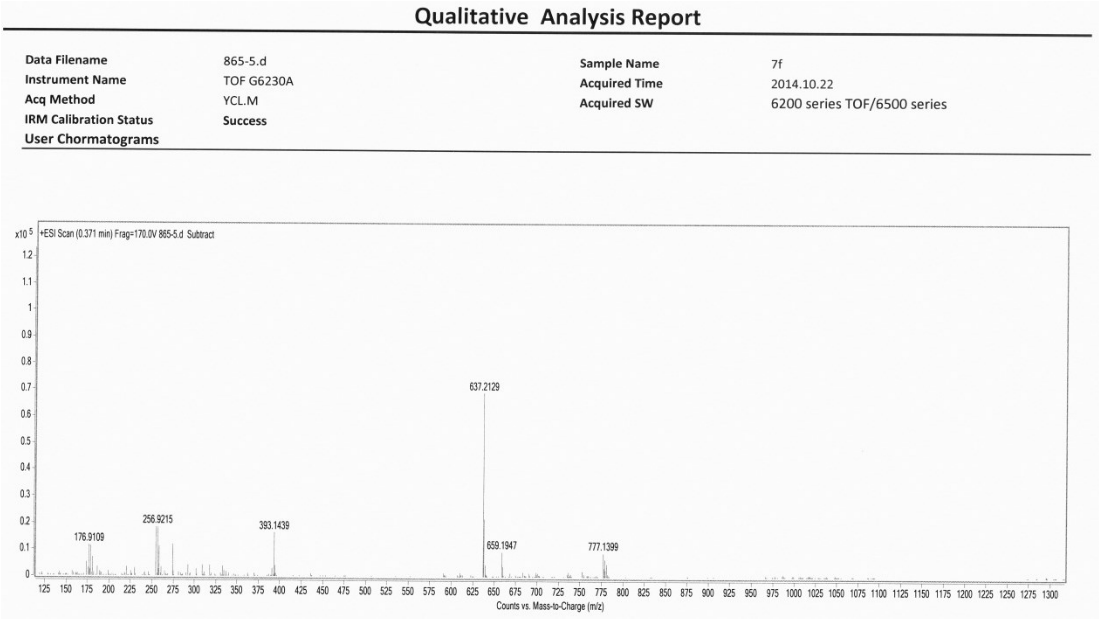

Supplement: Supplementary file 1 [file molecules-19-19718-s001.pdf]
